# Hospital accreditation: lessons from low- and middle-income countries

**DOI:** 10.1186/s12992-014-0065-9

**Published:** 2014-09-04

**Authors:** Helen Smits, Anuwat Supachutikul, Kedar S Mate

**Affiliations:** Institute for Healthcare Improvement, 20 University Road, 7th Floor, Cambridge, MA 02138 USA; The Healthcare Accreditation Institute, Bangkok, Thailand; Department of Medicine, Weill Cornell Medical Center, 525 E 68th Street, Box 130, New York, NY 10065 USA

**Keywords:** Innovation in accreditation, Quality improvement, Low and middle-income countries

## Abstract

The growth of accreditation programs in low- and middle-income countries (LMICs) provides important examples of innovations in leadership, governance and mission which could be adopted in developed countries. While these accreditation programs in LMICs follow the basic structure and process of accreditation systems in the developed world, with written standards and an evaluation by independent surveyors, they differ in important ways. Their focus is primarily on improving overall care country-wide while supporting the weakest facilities. In the developed world accreditation efforts tend to focus on identifying the best institutions as those are typically the only ones who can meet stringent and difficult evaluative criteria.

The Joint Learning Network for Universal Health Coverage (JLN), is an initiative launched in 2010 that enables policymakers aiming for UHC to learn from each other’s successes and failures. The JLN is primarily comprised of countries in the midst of implementing complex health financing reforms that involve an independent purchasing agency that buys care from a mix of public and private providers [Lancet 380: 933-943, 2012]. One of the concerns for participating countries has been how to preserve or improve quality during rapid expansion in coverage. Accreditation is one important mechanism available to countries to preserve or improve quality that is in common use in many LMICs today.

This paper describes the results of a meeting of the JLN countries held in Bangkok in April of 2013, at which the current state of accreditation programs was discussed. During that meeting, a number of innovative approaches to accreditation in LMICs were identified, many of which, if adopted more broadly, might enhance health care quality and patient safety in the developed world.

**Electronic supplementary material:**

The online version of this article (doi:10.1186/s12992-014-0065-9) contains supplementary material, which is available to authorized users.

## Introduction

Hospital accreditation was first developed in the United States by the American College of Surgeons close to 100 years ago [1]. Spread to other countries was slow at first but accelerated in the 1980s and 1990s, chiefly in English-speaking developed countries [2]. More recently, accreditation has been adopted in a number of low- and middle-income countries (LMICs) as a strategy to improve basic health service quality [3–8].

The acceleration of universal health coverage (UHC) in LMICs through insurance also appears to have accelerated the use of accreditation and accreditation-like systems [9]. Such systems are attractive to insurers as a way of defining which institutions may participate and which might receive bonus payments. Accreditation also appeals to governments seeking to provide UHC since it permits the use of independent professional surveys to ensure that financing for health care services is provided only to facilities that meet a high standard of care [10]. Accreditation appeals to health care facilities as it can provide external validation of quality in a setting where overall medical care is known to be highly variable [11]. Such proof can have an impact on consumer behavior, generating a new or increased income stream for accredited institutions as a result of the insurance support new patients bring with them.

Although well documented within selected LMIC countries, some of the most impressive schemes in these settings are little known to outsiders [12]. A careful look at these schemes and at some of the innovative approaches they have taken bears lessons not just for accreditation programs in LMICs, but also for the fields of health care quality and patient safety. Newer accreditation schemes in LMICs commonly have a very different sense of mission from similar efforts in developed countries: they assume responsibility for improving quality throughout the health care system in both accredited and non-accredited facilities [13]. In a wide variety of ways, from the design of their quality standards, reporting requirements for indicators, sponsored teaching programs in quality and safety, and annual meetings on quality strategies, they aim to improve all of the available in-country care.

The Joint Learning Network for Universal Health Coverage (JLN), is an initiative launched in 2010 that enables policymakers aiming for UHC to learn from each other’s successes and failures. The JLN is funded by the Rockefeller and Gates Foundations and overseen by a governing board of senior officials from the participating countries. JLN members are all in the midst of implementing complex health financing reforms that involve an independent purchasing agency that buys care from a mix of public and private providers [14]. Initiatives to improve quality have been one aspect of JLN activity; member countries expressed particular interest in learning more about accreditation systems. A multi-country workshop involving all active JLN members as of early 2013 was held in Bangkok in April of that year, sponsored by the Thai Healthcare Accreditation Institute with support from the Institute for Healthcare Improvement [14,15]. The ten countries are: Ghana, India, Indonesia, Kenya, Malaysia, Mali, Nigeria, the Philippines, Thailand, and Vietnam) [16]. These ten countries either have accreditation schemes in place or are developing them. Participating countries chose their own conference attendees; these included heads of accreditation agencies, senior staff from ministries of health, senior staff from insurers and representatives of relevant non-governmental organizations.

The aim of this paper is to report on how this group of countries is using accreditation with an emphasis on the innovative modifications of the accreditation structures as they are used in the developed world. We believe these hold lessons, which viewed in the context of recent discussion around “reverse innovation”, could have substantive and important implications for improving quality of health care in more developed countries as well [17]. Table 1 indicates the current status of accreditation programs in all participating countries; and Table 2 indicates the specific settings where these innovations might be implemented in other contexts.

**Table 1:** **Accreditation programs in attendees’ countries**

**Country**	**Current program**	**Structure & location**	**ISQua accredited**	**How is the accreditation program used by the UHC Insurance scheme?**
Ghana	Yes	Part of National Health Insurance Scheme (NHIS)	No	Claims reviewed; may be denied
India	Yes	Independent: National Accreditation Board of Hospitals	Yes	Varies by insurer; actively used.
Indonesia	Yes	Independent: Indonesia Accreditation Body	No	Not reported
Kenya	Yes	Part of National Hospital Insurance Fund (NHIF)	No	Required*
Malaysia	Yes	Independent: Malaysian Society for Quality in Health	Yes	Not reported
Mali	Yes; ambulatory only	Part of Ministry of Health: National Hospital Assessment Agency	No	Not reported
Nigeria	Yes	Part of National Health Insurance Scheme	No	Required*
Philippines	Yes	Part of Philippine Health Insurance Corporation	No	Required*
Thailand	Yes	Independent with Ministry of Health linkages: Healthcare Accreditation Institute	Yes	Required*
Vietnam	Under development	Part of Ministry of Health	No	Not yet fully implemented

Note: This table was prepared from presentations made in May of 2013; “not reported” means that the presenter did not mention any UHC/Accreditation link.

*Required = Insurers require that facilities participating in the insurance scheme be accredited by the National Accreditation program.

**Table 2:** **Potential use of reverse innovation in accreditation systems**

**Innovation**	**Countries where used**	**Applicability**
Focus on entire system, not just best facilities.	Thailand, Malaysia, India, Kenya, Ghana	Any setting with variable quality
Indicator use very focused, requirements of accrediting body, insurer and government fully harmonized	Malaysia, India	Could benefit state innovation projects in US
Accreditation used as a “brand” for ambulatory facilities; linked to community education	KMET in Kenya	Any setting where improvement in the ambulatory setting is desirable
“Graded” accreditation used to ensure that facilities which do not fully pass are supported in improving.	India; reported from other countries such as Brazil	Any country where not all facilities are accredited
Surveys used to identify facility need; resources such as loans provided where appropriate	Kenya, Ghana (Pharmaccess)	Any country where capital resources are constrained
Surveys used as teaching opportunity	Thailand, Malaysia	All accreditation settings

## Background and definitions

For this paper we use the International Society for Quality in Healthcare’s (ISQua) definition of accreditation: “A public recognition by a healthcare accreditation body of the achievement of accreditation standards by a healthcare organization, demonstrated through an independent external peer assessment of that organization’s level of performance in relation to the standards” [18]. We define empanelment to mean an insurance company-driven process of surveying and accepting institutions and certifying aspects of their care delivery system. Where the definitions intersect, as they sometimes do, we have accepted the term used by the local organization.

The International Society for Quality in Healthcare serves as the principal international body on accreditation in health care [19]. They “accredit the accreditors” by issuing standards and surveying in three areas: health care standards, external evaluation organizations, and surveyor training programs [19–22]. Although the majority of ISQUA-accredited organizations are in the developed world, three of the countries attending the Bangkok meeting have at least one element of ISQua approval (Thailand, Malaysia and India) [19].

Despite their growing popularity, accreditation programs have been criticized for being too rigid, for failing to be sufficiently objective in the survey process and, most important, for not having a measurable impact on the quality of care [23]. Reliable studies of quality are difficult to perform but appear to show that care processes improve; it has been harder to demonstrate that improved outcomes of care result. One review suggests that there is a demonstrable impact of specialty accreditation, such as in chest pain management, sleep medicine and trauma management [13]. Concerns about rigidity have raised questions about whether accreditation actually works against efforts to improve quality [24]. A commentary published by the Agency for Healthcare Research and Quality suggests that accreditation can improve the minimum acceptable level of care but is unlikely, in and of itself, to lead to excellence [25].

All accreditation programs have at least four main elements, each of which has been fertile ground for innovation in LMIC settings. These include:

1. The development of an organization in which accreditation efforts are housed.
2. The development of standards and the accompanying specific criteria.
3. The implementation of the survey process, including hiring and training surveyors, and scoring and reporting the results.
4. Incentives/disincentives and institutional support (i.e., what is done with the results to encourage or require improvement).

### Organizational Structure

Before standards can be written or surveyors hired, an organization must be identified as the accrediting body. Historically these have been free-standing entities, usually developed by a professional body or a coalition of professional bodies, as was the case in developing the first two accreditation agencies in the United States and Canada [26]. In LMICs, however, professional organizations may not have the resources or financial capacity to field a major new effort. As a result, accreditation has, in some settings, been housed within the government or major insurer. For example, Ghana’s National Health Insurance Scheme originally placed responsibility for accreditation within government; that task is now being transferred to an independent body [27]. In Kenya, the National Health Insurance Fund (the insurer) manages accreditation; their standards, known as the Kenya Quality Model, were developed by a broad coalition of professionals outside of the Insurance Fund and are supported by the Ministry of Health [28]. These arrangements allow the early development of accreditation to take place without all the challenges inherent in establishing a new independent body.

In all organizational structures, representatives at the Bangkok conference emphasized the importance of engaging all stakeholders in accreditation. This contrasts with accreditation in the United States, where the majority of the Joint Commission Board members are physicians who represent national professional organizations, with fewer members from the American Hospital Association, the professional associations of nursing and allied clinical services, and patient/public members [29]. No representatives of government sit on the Joint Commission Board even though Commission accreditation is used to qualify hospitals for participation in Medicare and Medicaid. In Malaysia, by contrast, the accrediting body was formed in collaboration with the Ministry of Health, the Private Hospital Association, and the Medical Association (see Additional file for more detail) [30]. The presence of government as participants at the Board level with the ability to influence policy decisions (although not individual accreditation decisions) makes for a very different working environment from that of the Joint Commission, where all contact with government is handled by staff and where important policy changes are made without government representatives present. Similar multi-stakeholder organizational structures, with adaptation to suit local needs, are found in the other well developed programs in India and Thailand.

### Standards

Any analysis of an accreditation process needs to start with the standards against which health facilities are being accredited [31]. If these standards focus on static, structural elements such as job descriptions and the presence of fire extinguishers but neglect clinical care, then rigor in the accreditation process will have little meaning. ISQua now emphasizes the development of what are termed “quality improvement” standards that require institutions to improve performance [32]. These standards include structural requirements such as the creation of QI committees as well as process requirements such as requiring the reporting of the outcomes of specific patient conditions. The use of standards that support quality improvement initiatives is not unique to LMIC countries, but the focus on them is. There was a clearly expressed desire in all countries attending the meeting to use standards to improve overall quality of care—not just to sort hospitals into those that “pass” an accreditation visit and those that don’t. Institutions that fail to meet standards are, in many LMIC settings, the only available source of care for parts of the population. Upgrading their care is therefore a high priority.

One particularly interesting approach, expressed by both Malaysia and Thailand, was to begin with relatively achievable accreditation standards coupled with a commitment to continue upgrading requirements over time—that is, “start with what we have.” In this context, Malaysia has recently issued the fourth version of its hospital standards since the program was initiated in 1999. Thailand has also made progressive changes, introducing into the accreditation process a stepwise recognition program in 2004 and patient safety goals in 2006 (See Additional file 1, Leadership and Strategy for Improvement (Thailand) and Additional file 2, Malaysian Health Sector for more information).

Another important innovation is happening in Malaysia where the accreditor now requires reporting of a variety of specific patient safety and quality of care indicators, for example the number of ventilator-associated pneumonias (VAPs). Such reporting is not routine across other developed country accreditation programs. All public hospitals in Malaysia must report on these indicators in order to maintain their accreditation status. All reporting hospitals receive training in the “care bundle” for patients on ventilators and Malaysia has been able to show a marked reduction in the number of VAPs over a five-year period [33]. They reported similar progress in the reduction of peri-operative infections (See Additional file 2 for more detail) [30]. The focus on a limited number of high impact indicators is very different from the approach in the developed world where efforts to use indicators in a comprehensive fashion may dilute the impact of the most important.

Another example of both standards development and indicator use is currently being developed in India, where the National Accreditation Board of Hospitals (NABH) has to date been able to accredit relatively few hospitals within this vast country [34]. UHC in India is the responsibility of individual states, resulting in very large government-sponsored insurance companies being developed on a state-by-state basis. Recently the Aarogyasri Health Care Trust, the insurer to more than 65 million people in the state of Andhra Pradesh, began work on identifying standards and indicators that will be required of all hospitals empaneled to provide care to their beneficiaries. The Trust is working with NABH to ensure that their standards and indicators are consistent with those required of accredited hospitals, with the expectation that this consistency will both improve care in the short term and encourage more hospitals to achieve accreditation. With support from the World Bank, insurers from several states are meeting on a regular basis to co-develop a shared set of standards and indicators that encourage hospitals to move towards a harmonized system of providing higher quality service delivery [35]. The hope is also that this will lessen the reporting burden and streamline the number of quality measures—something that is sorely needed in the “developed” world, where measures of quality have multiplied. The current test case for this work is focused on neonatal care delivery and the first patient-level results should be available later in 2014.

### Implementation

The elements of accreditation implementation typically include hiring and training surveyors, scheduling accreditation visits, developing a scoring system for the elements in the standards, and categorizing and reporting the results.

The areas where LMIC approaches to implementation of accreditation are most strikingly different from established programs are in the scoring, categorizing, and reporting elements. A wide variety of methods have been developed to bring institutions gradually into compliance with accreditation standards, affording the weaker ones the time required to improve. India’s NABH, for example, uses a single set of standards but, depending on the results, gives three different categories of awards: pre-accreditation entry level, pre-accreditation progressive level, and fully accredited. Institutions in either of the pre-accreditation levels are given a specific time frame within which to apply for re-surveying and possible full accreditation [36].

Another approach to staging implementation can be found in the SafeCare standards, developed by a Dutch NGO, Pharmaccess, Joint Commission International (US), and COHSASA (South Africa), and used in Ghana and Kenya (as well as other countries) to survey basic, resource-poor facilities. The SafeCare methodology dissects the improvement process of small health care institutions into measurable steps. An improvement trajectory is created that provides positive incentives for health care institutions to move upwards in quality, ultimately to the level that qualifies them for full accreditation. A guided, stepwise process for achieving accreditation is designed to boost client, investor, and regulator confidence in the motivation and capacity of these institutions to steadily enhance their performance.

Rapid reporting of accreditation survey results to institutions in LMIC settings is essential and is another area of innovative activity by accreditors in these settings. Delay in reporting results was identified as one of the elements that doomed the failed accreditation efforts in Zambia [37]. Pharmaccess, in implementing the SafeCare standards, uses mobile devices to score survey results, enabling them to provide the facility with survey results at the end of the visit [38]. In Malaysia, the survey team conducts nightly meetings while on site to identify areas of strengths and weaknesses in preparation for the exit conference. This allows the exit conference to clearly indicate areas of commendation and areas for further improvement. The final decision about accreditation, however, must go through further review by a group of councilors before accreditation status is announced and the certificate is conferred on the organization.

### Incentives and Monitoring

There are a wide variety of incentives and disincentives available, based on the outcomes of accreditation efforts, including financial rewards, public reporting of results as a way to attract more patients to the best facilities, and the provision of on-site support for weaker facilities.

The expanded health care financing made available under UHC schemes makes it possible to develop financial incentives such as additional payment per case for good performance, or a general bonus for good performance. In India, hospitals at all three NABH accreditation levels are paid more than non-accredited hospitals by certain health plans [39]. Similar incentives are either in place or being considered in other countries. In addition, some UHC insurance schemes have collaborated with accreditation agencies to fast-track payment for accredited institutions, thus providing another strong motivator to seek formal accreditation status since payment delays and the resultant cash flow challenges are a common complaint in LMICs.

Clear and public indication that a facility provides high-quality care can also, as noted earlier, have a financial effect by attracting more patients. In some of the countries represented at the Bangkok meeting, “gold star” status conferred by an accreditation program may also be a way to attract “medical tourists”—individuals from other countries seeking good care at a reasonable price. Tiered accreditation models using “gold star” or other rating systems for hospitals are used in Egypt, Brazil and Mexico among other countries allowing the best hospitals to advertise their expertise to nationals and international customers [40]. Medical tourists are not a part of country-based UHC efforts, but accreditation programs designed to support UHC quality can have corollary benefits for the institutions.

Many of the participants at the Bangkok workshop reported widespread concern about the quality of care in the facilities and services covered under UHC insurance schemes. Accreditors and insurers working together with healthcare providers have created innovative ways to align incentives and motivations in LMIC settings that may be instructive for developed countries. Interested in ‘paying for quality,’ insurers have agreed to providing greater financial reward to those systems passing accreditation standards. This has set off a virtuous cycle whereby accreditors benefit as there is added motivation for providers to become ‘accredited’; providers benefit from greater reimbursement if they are successfully accredited; insurers benefit as they can claim to have some independent assessment of healthcare quality; and the public benefits, from a higher quality health service overall and from the availability of information about which facilities have met accreditation standards.

One interesting manifestation of this virtuous cycles between accreditation, healthcare financing and provider systems that is emerging in developing countries is within the use of social franchises. KMET is an indigenous NGO in Kenya that surveys small outpatient facilities and “brands” those that do well as part of a quality system [41]. KMET provides training and microfinancing solutions for facilities that want to improve and achieve KMET “brand” status. KMET also works in the community to improve health-seeking behavior and to describe the “brand” and expectations of a KMET-affiliated clinic. Clinics are motivated to participate and improve their services due to the brand-following that KMET has created in rural villages. Since the National Hospital Insurance Fund (NHIF), the major insurer in Kenya, does not have the capacity to undertake multiple surveys of smaller facilities and clinics, they are now working with KMET to ensure integration of their different approaches. Both KMET and NHIF base their standards on the Kenya Quality Model.

Another example of providing incentives is PharmAccess, which supplies affordable loans for facility improvement, as part of their stepwise recognition of improvement systems, to assist facilities in overcoming resource deficiencies that prevent them from achieving accreditation. Fines for poor performance have also been used in many settings, but this approach raises the troubling issue of decreasing the resources available at the institutional level to help provide good care. The Bangkok meeting attendees preferred positive incentives. A comprehensive list of financial incentives and “disincentives” can also be found in the ISQua Toolkit [20].

## Potential impact and benefits

Accreditation is a complex social, regulatory, and organizational intervention, which can be improved over time. The development of Joint Commission standards, which evolved from a single page of standards in 1918 into today’s elaborate process that includes the reporting of outcomes, demonstrates both change and growth in the approach to accreditation in the United States. In an era of continuous improvement in the health care delivery process, a strong foundation for the continuous improvement of accreditation is needed. Unfortunately, no comprehensive review of accreditation systems in the developed world exists. In recent years, criticism of the Joint Commission and other older systems has focused on their rigidity and the fact that “external review was seen as negative and punitive rather than encouraging striving for improvement” [24]. Modern accreditation systems have struggled to move towards models that support QI but are hindered by their extensive existing standards which are difficult to eliminate without appearing to reduce the oversight of hospitals. Countries that began accreditation over the past 15–20 years have had the opportunity to focus more exclusively on improvement and to target their efforts on the areas of most concern.

The innovations in accreditation in LMIC settings described in this paper are very encouraging, but more work is needed to better characterize, study, and understand their potential for impact. Table 2 shows the potential uses of innovation in settings other than the ones described here. Improvement in accreditation will depend, in part, on the availability of studies that evaluate the elements of accreditation schemes, describing what has been successful in terms of both implementation and health care outcomes. Studies of the component parts of accreditation schemes are needed. Results of such studies will be of more value to those initiating accreditation efforts than existing summative evaluations about whether or not accreditation processes “work” [42,43].

Specific elements of accreditation such as standards design, use of feedback, indicator requirements, survey interval and structure all deserve further study. The details of weighting and scoring systems are, for example, almost completely absent from the literature [44]. Publication and discussion of scoring and weighting schemes would be a valuable resource for countries that are in early stages of accreditation development as well as for well-established accreditation programs seeking to improve themselves. Information about how best to identify and publicly report the level of quality in all facilities is also needed, as are studies of the impact of benchmarking on consumer behavior.

A comprehensive, web-based, free repository of all available ideas and evidence is another critical element needed to further our understanding about which features of accreditation work best and in which settings. All ten countries participating in the Bangkok meeting expressed eagerness to review standards developed elsewhere; we were both surprised and disappointed to discover that relatively few accreditation standards are available for review without cost. A collection of accessible standards from a variety of countries, with commentary on which aspects appear to work best, would be of great value and can save LMICs both time and money as they establish accreditation systems anew or refresh existing standards sets. A few sets of international standards are available without cost, including the country-specific standards described in this paper (see Additional file 3 for the Thai standards) and those of SafeCare and Det Norske Veritas (DNV) Healthcare. Standards from the Joint Commission and Joint Commission International must be purchased, as must the standards from many important and well-established accreditation programs, including those in Canada, Australia, and South Africa. An arrangement similar to that of many peer-reviewed publications, which makes standards available at no cost to LMICs, would be very welcome. A repository maintained by an organization like the World Health Organization or ISQua would be ideal, especially if comments by other users could be added. Other elements of the accreditation process, such as training materials for surveyors, would be welcome additions to such a compendium of information.

LMICs can contribute to the dissemination of information about accreditation by reporting their approaches in international publications. A number of the innovations mentioned in this paper are of sufficient interest to warrant detailed publication. Since the organizational structure of accreditation varies across countries, formal comparative effectiveness study may be difficult. A useful start would be a thorough description of all organizational models, with detailed qualitative data on those that appear to work best.

Hospital accreditation is in a period of rapid international expansion. To achieve the potential results that all of its supporters want, far more information should be publicly available. At present the developers of a new accreditation scheme are often left to reinvent the wheel in fundamental areas such as the development of standards, the training of surveyors and the use of incentives. A careful re-examination of the business model of successful schemes in the developed world and a commitment to reporting and sharing information could help this important field move forward quickly.

## Additional files

Additional file 1:
**Leadership and Strategy for Improvement (Thailand).**
Additional file 2:
**Malaysian Health Sector.**
Additional file 3:
**Hospital Standards, Thailand.**
