# Supplementary material for: Hospital accreditation: lessons from low- and middle-income countries
Source: Global Health. 2014 Sep 4;10:65. doi: 10.1186/s12992-014-0065-9 (PMC4159532; doi:10.1186/s12992-014-0065-9)
Supplement: Additional file 1: — Leadership and Strategy for Improvement (Thailand). [file 12992_2014_65_MOESM1_ESM.pptx]

## Slide 1
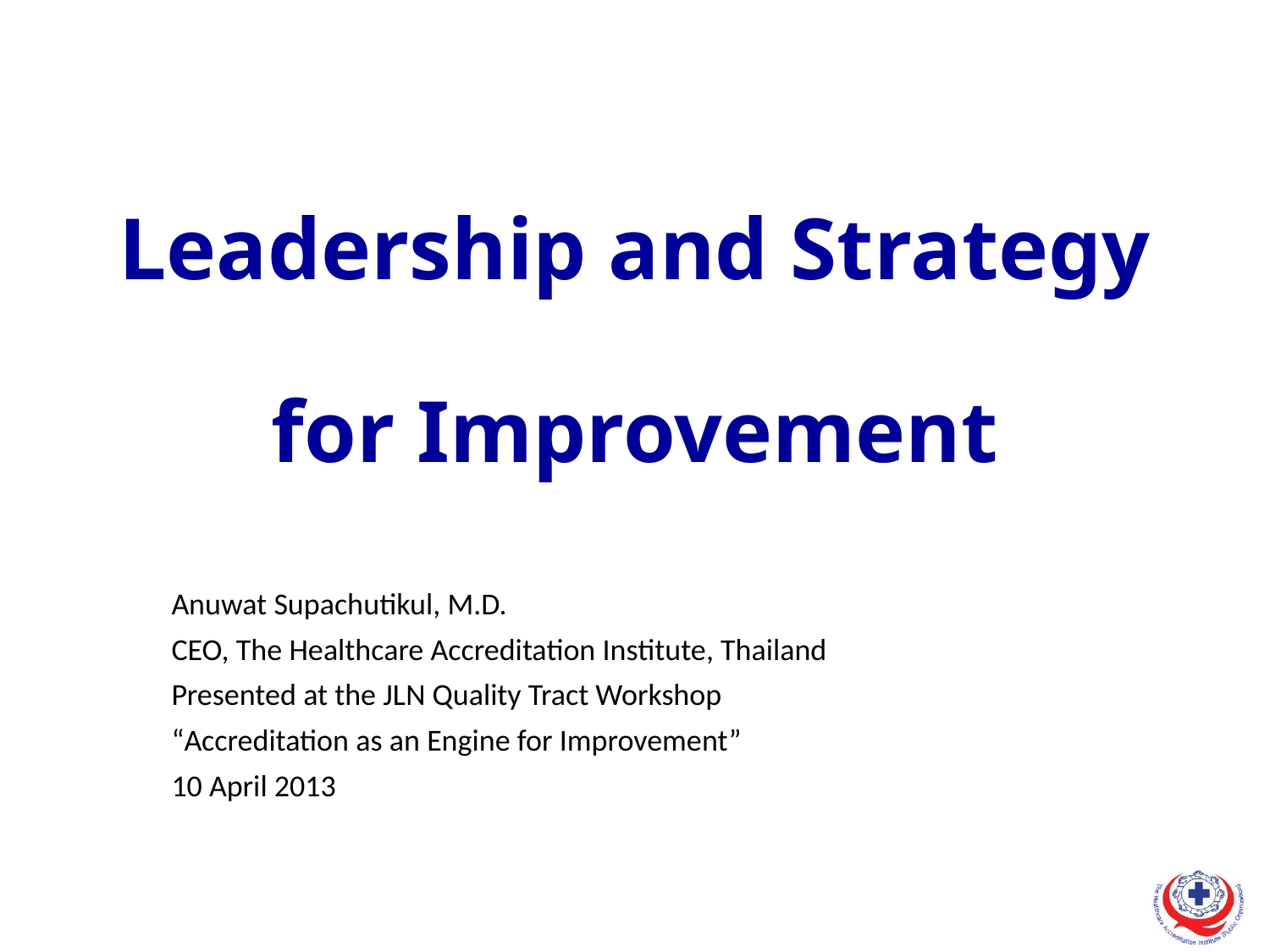

# Leadership and Strategy for Improvement
Anuwat Supachutikul, M.D.
CEO, The Healthcare Accreditation Institute, Thailand
Presented at the JLN Quality Tract Workshop
“Accreditation as an Engine for Improvement”
10 April 2013

## Slide 2
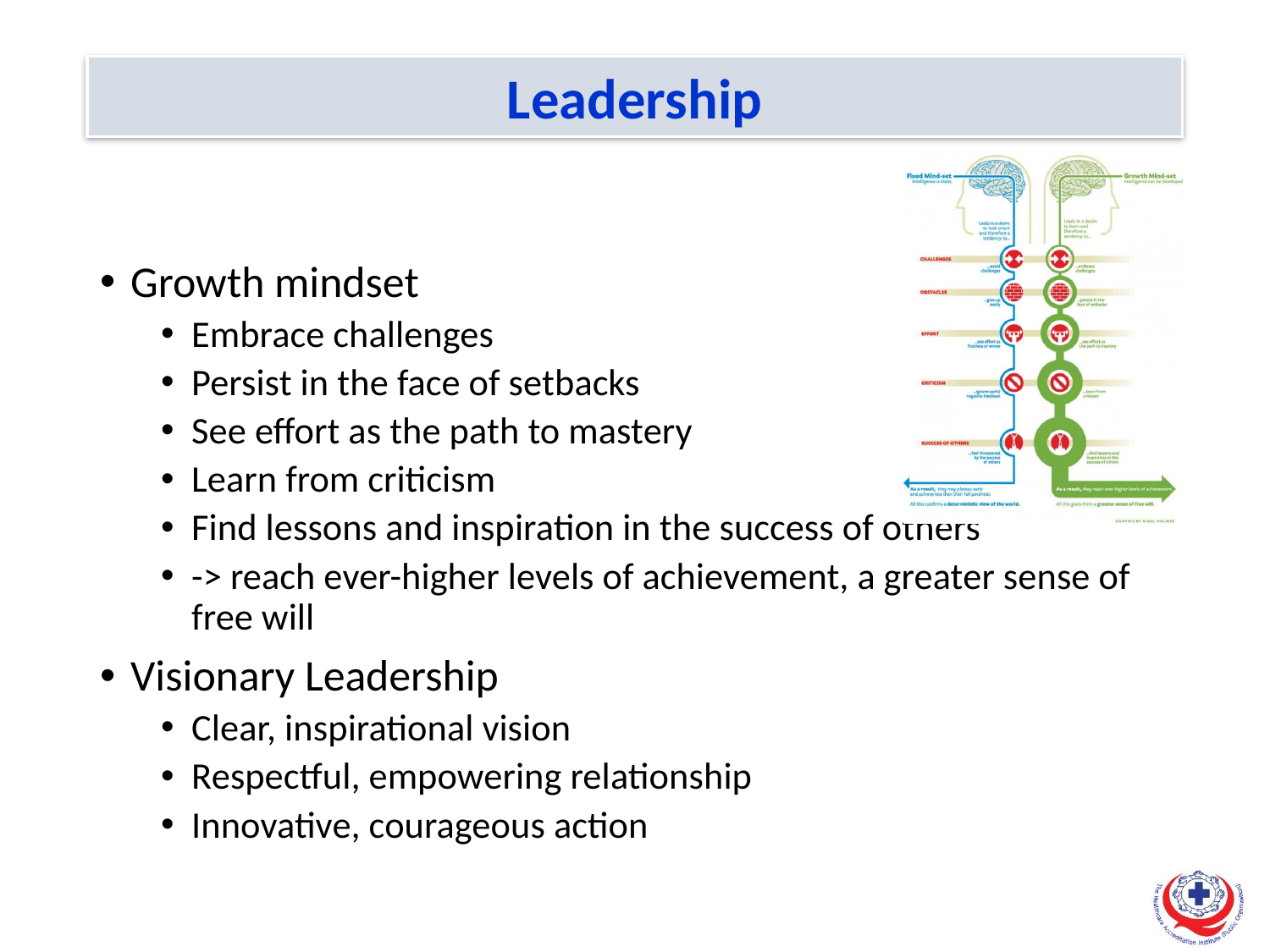

Leadership
Growth mindset
Embrace challenges
Persist in the face of setbacks
See effort as the path to mastery
Learn from criticism
Find lessons and inspiration in the success of others
-> reach ever-higher levels of achievement, a greater sense of free will
Visionary Leadership
Clear, inspirational vision
Respectful, empowering relationship
Innovative, courageous action

## Slide 3
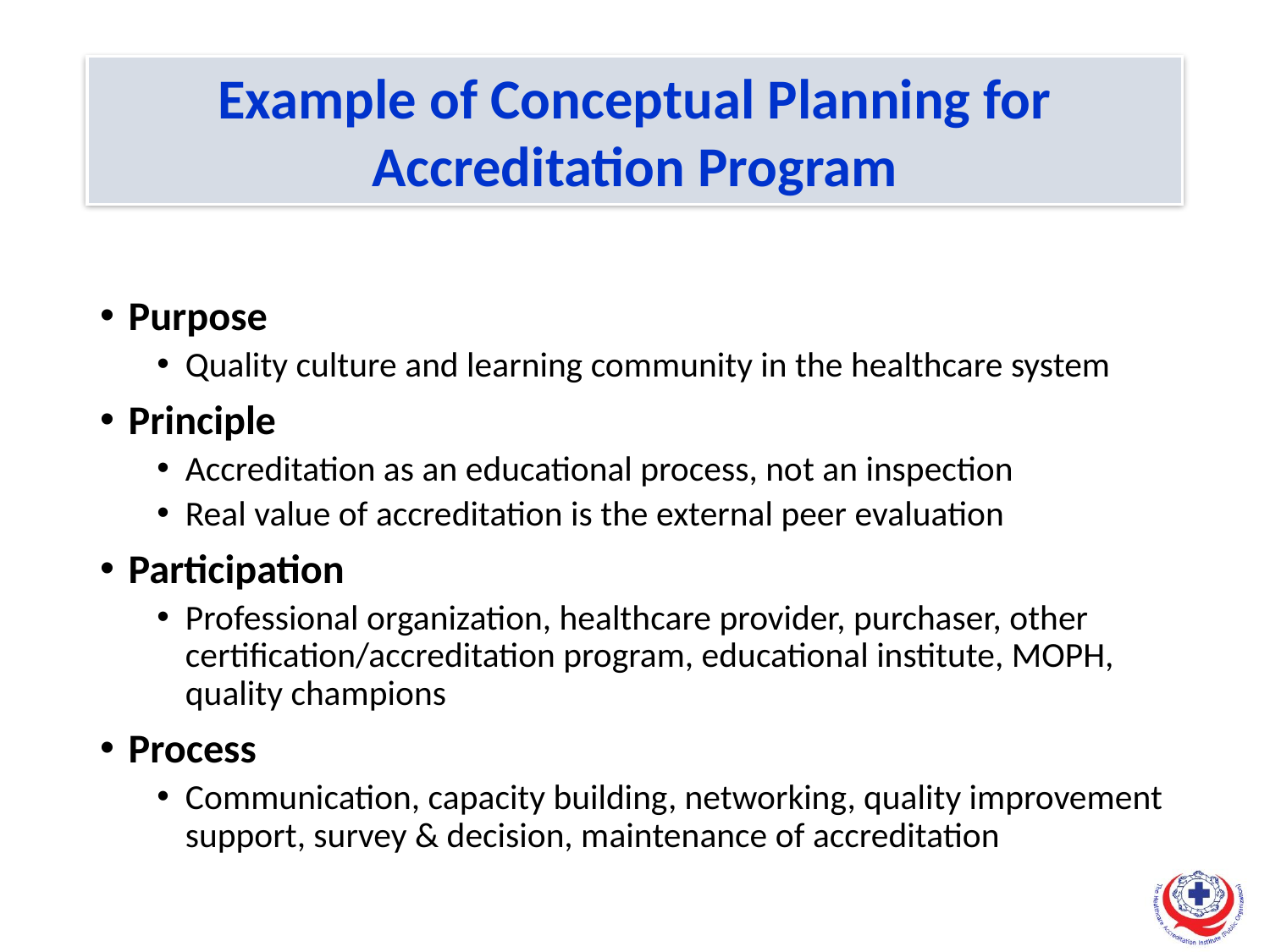

Example of Conceptual Planning for Accreditation Program
Purpose
Quality culture and learning community in the healthcare system
Principle
Accreditation as an educational process, not an inspection
Real value of accreditation is the external peer evaluation
Participation
Professional organization, healthcare provider, purchaser, other certification/accreditation program, educational institute, MOPH, quality champions
Process
Communication, capacity building, networking, quality improvement support, survey & decision, maintenance of accreditation

## Slide 4
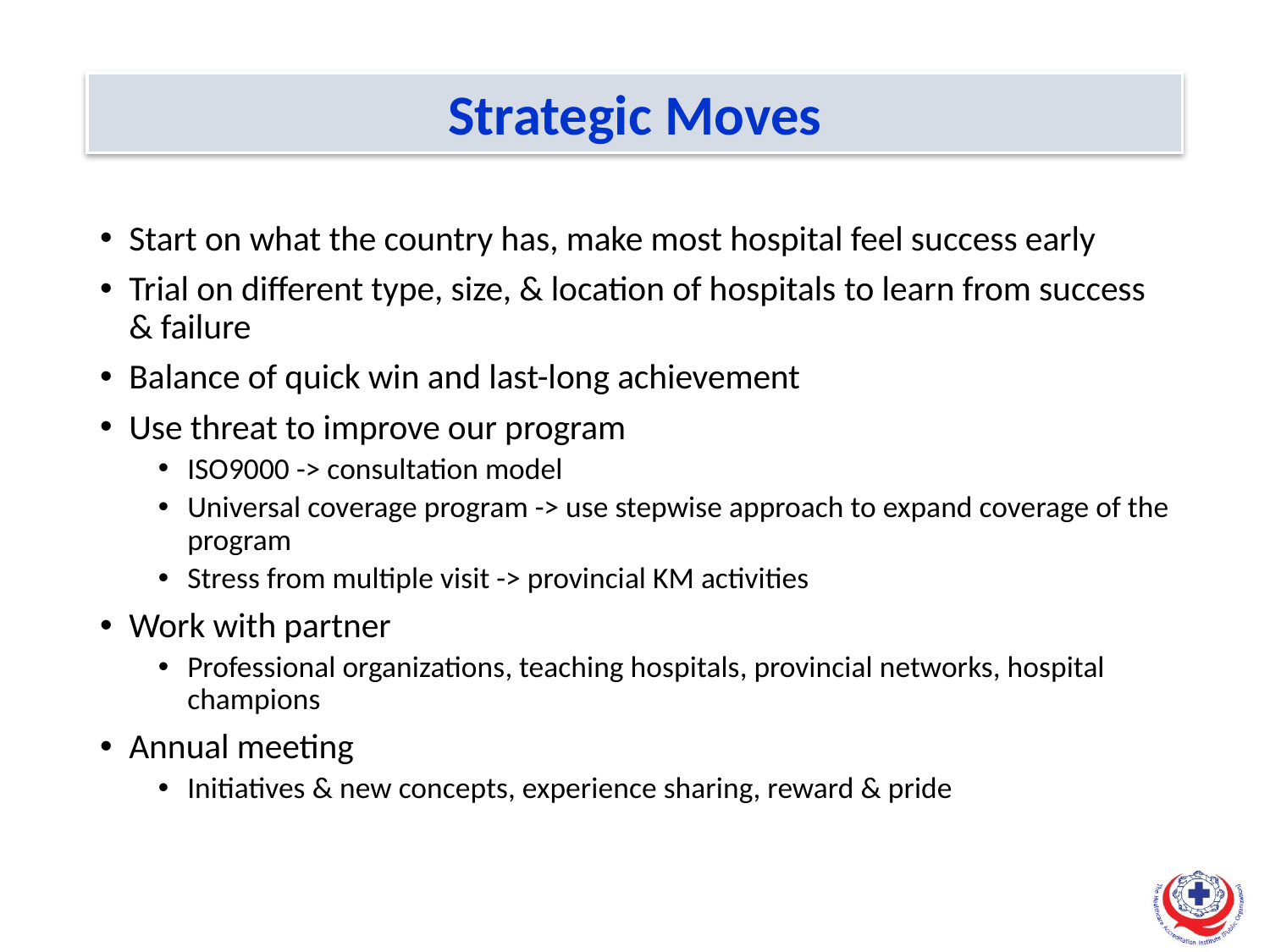

Strategic Moves
Start on what the country has, make most hospital feel success early
Trial on different type, size, & location of hospitals to learn from success & failure
Balance of quick win and last-long achievement
Use threat to improve our program
ISO9000 -> consultation model
Universal coverage program -> use stepwise approach to expand coverage of the program
Stress from multiple visit -> provincial KM activities
Work with partner
Professional organizations, teaching hospitals, provincial networks, hospital champions
Annual meeting
Initiatives & new concepts, experience sharing, reward & pride

## Slide 5
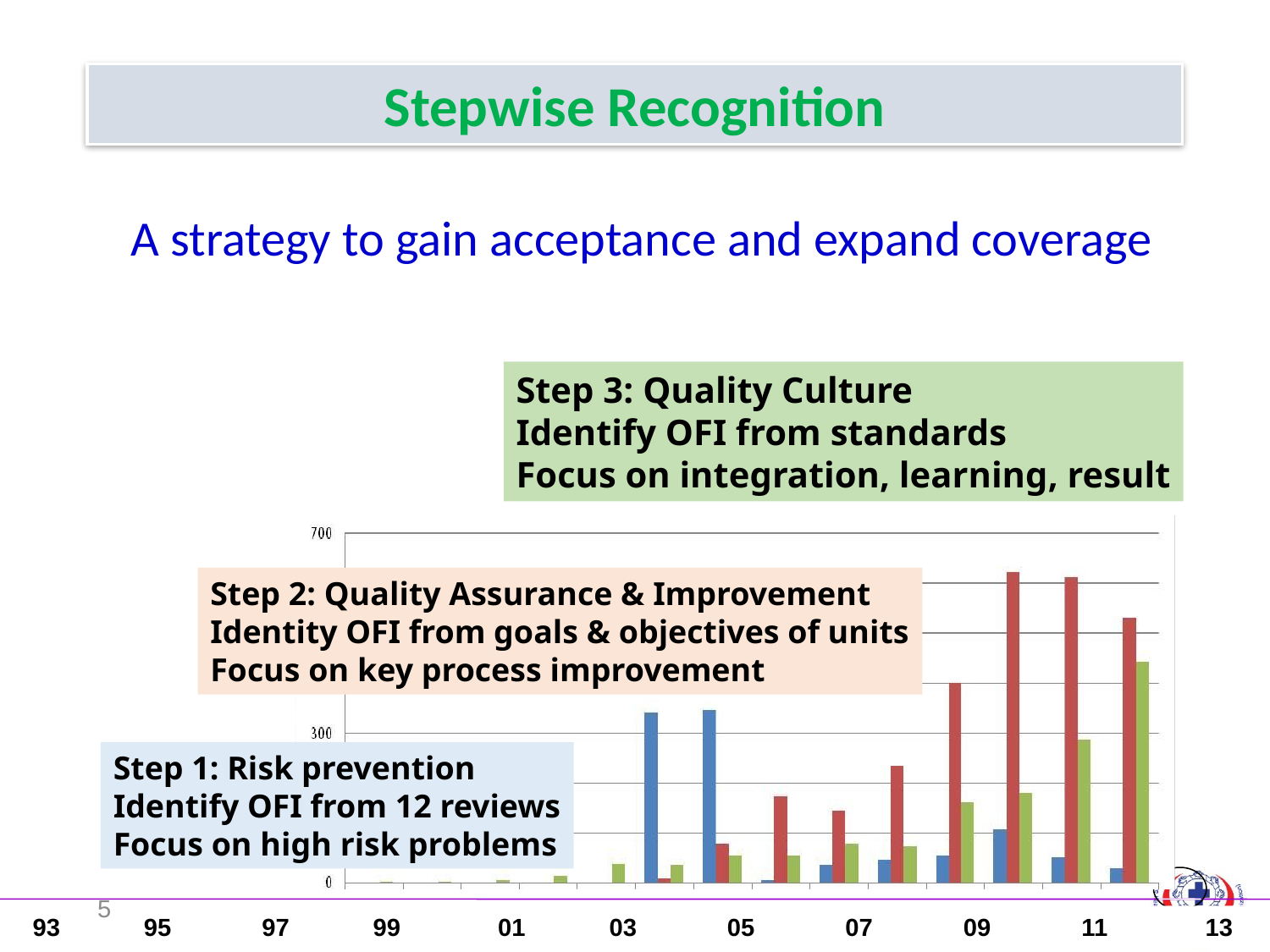

Stepwise Recognition
A strategy to gain acceptance and expand coverage
Step 3: Quality Culture
Identify OFI from standards
Focus on integration, learning, result
Step 2: Quality Assurance & Improvement
Identity OFI from goals & objectives of units
Focus on key process improvement
Step 1: Risk prevention
Identify OFI from 12 reviews
Focus on high risk problems
5
93 95 97 99 01 03 05 07 09 11 13

## Slide 6
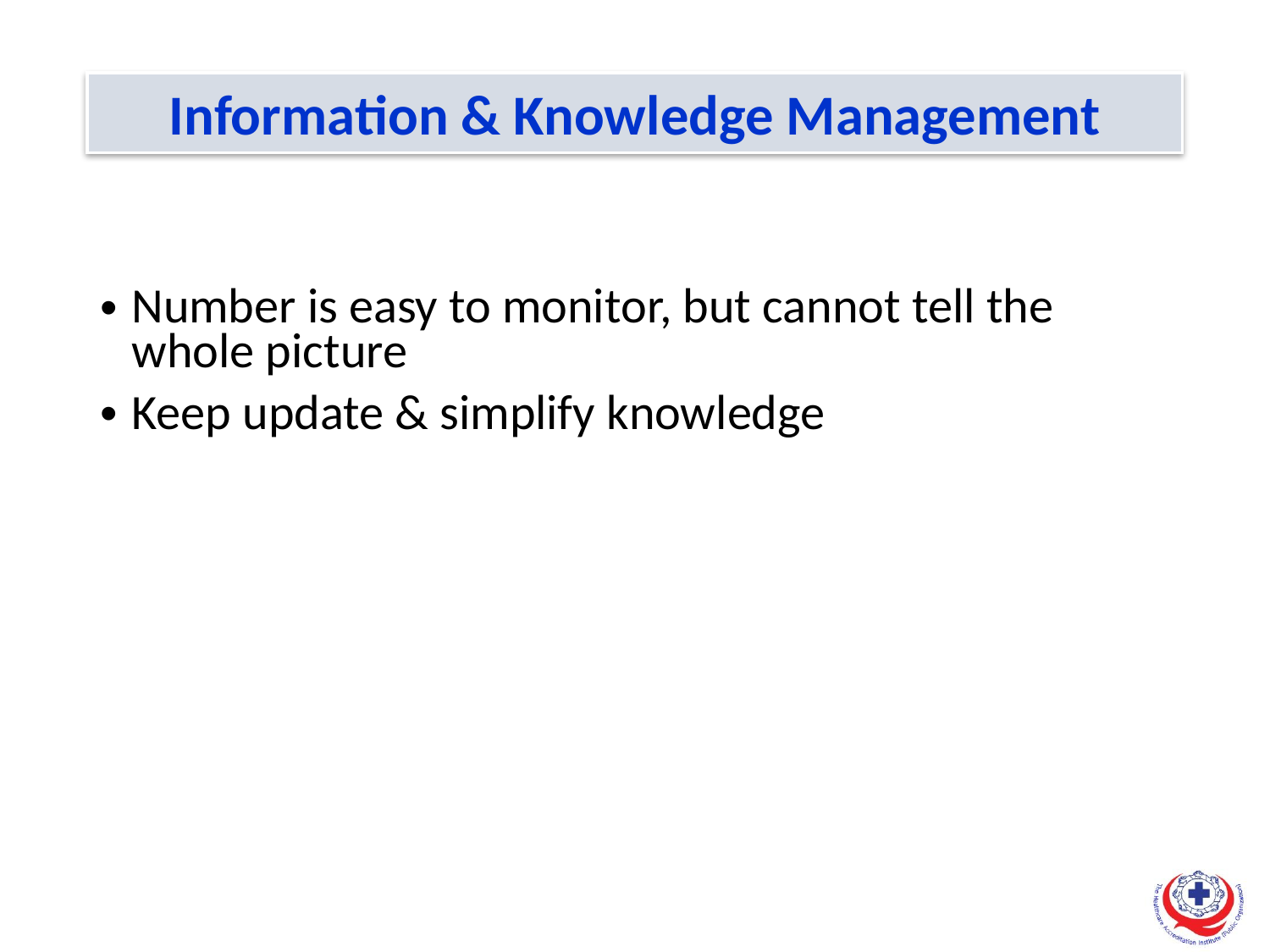

Information & Knowledge Management
Number is easy to monitor, but cannot tell the whole picture
Keep update & simplify knowledge

## Slide 7
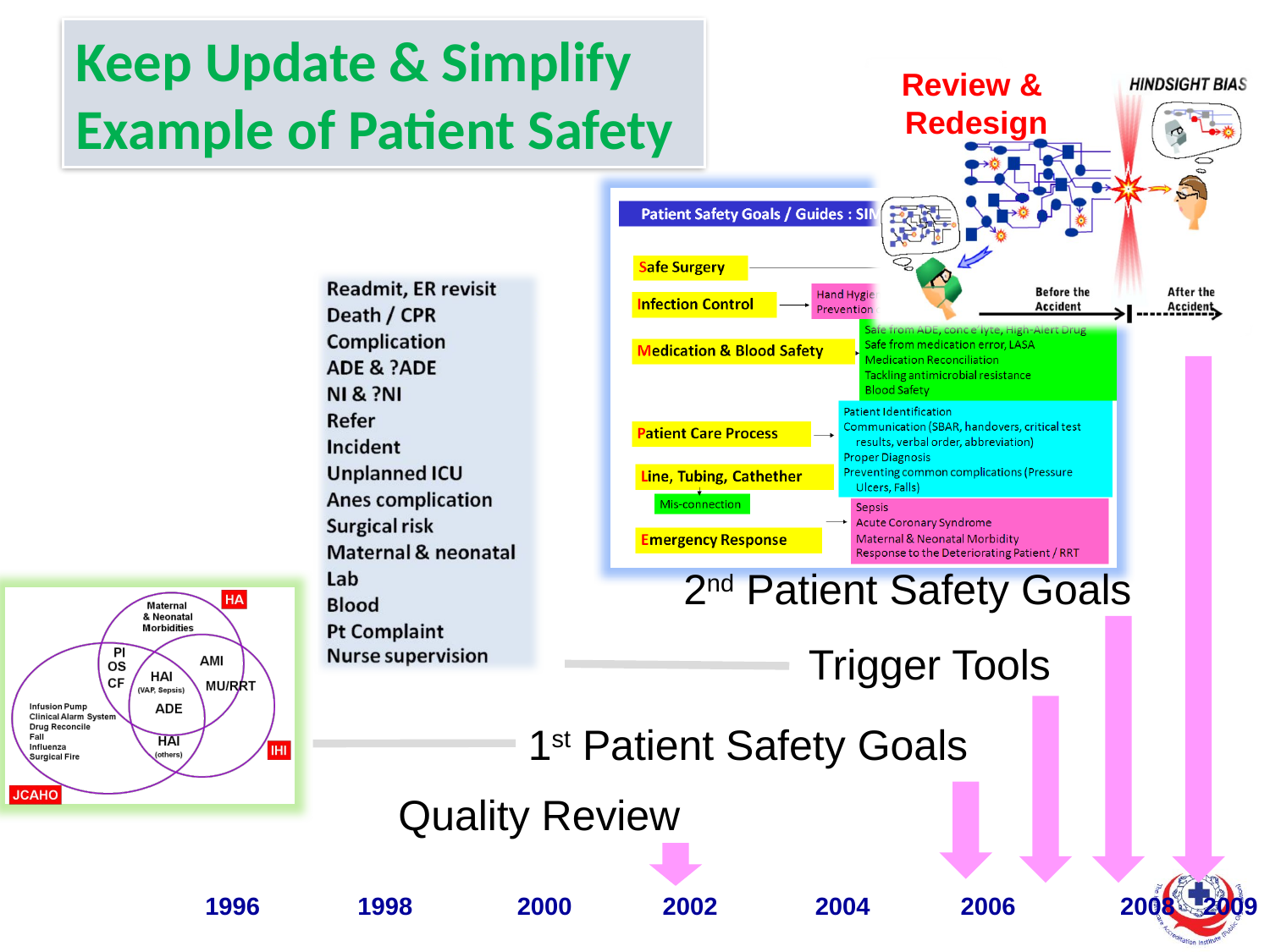

Keep Update & Simplify
Example of Patient Safety
Review &
Redesign
2nd Patient Safety Goals
Trigger Tools
1st Patient Safety Goals
Quality Review
1996 1998 2000 2002 2004 2006 2008 2009

## Slide 8
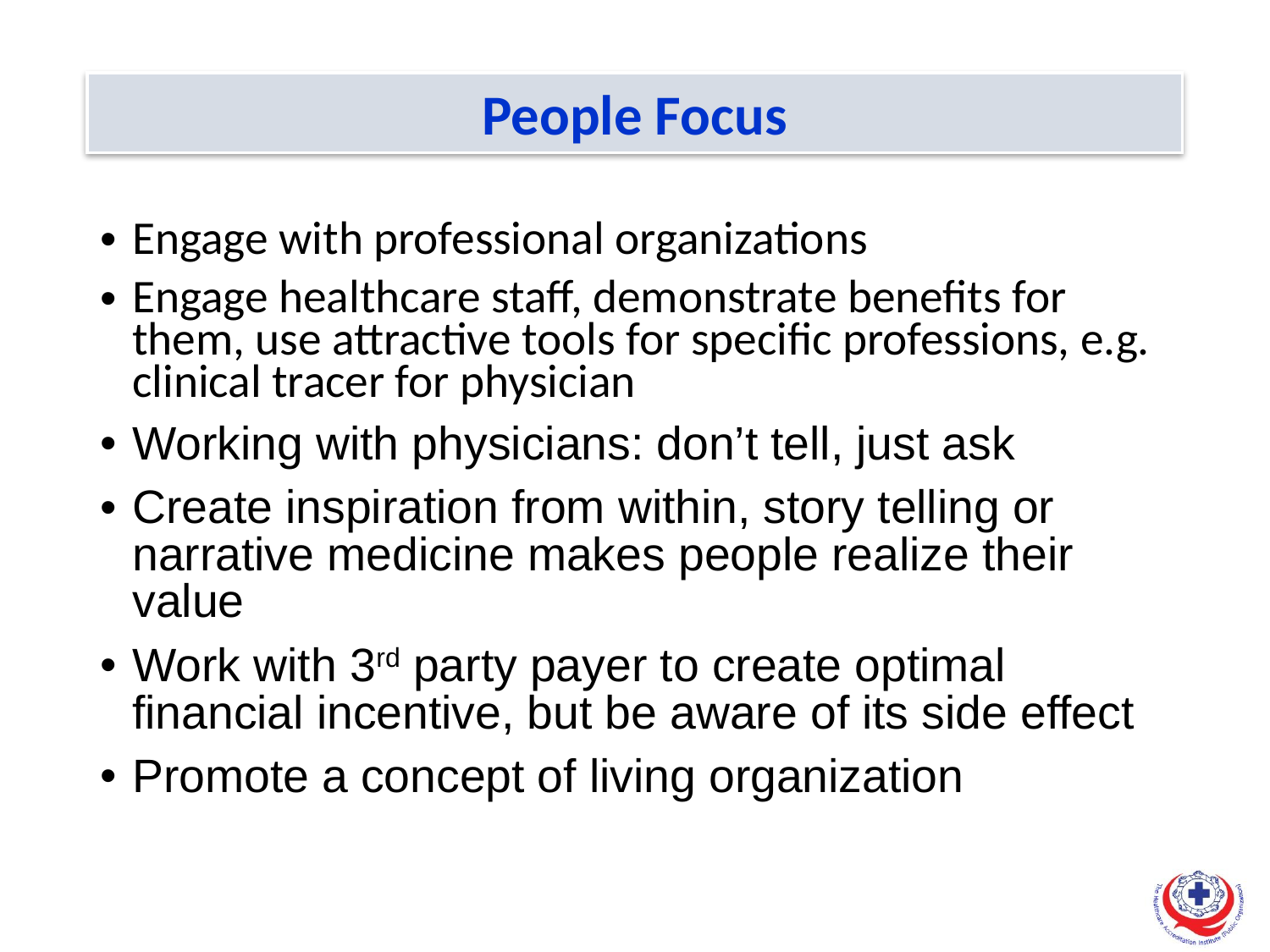

People Focus
Engage with professional organizations
Engage healthcare staff, demonstrate benefits for them, use attractive tools for specific professions, e.g. clinical tracer for physician
Working with physicians: don’t tell, just ask
Create inspiration from within, story telling or narrative medicine makes people realize their value
Work with 3rd party payer to create optimal financial incentive, but be aware of its side effect
Promote a concept of living organization

## Slide 9
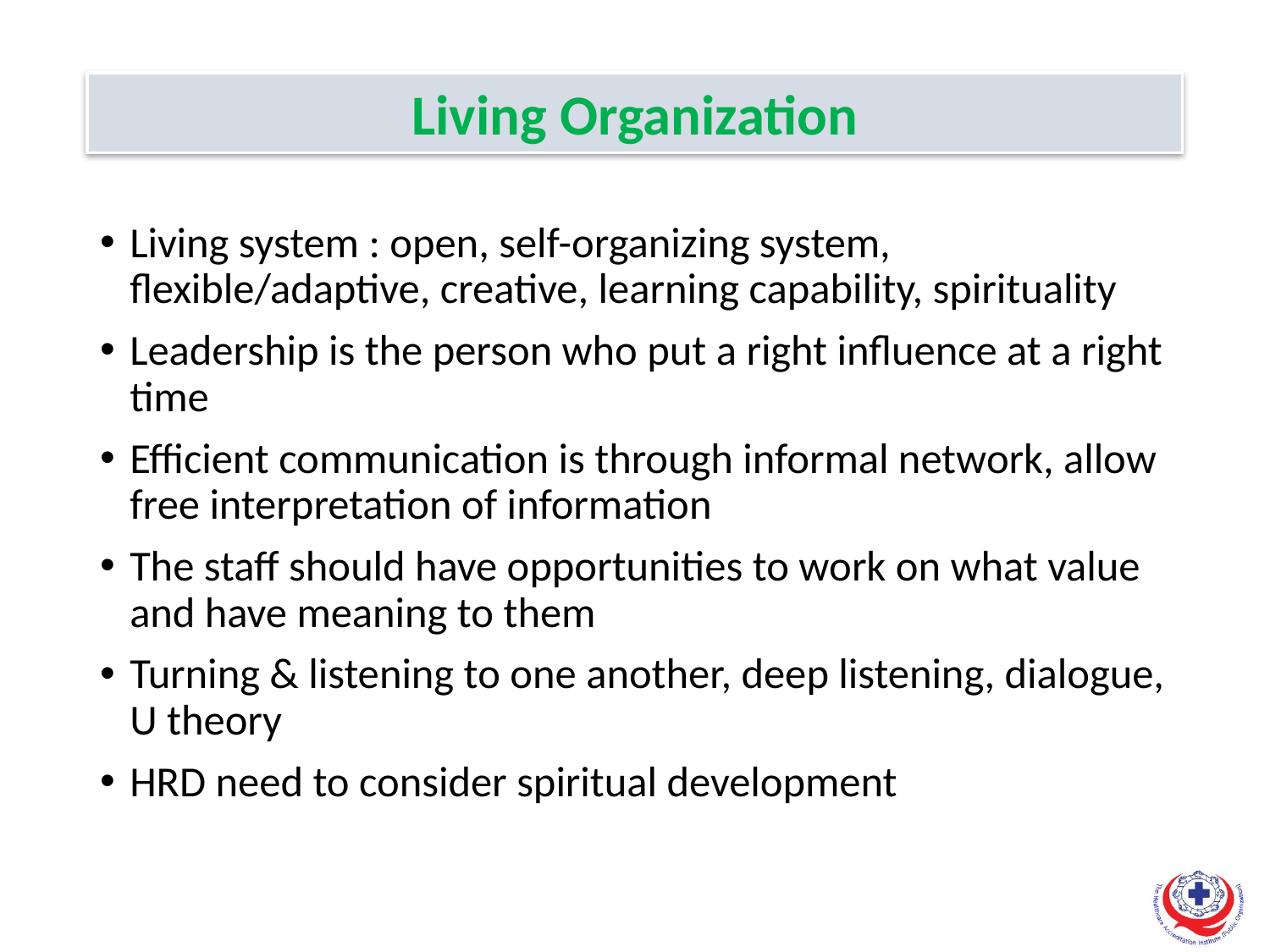

Living Organization
Living system : open, self-organizing system, flexible/adaptive, creative, learning capability, spirituality
Leadership is the person who put a right influence at a right time
Efficient communication is through informal network, allow free interpretation of information
The staff should have opportunities to work on what value and have meaning to them
Turning & listening to one another, deep listening, dialogue, U theory
HRD need to consider spiritual development

## Slide 10
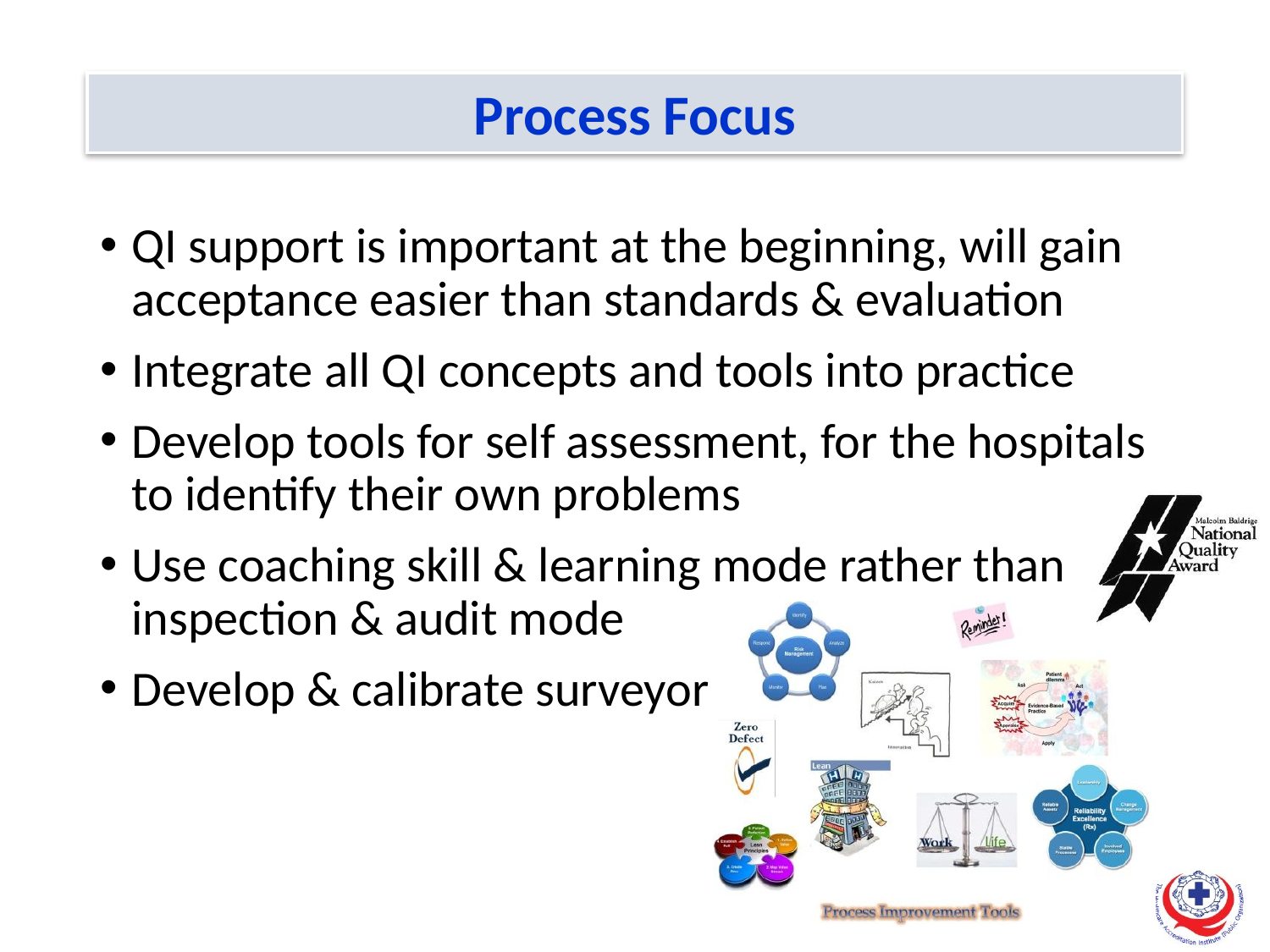

Process Focus
QI support is important at the beginning, will gain acceptance easier than standards & evaluation
Integrate all QI concepts and tools into practice
Develop tools for self assessment, for the hospitals to identify their own problems
Use coaching skill & learning mode rather than inspection & audit mode
Develop & calibrate surveyor

## Slide 11
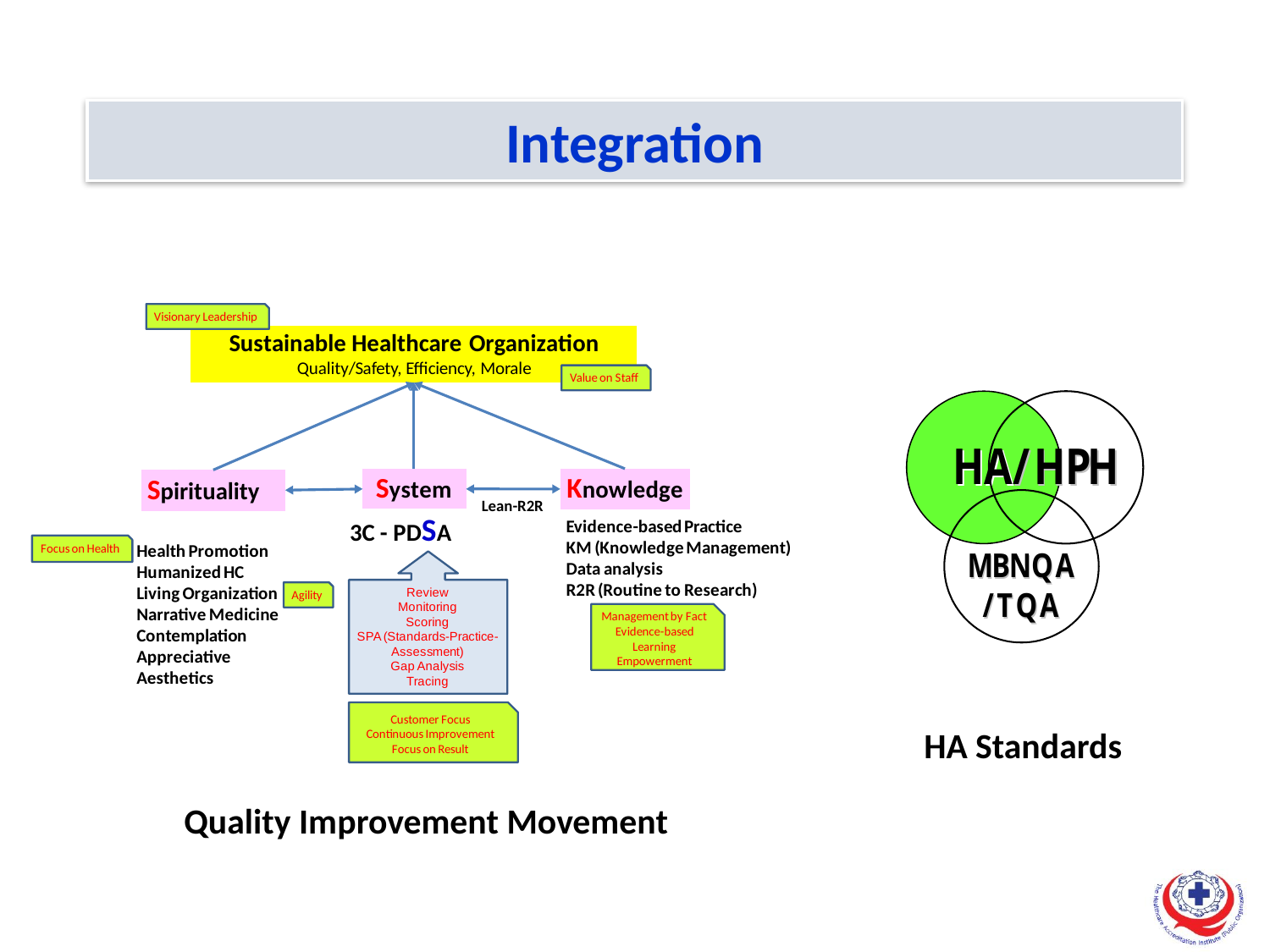

Integration
HA Standards
Quality Improvement Movement

## Slide 12
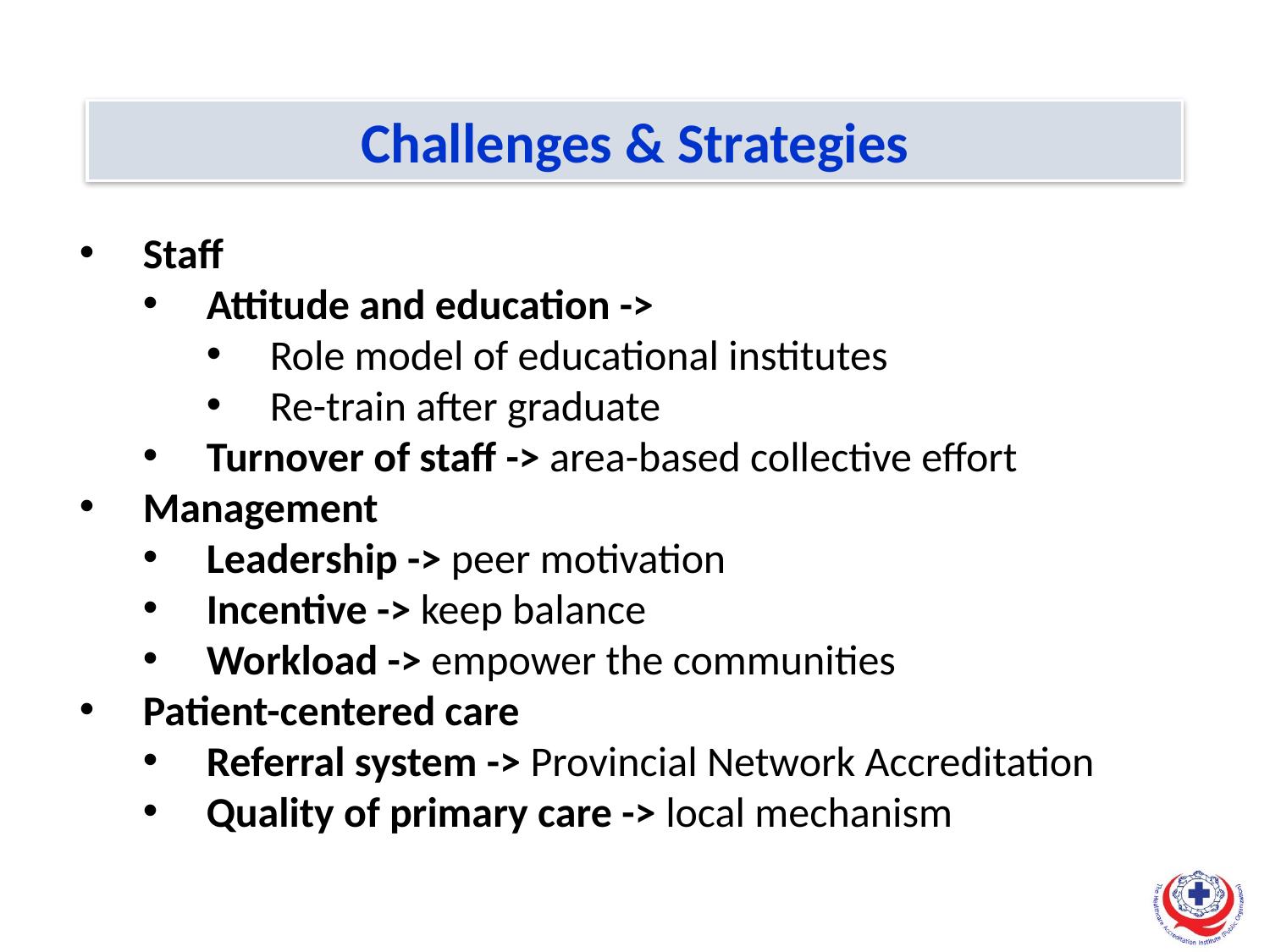

Challenges & Strategies
Staff
Attitude and education ->
Role model of educational institutes
Re-train after graduate
Turnover of staff -> area-based collective effort
Management
Leadership -> peer motivation
Incentive -> keep balance
Workload -> empower the communities
Patient-centered care
Referral system -> Provincial Network Accreditation
Quality of primary care -> local mechanism

## Slide 13
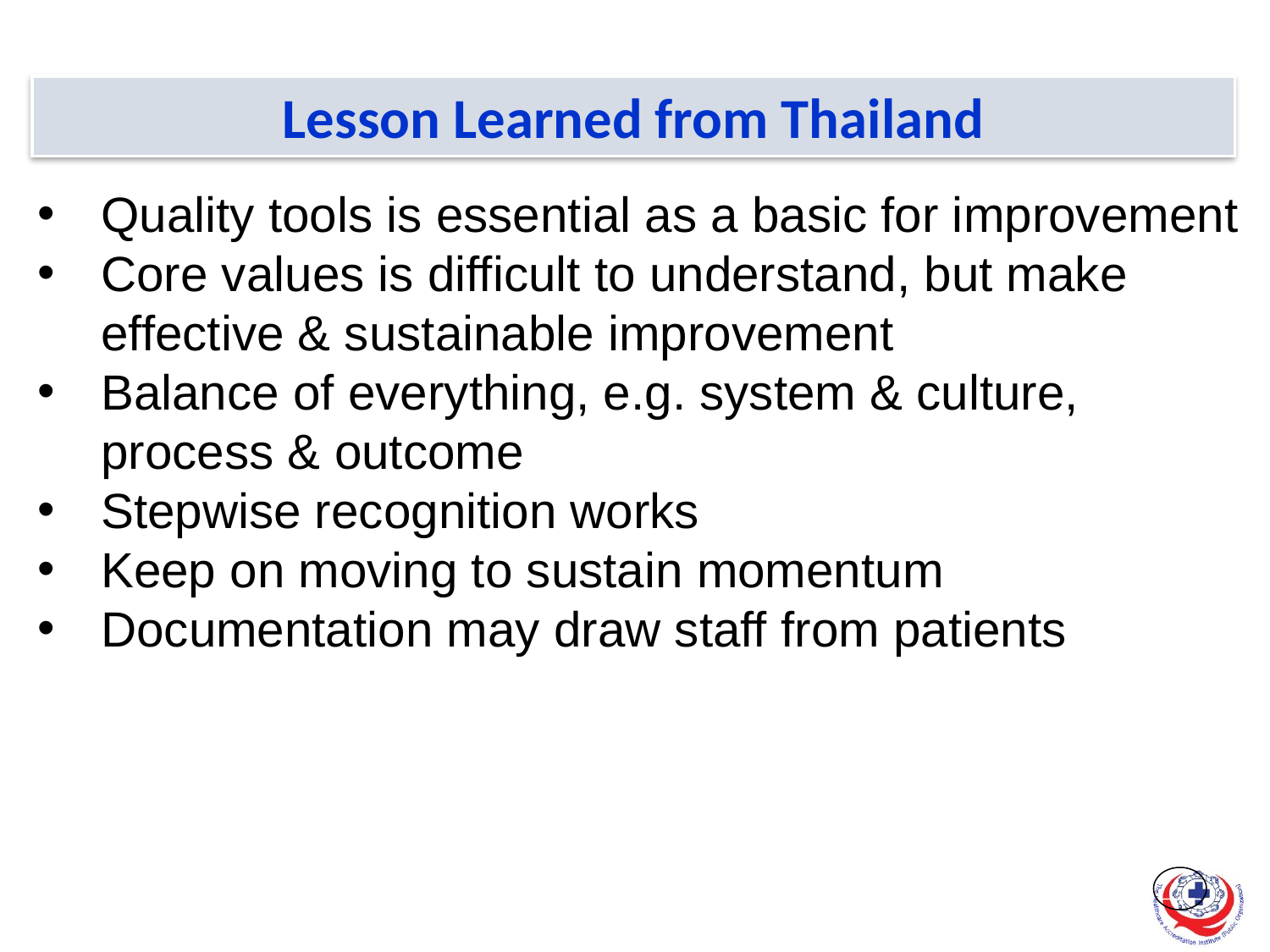

Lesson Learned from Thailand
Quality tools is essential as a basic for improvement
Core values is difficult to understand, but make effective & sustainable improvement
Balance of everything, e.g. system & culture, process & outcome
Stepwise recognition works
Keep on moving to sustain momentum
Documentation may draw staff from patients

## Slide 14
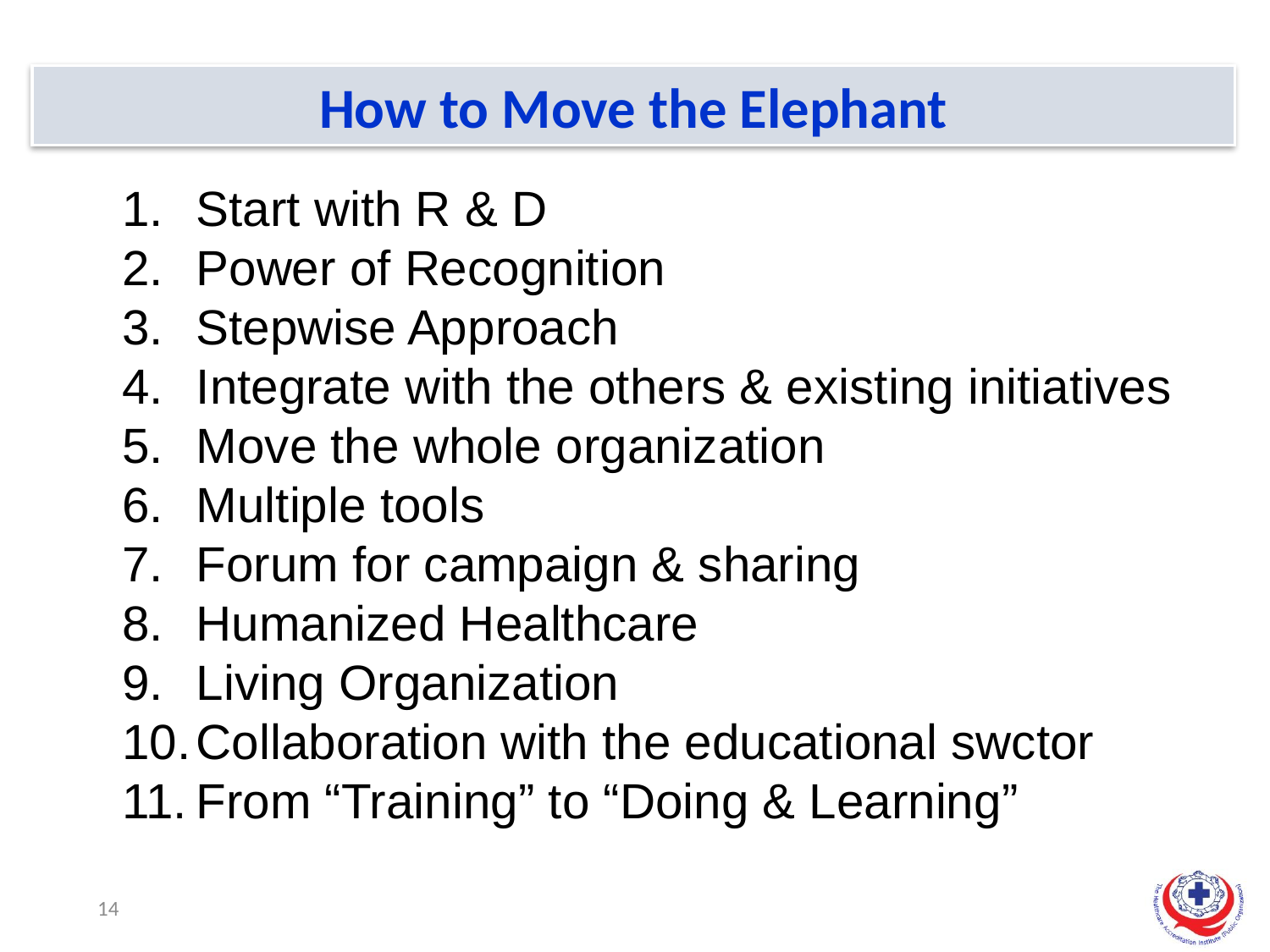

How to Move the Elephant
Start with R & D
Power of Recognition
Stepwise Approach
Integrate with the others & existing initiatives
Move the whole organization
Multiple tools
Forum for campaign & sharing
Humanized Healthcare
Living Organization
Collaboration with the educational swctor
From “Training” to “Doing & Learning”
14

## Slide 15
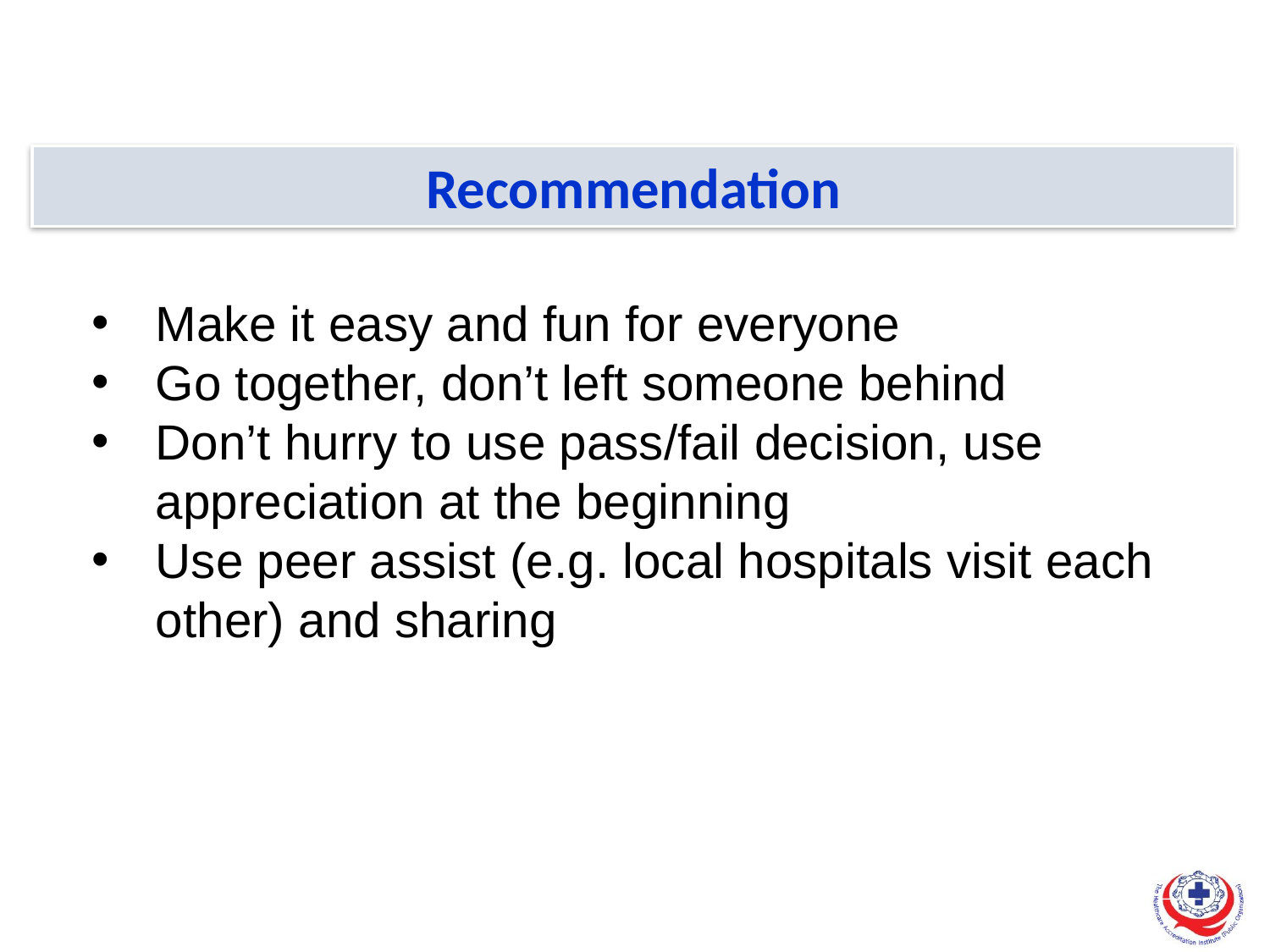

Recommendation
Make it easy and fun for everyone
Go together, don’t left someone behind
Don’t hurry to use pass/fail decision, use appreciation at the beginning
Use peer assist (e.g. local hospitals visit each other) and sharing
