# Supplementary material for: Hospital accreditation: lessons from low- and middle-income countries
Source: Global Health. 2014 Sep 4;10:65. doi: 10.1186/s12992-014-0065-9 (PMC4159532; doi:10.1186/s12992-014-0065-9)
Supplement: Additional file 2: — Malaysian Health Sector. [file 12992_2014_65_MOESM2_ESM.pptx]

## Slide 1
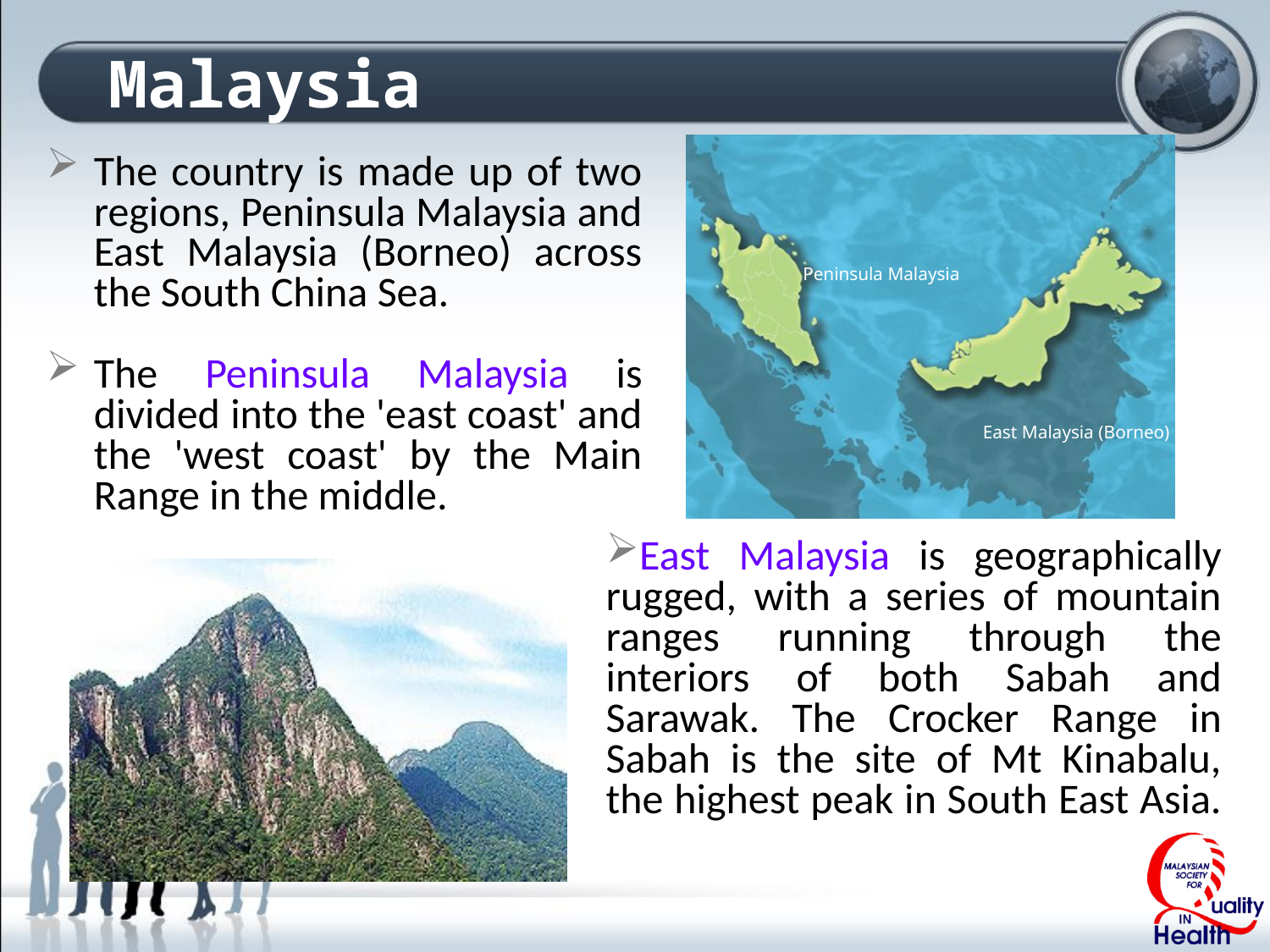

# Malaysia
The country is made up of two regions, Peninsula Malaysia and East Malaysia (Borneo) across the South China Sea.
The Peninsula Malaysia is divided into the 'east coast' and the 'west coast' by the Main Range in the middle.
Peninsula Malaysia
East Malaysia (Borneo)
East Malaysia is geographically rugged, with a series of mountain ranges running through the interiors of both Sabah and Sarawak. The Crocker Range in Sabah is the site of Mt Kinabalu, the highest peak in South East Asia.

## Slide 2
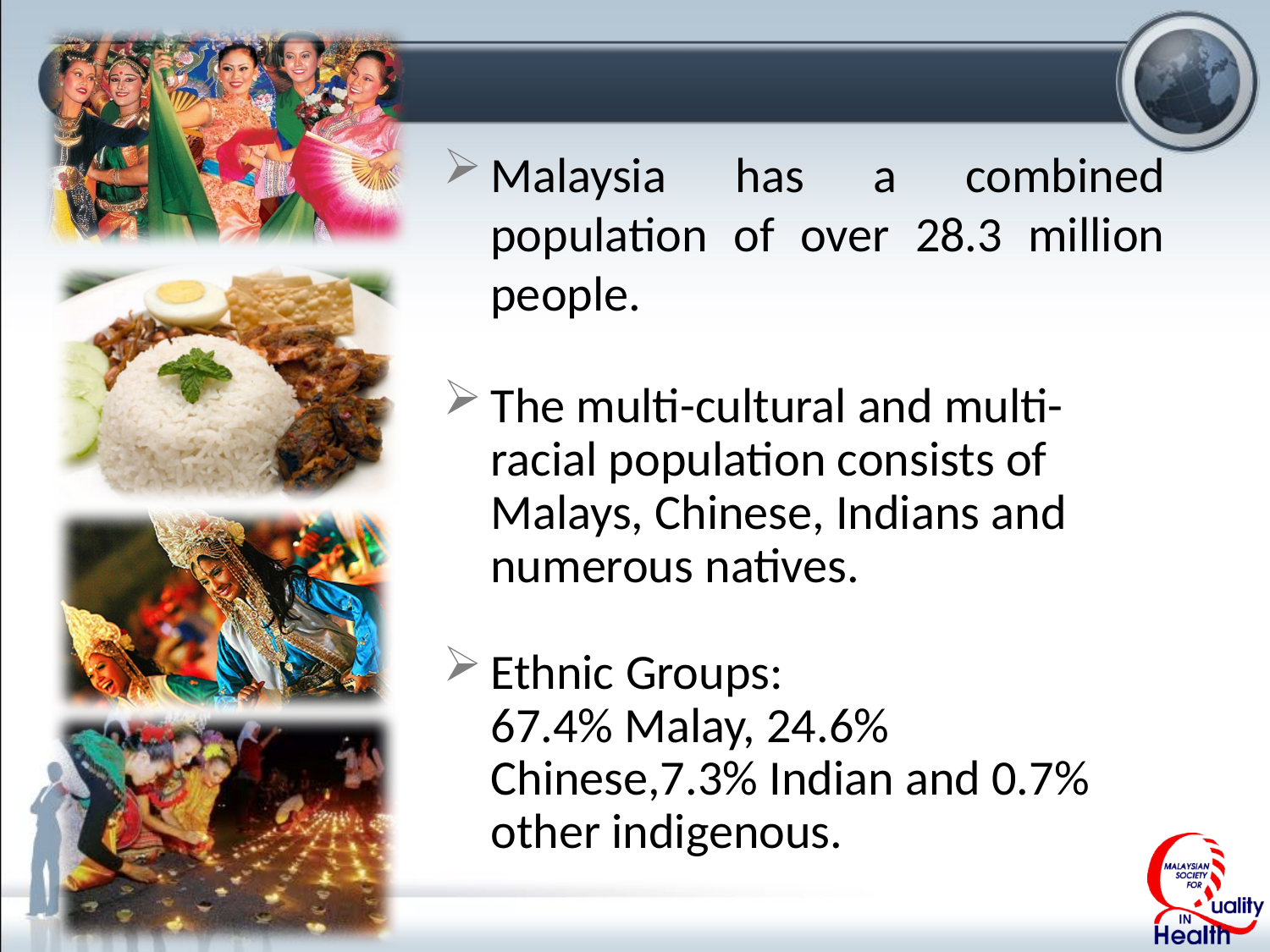

Malaysia has a combined population of over 28.3 million people.
The multi-cultural and multi-racial population consists of Malays, Chinese, Indians and numerous natives.
Ethnic Groups:
67.4% Malay, 24.6% Chinese,7.3% Indian and 0.7% other indigenous.

## Slide 3
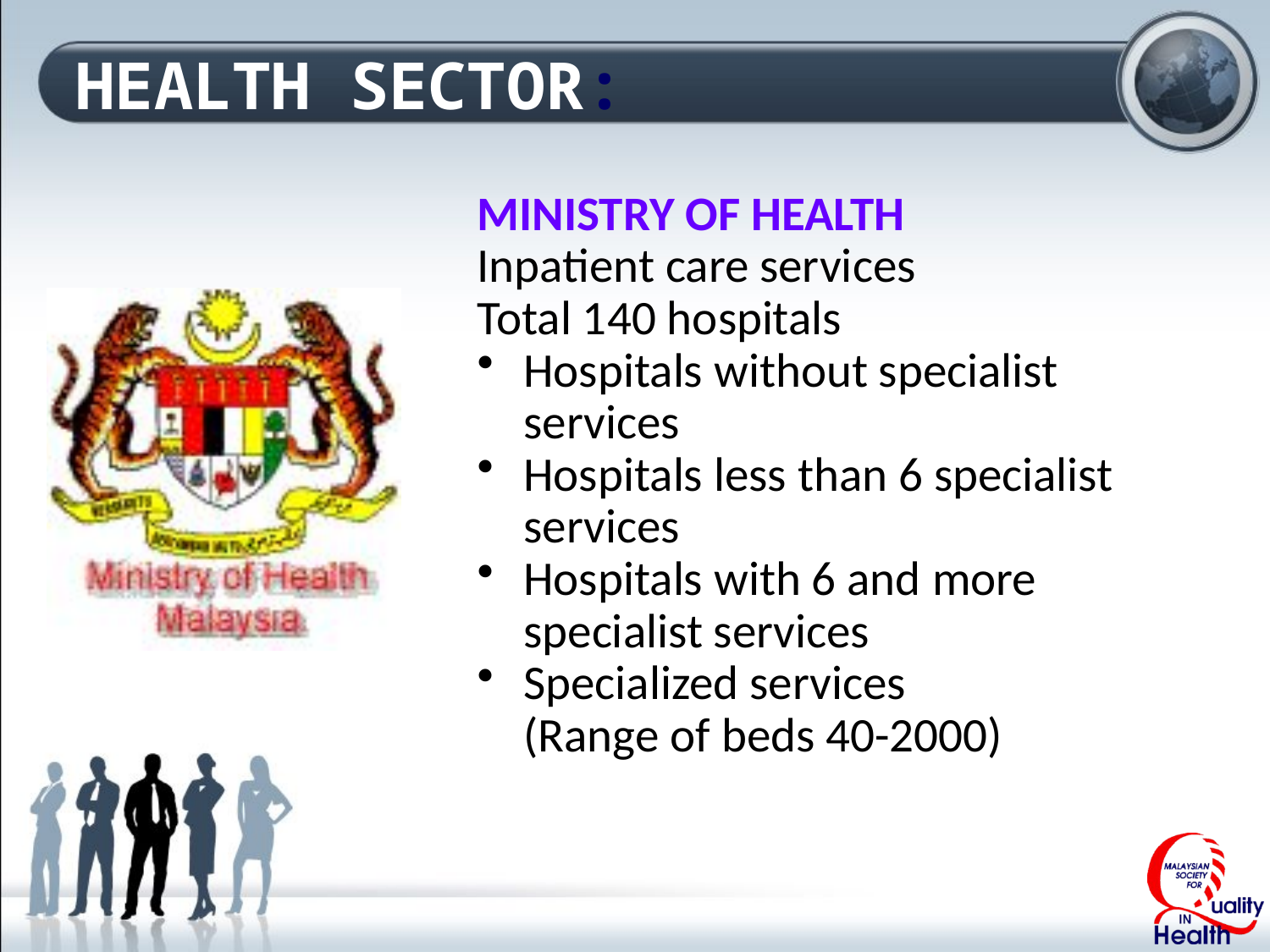

# HEALTH SECTOR:
MINISTRY OF HEALTH
Inpatient care services
Total 140 hospitals
Hospitals without specialist services
Hospitals less than 6 specialist services
Hospitals with 6 and more specialist services
Specialized services
	(Range of beds 40-2000)

## Slide 4
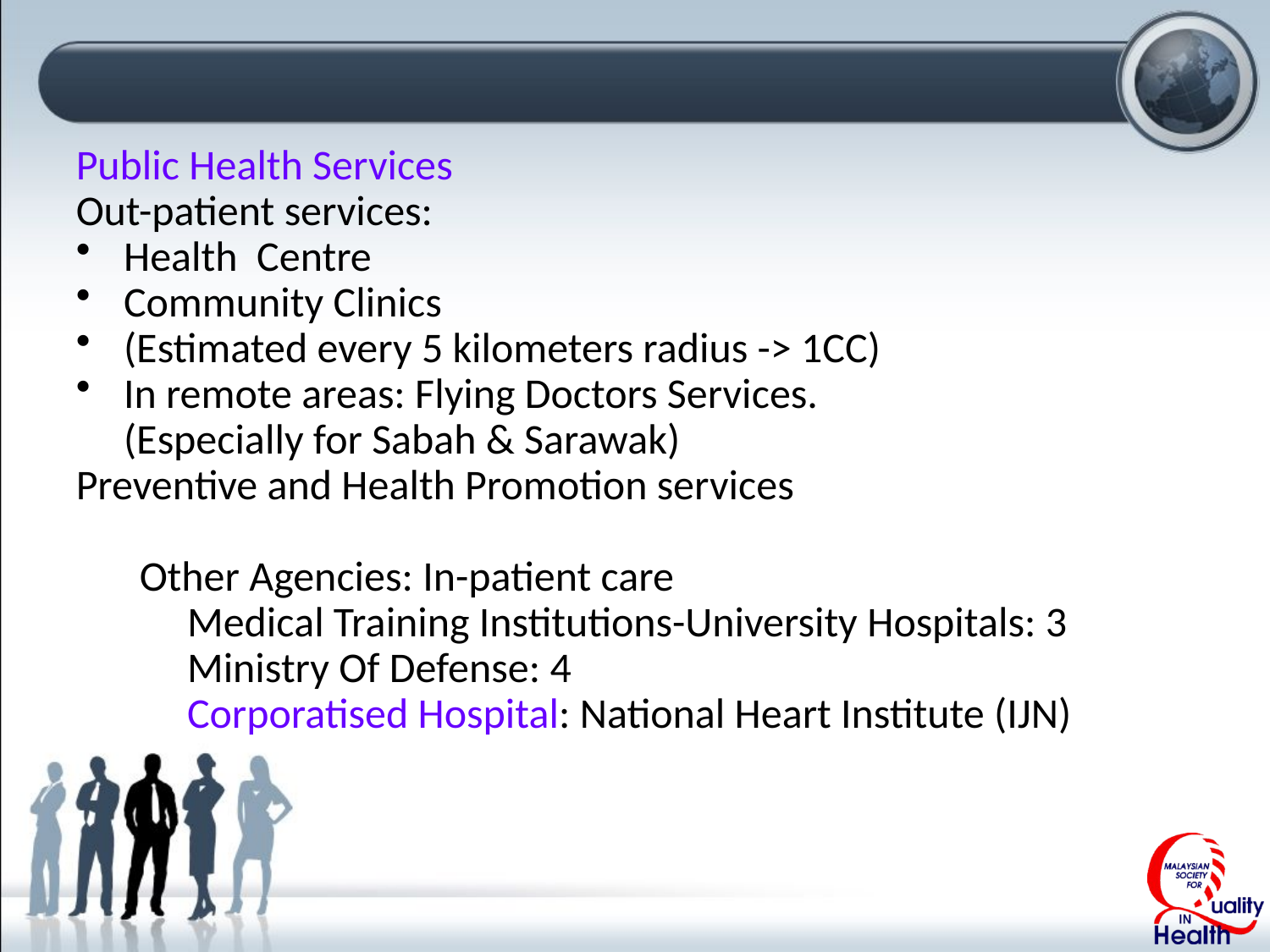

Public Health Services
Out-patient services:
Health Centre
Community Clinics
(Estimated every 5 kilometers radius -> 1CC)
In remote areas: Flying Doctors Services.
	(Especially for Sabah & Sarawak)
Preventive and Health Promotion services
Other Agencies: In-patient care
	Medical Training Institutions-University Hospitals: 3
	Ministry Of Defense: 4
 Corporatised Hospital: National Heart Institute (IJN)

## Slide 5
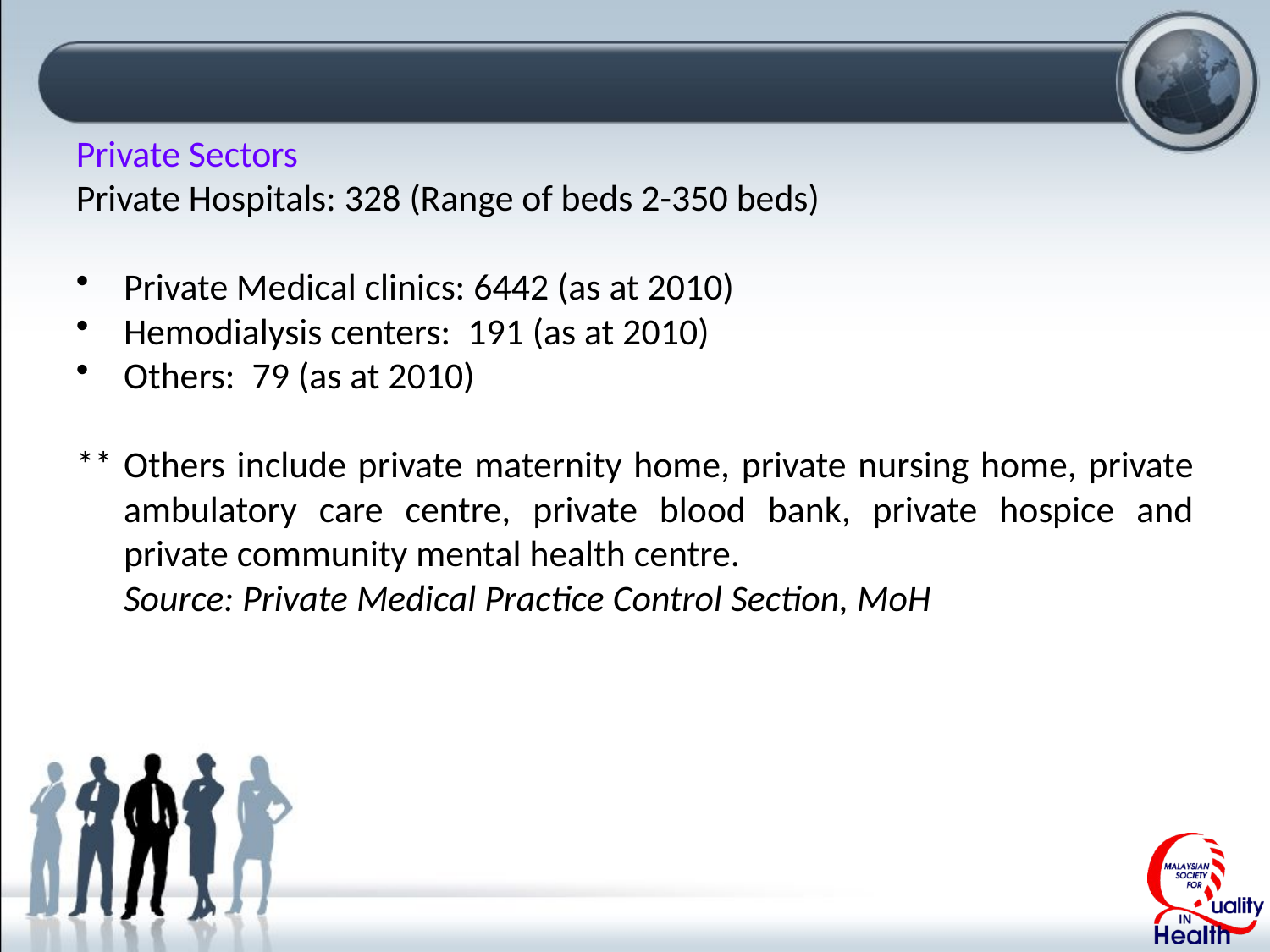

Private Sectors
Private Hospitals: 328 (Range of beds 2-350 beds)
Private Medical clinics: 6442 (as at 2010)
Hemodialysis centers: 191 (as at 2010)
Others: 79 (as at 2010)
**	Others include private maternity home, private nursing home, private ambulatory care centre, private blood bank, private hospice and private community mental health centre.
	Source: Private Medical Practice Control Section, MoH

## Slide 6
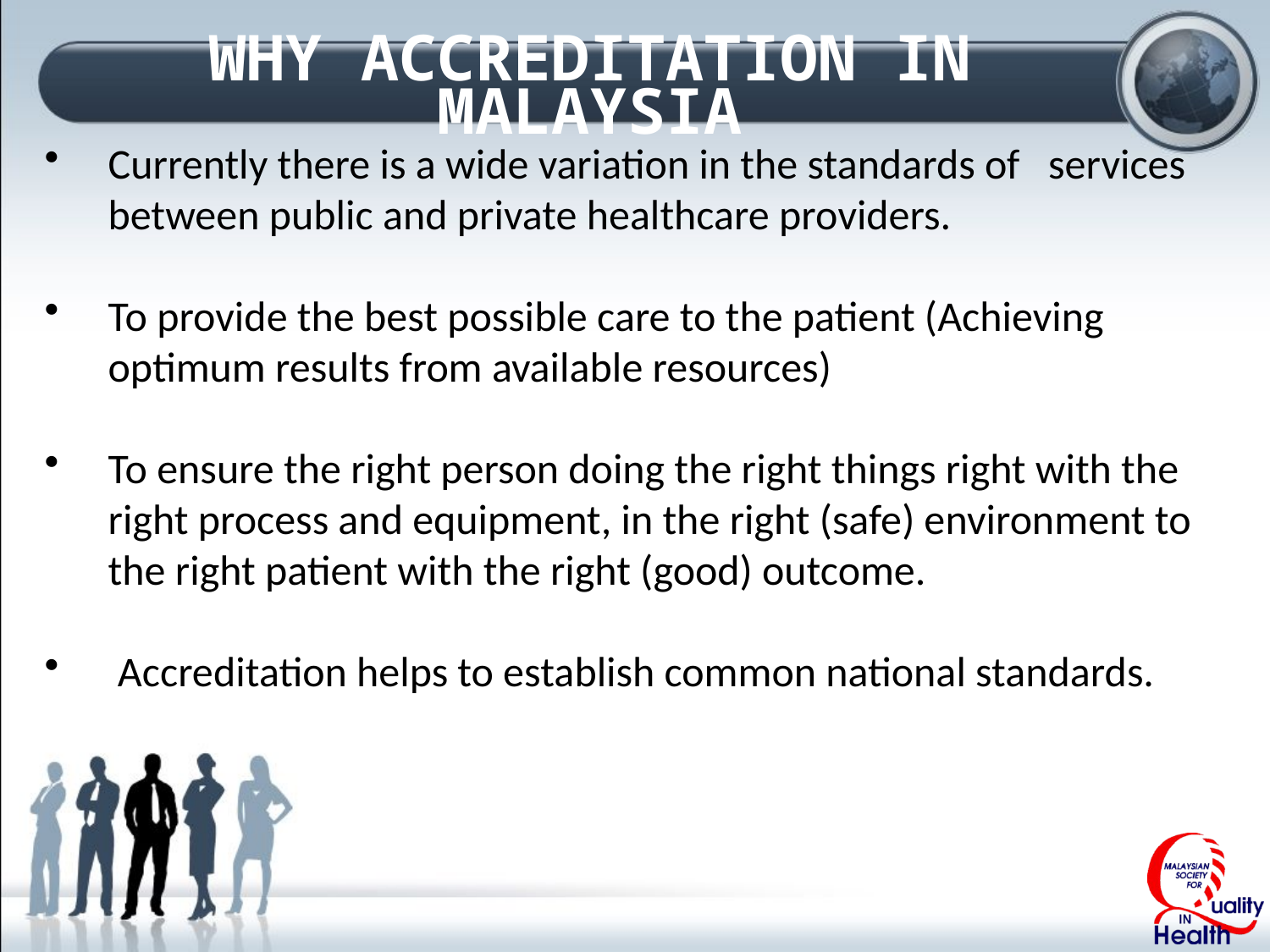

WHY ACCREDITATION IN MALAYSIA
Currently there is a wide variation in the standards of services between public and private healthcare providers.
To provide the best possible care to the patient (Achieving optimum results from available resources)
To ensure the right person doing the right things right with the right process and equipment, in the right (safe) environment to the right patient with the right (good) outcome.
 Accreditation helps to establish common national standards.

## Slide 7
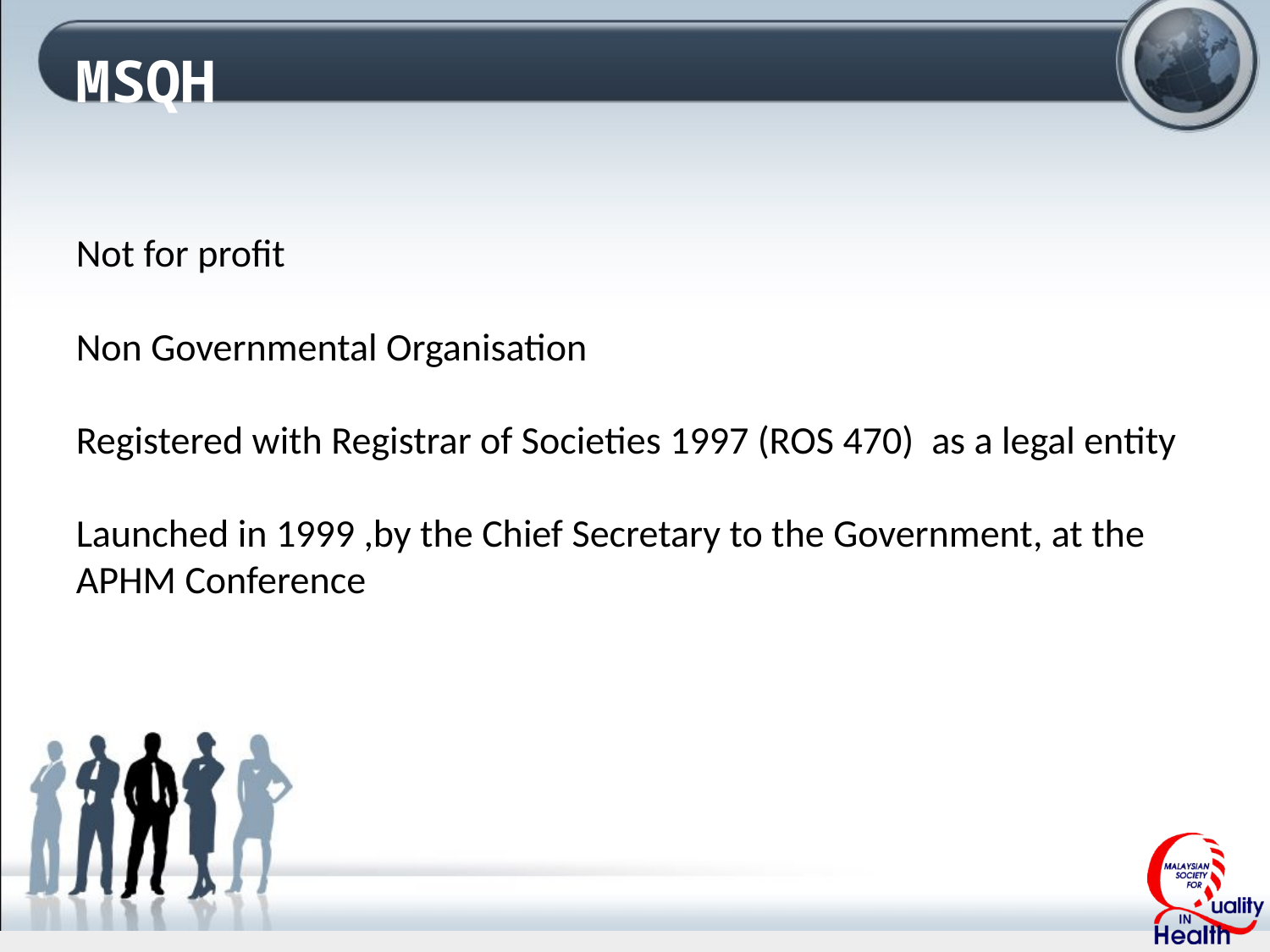

# MSQH
Not for profit
Non Governmental Organisation
Registered with Registrar of Societies 1997 (ROS 470) as a legal entity
Launched in 1999 ,by the Chief Secretary to the Government, at the APHM Conference

## Slide 8
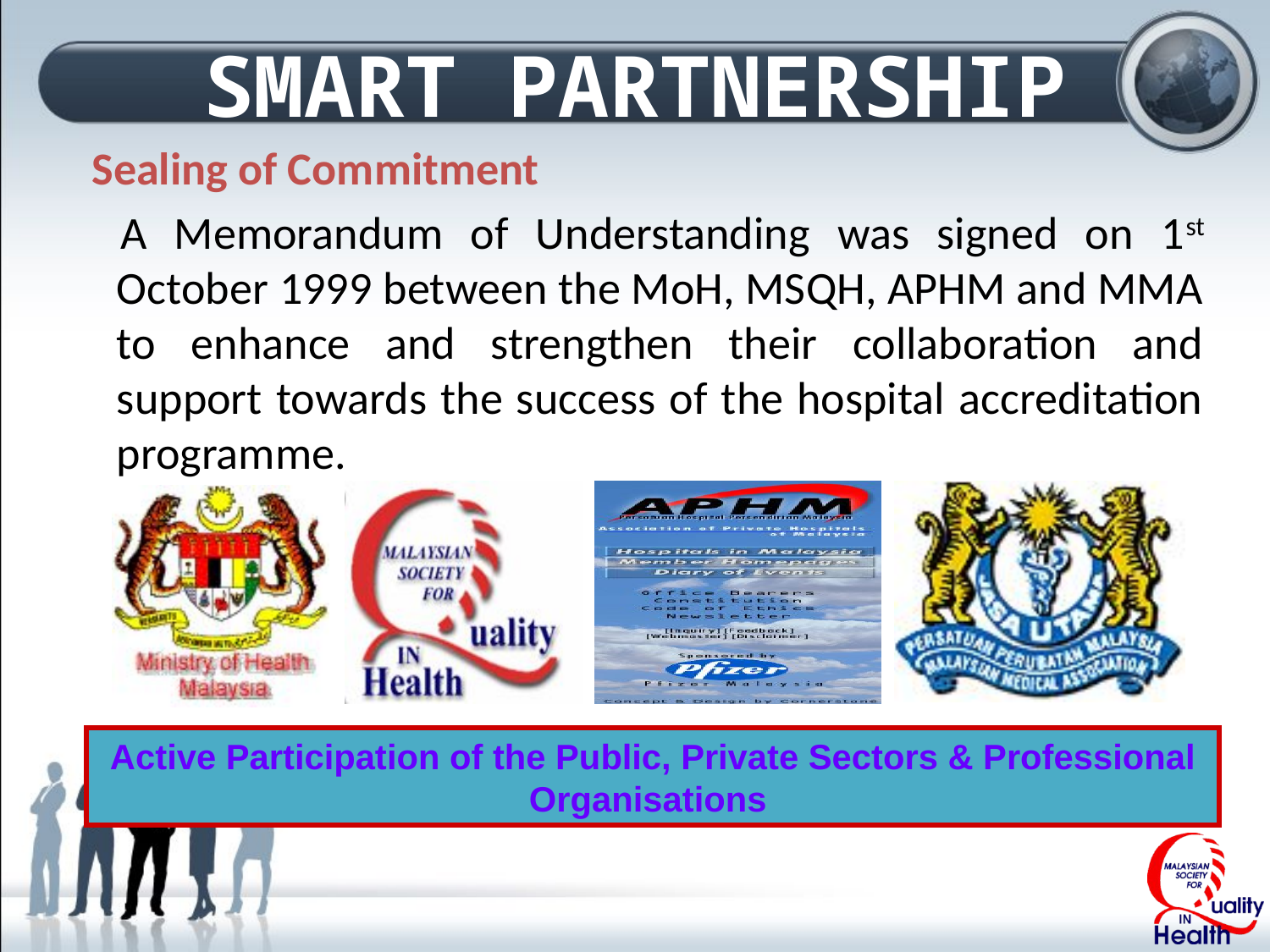

SMART PARTNERSHIP
	Sealing of Commitment
A Memorandum of Understanding was signed on 1st October 1999 between the MoH, MSQH, APHM and MMA to enhance and strengthen their collaboration and support towards the success of the hospital accreditation programme.
Active Participation of the Public, Private Sectors & Professional Organisations

## Slide 9
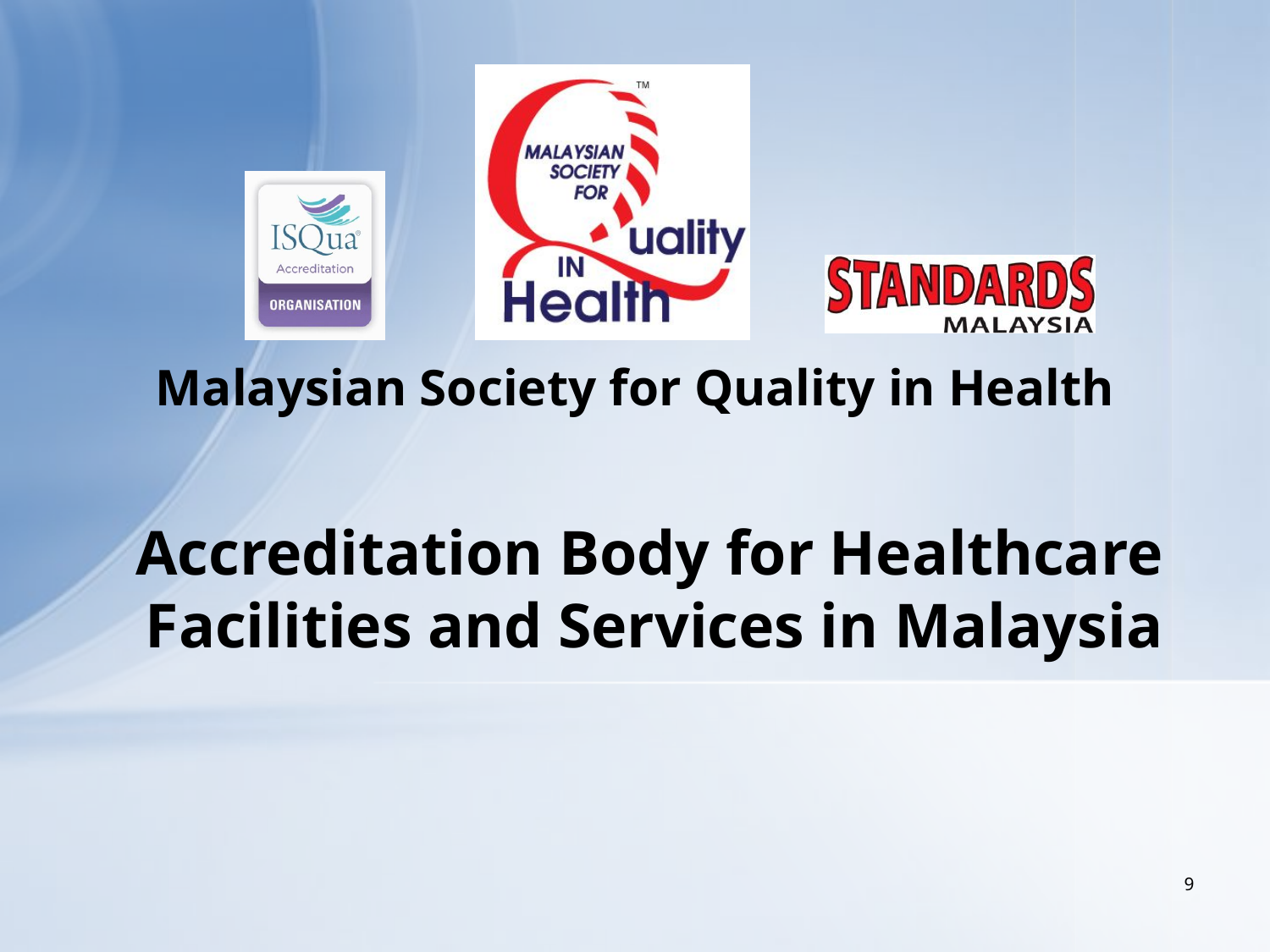

Malaysian Society for Quality in Health
# Accreditation Body for Healthcare Facilities and Services in Malaysia
9

## Slide 10
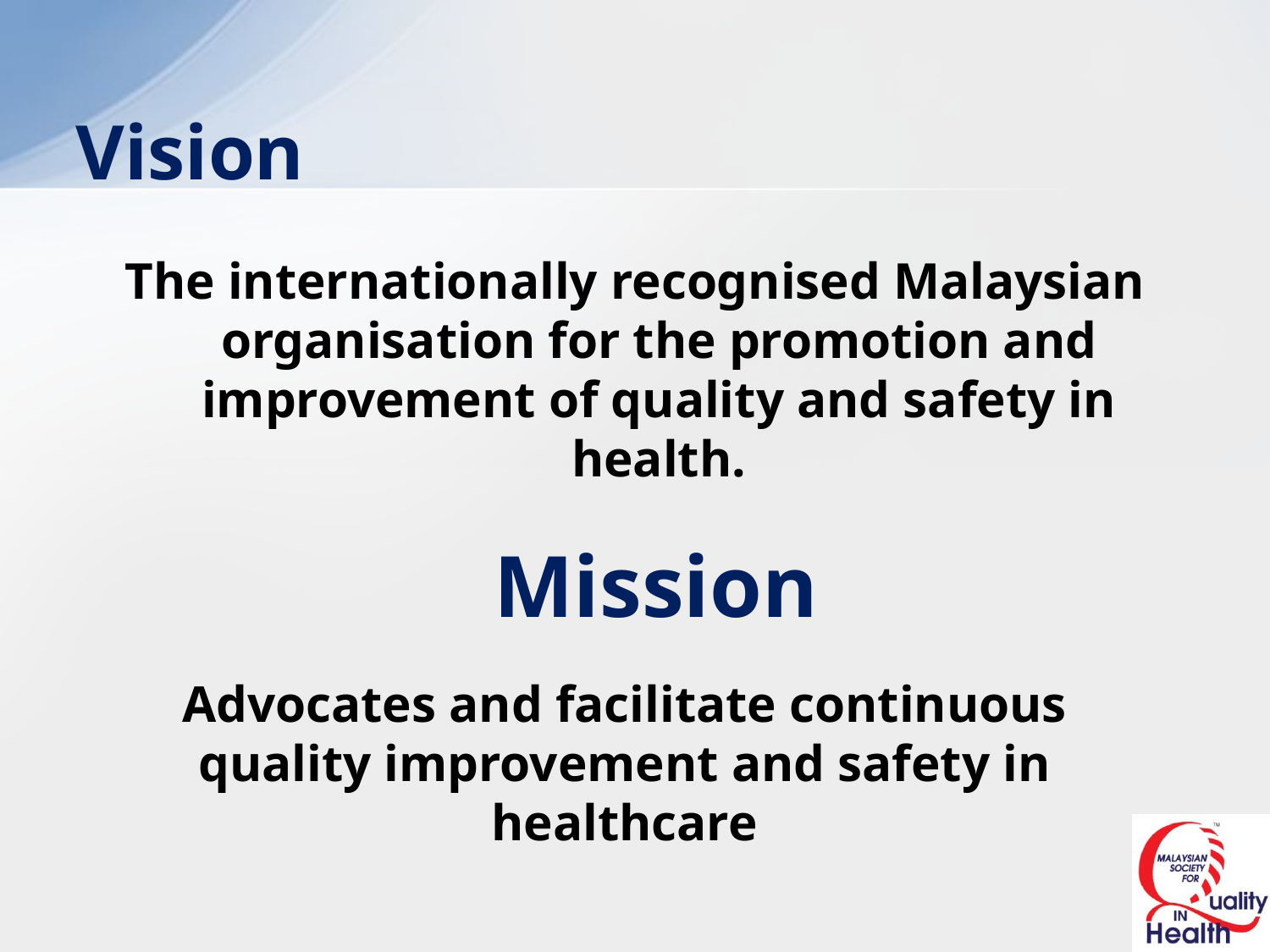

# Vision
The internationally recognised Malaysian organisation for the promotion and improvement of quality and safety in health.
Mission
Advocates and facilitate continuous quality improvement and safety in healthcare

## Slide 11
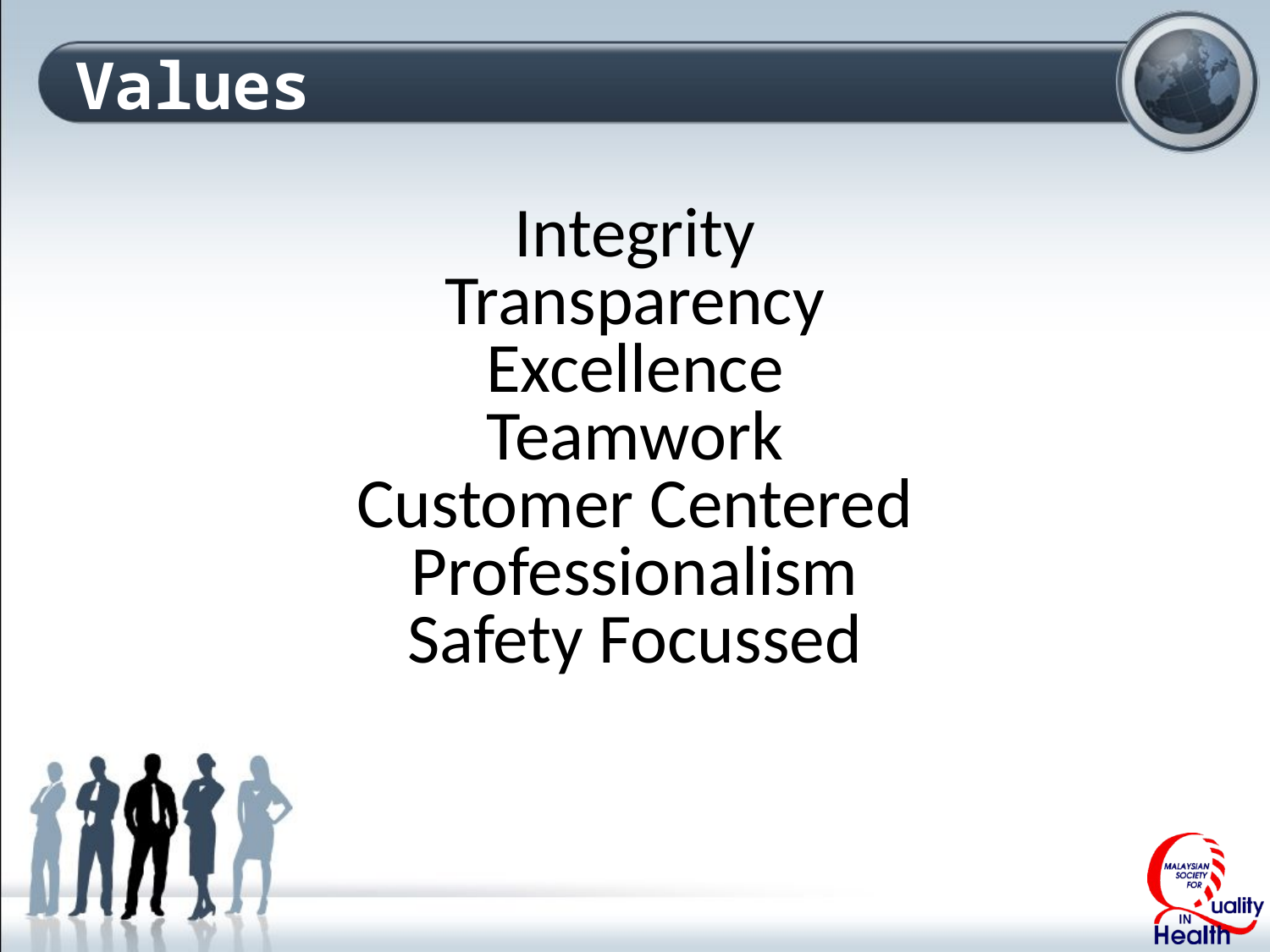

# Values
Integrity
Transparency
Excellence
Teamwork
Customer Centered
Professionalism
Safety Focussed

## Slide 12
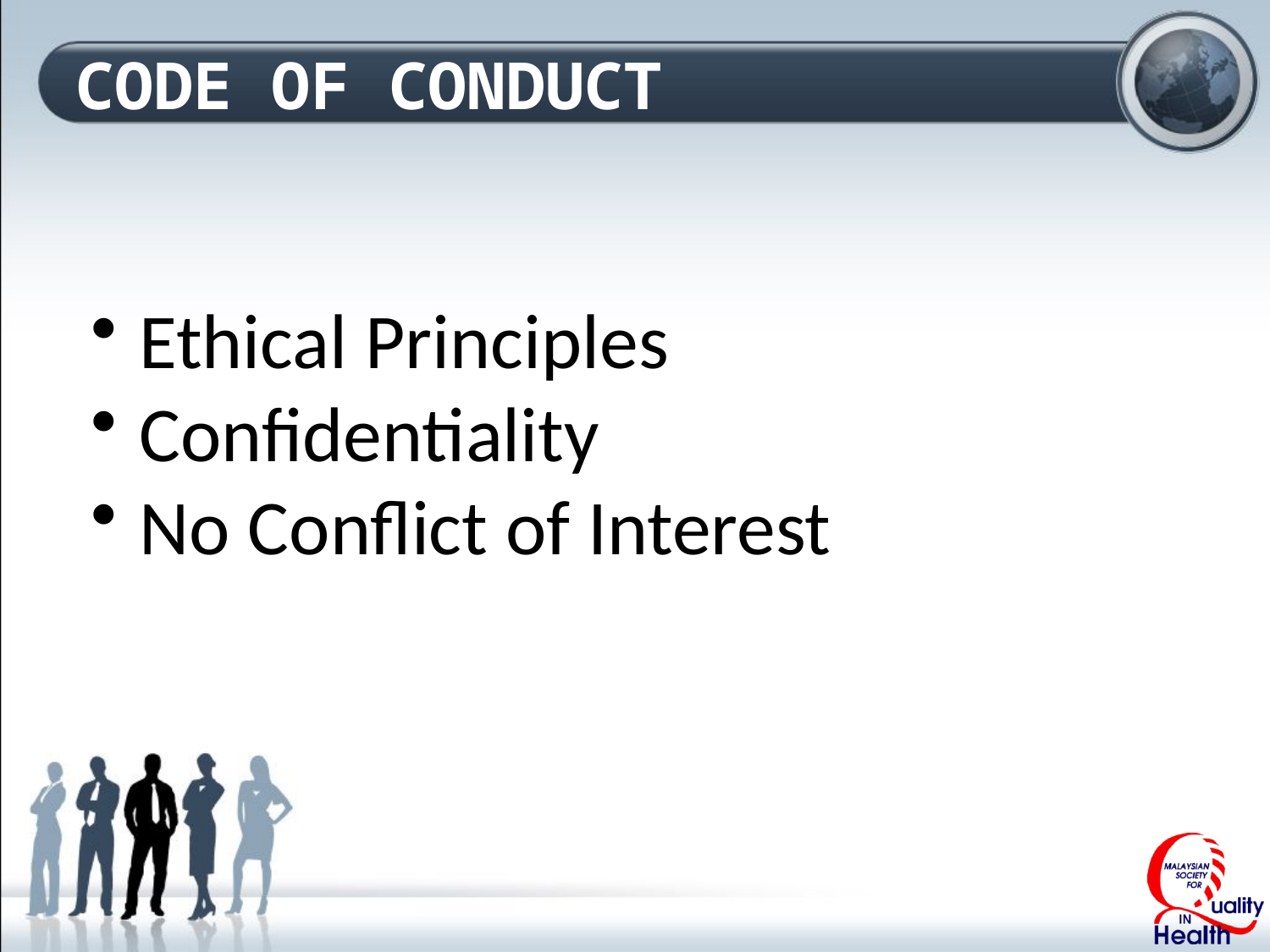

# CODE OF CONDUCT
Ethical Principles
Confidentiality
No Conflict of Interest

## Slide 13
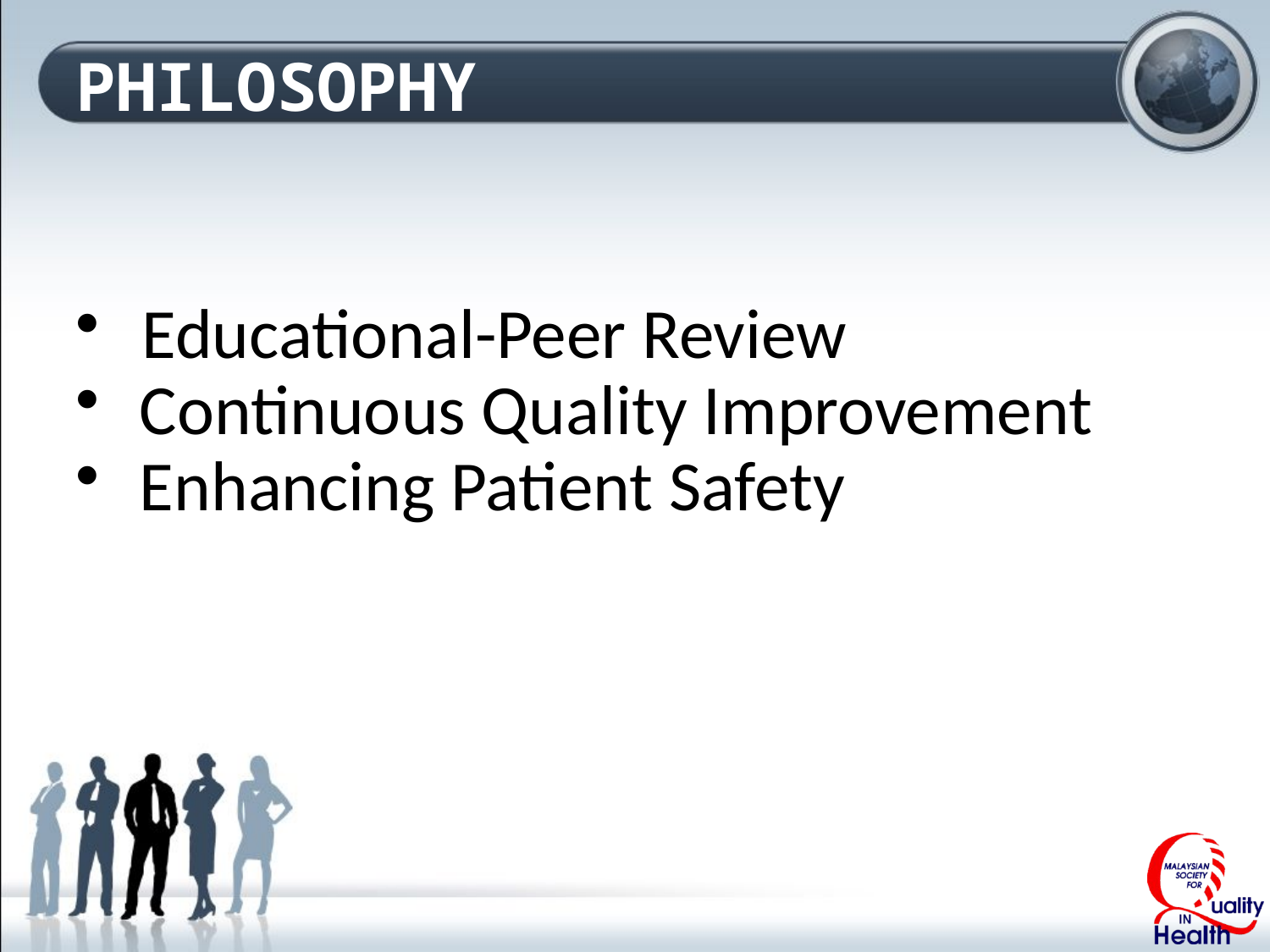

# PHILOSOPHY
 Educational-Peer Review
 Continuous Quality Improvement
 Enhancing Patient Safety

## Slide 14
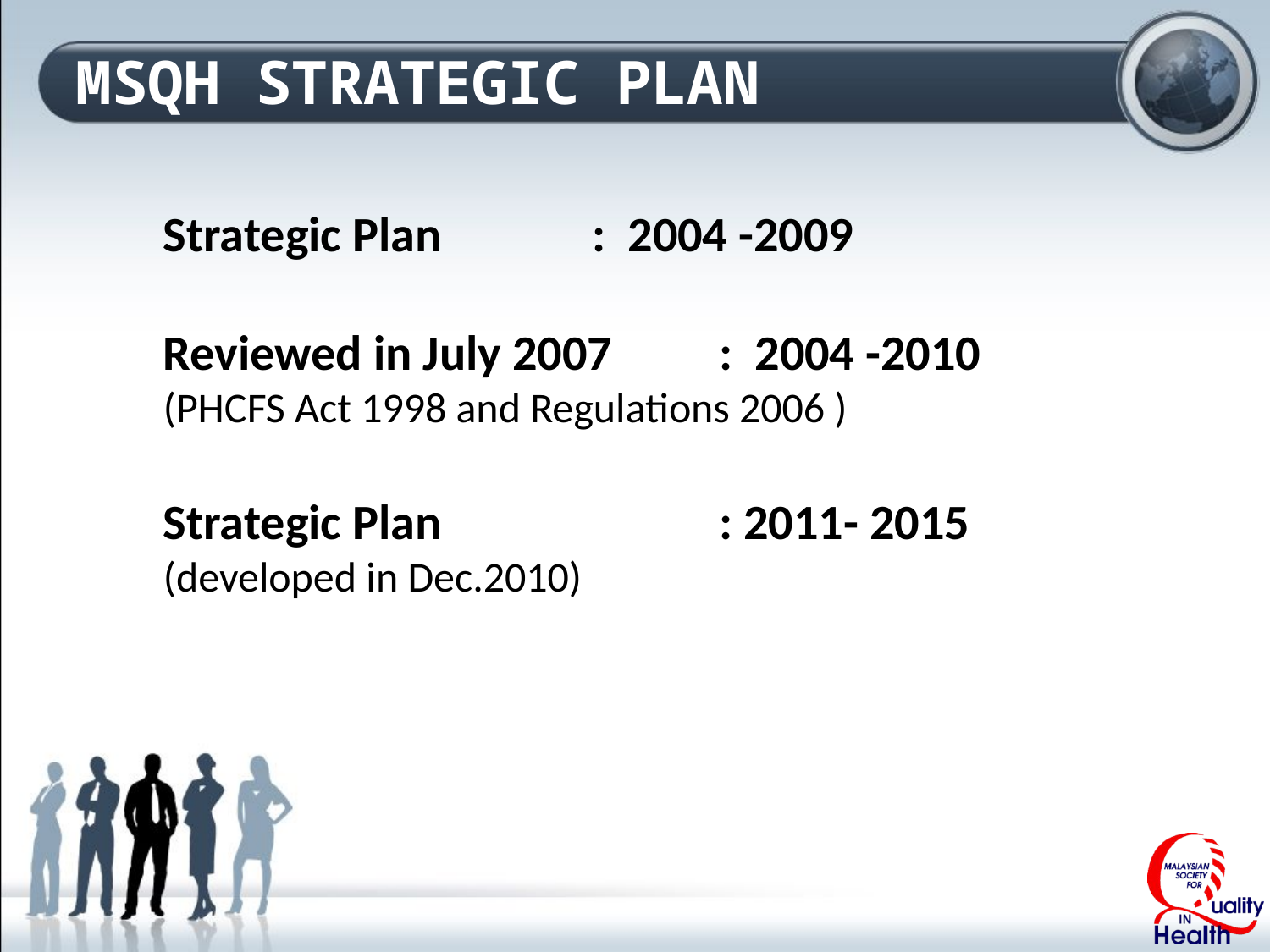

# MSQH STRATEGIC PLAN
Strategic Plan		: 2004 -2009
Reviewed in July 2007	: 2004 -2010
(PHCFS Act 1998 and Regulations 2006 )
Strategic Plan 		: 2011- 2015
(developed in Dec.2010)

## Slide 15
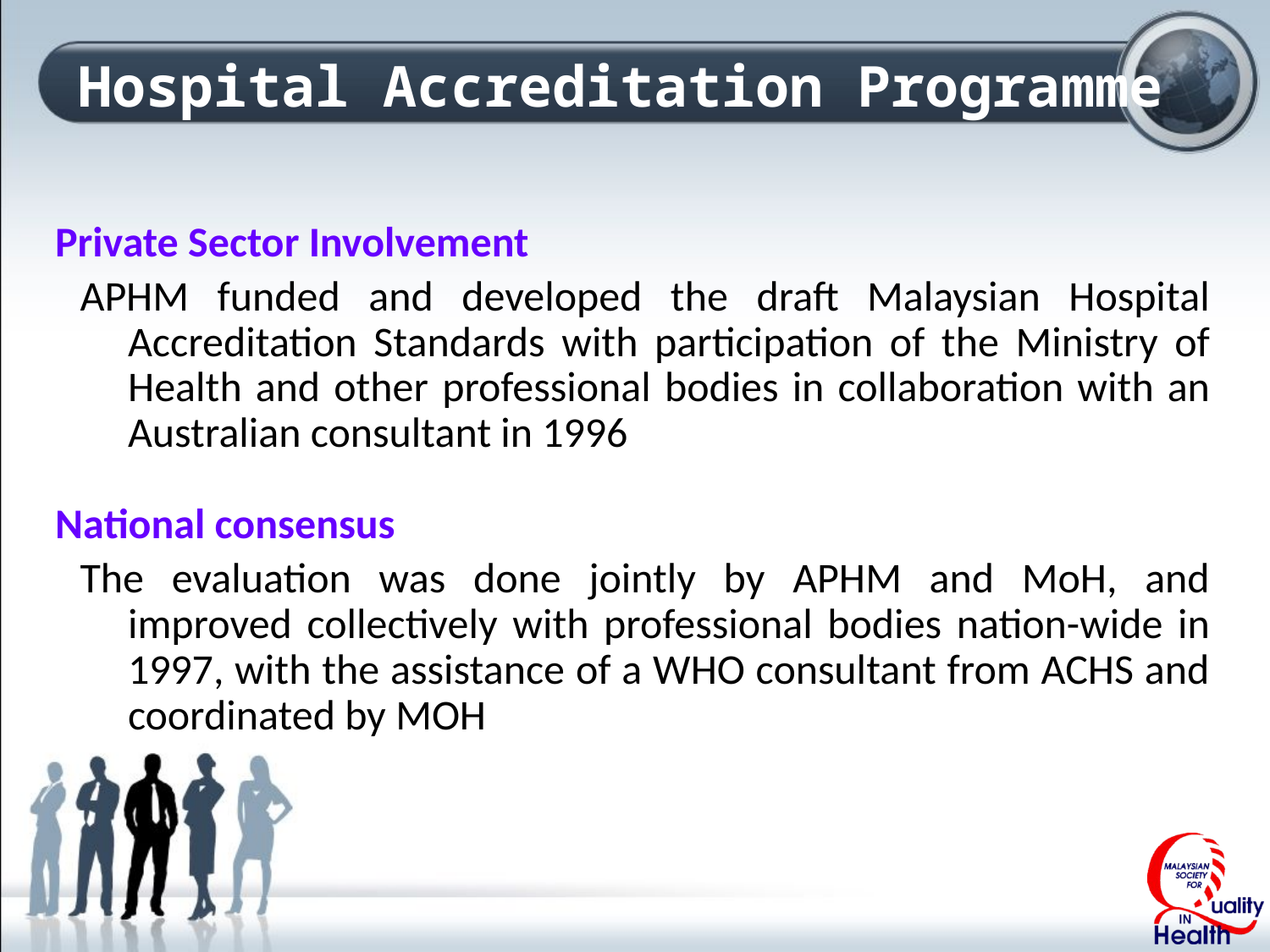

Hospital Accreditation Programme
Private Sector Involvement
APHM funded and developed the draft Malaysian Hospital Accreditation Standards with participation of the Ministry of Health and other professional bodies in collaboration with an Australian consultant in 1996
National consensus
The evaluation was done jointly by APHM and MoH, and improved collectively with professional bodies nation-wide in 1997, with the assistance of a WHO consultant from ACHS and coordinated by MOH

## Slide 16
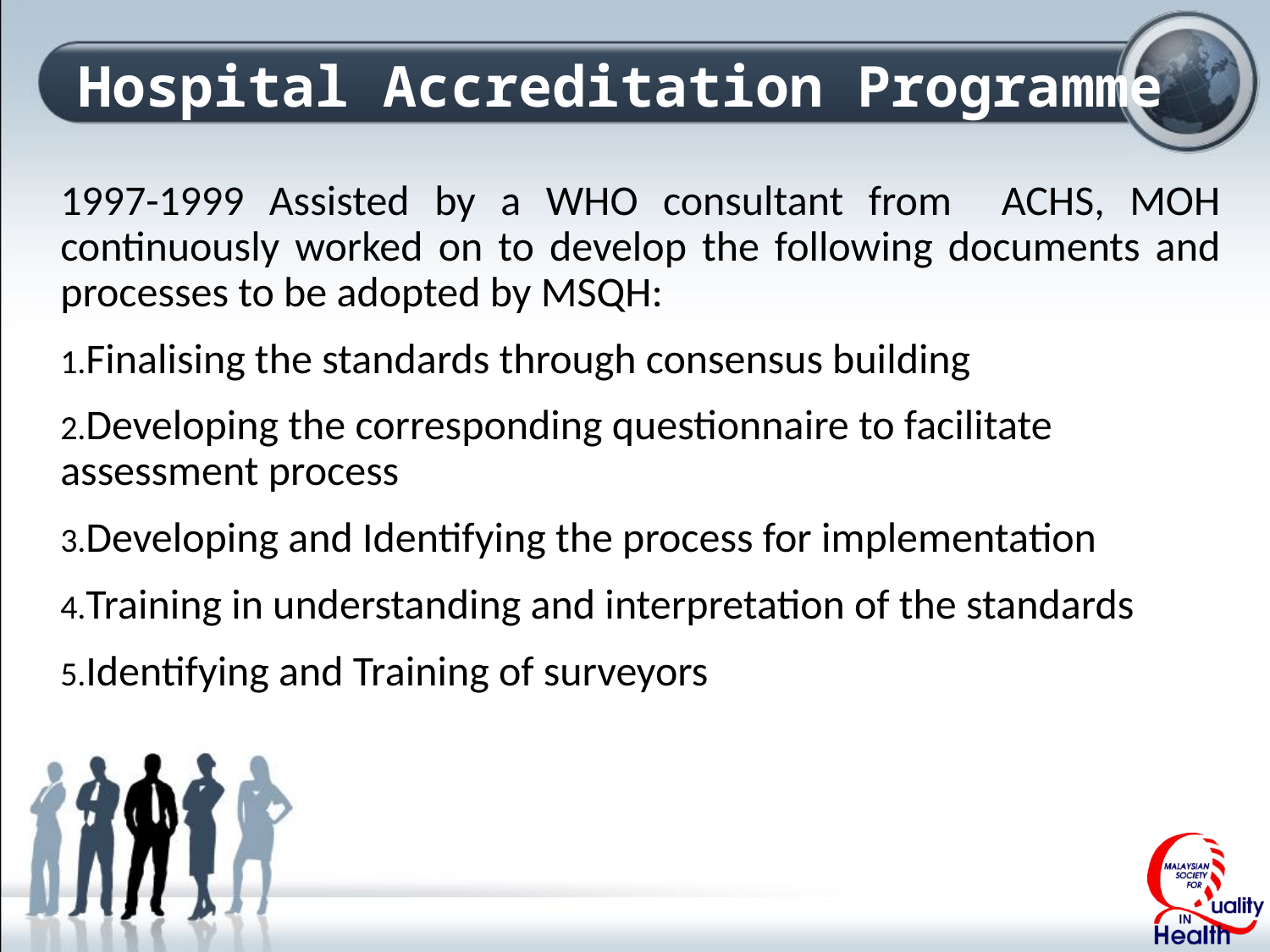

Hospital Accreditation Programme
1997-1999 Assisted by a WHO consultant from ACHS, MOH continuously worked on to develop the following documents and processes to be adopted by MSQH:
Finalising the standards through consensus building
Developing the corresponding questionnaire to facilitate assessment process
Developing and Identifying the process for implementation
Training in understanding and interpretation of the standards
Identifying and Training of surveyors

## Slide 17
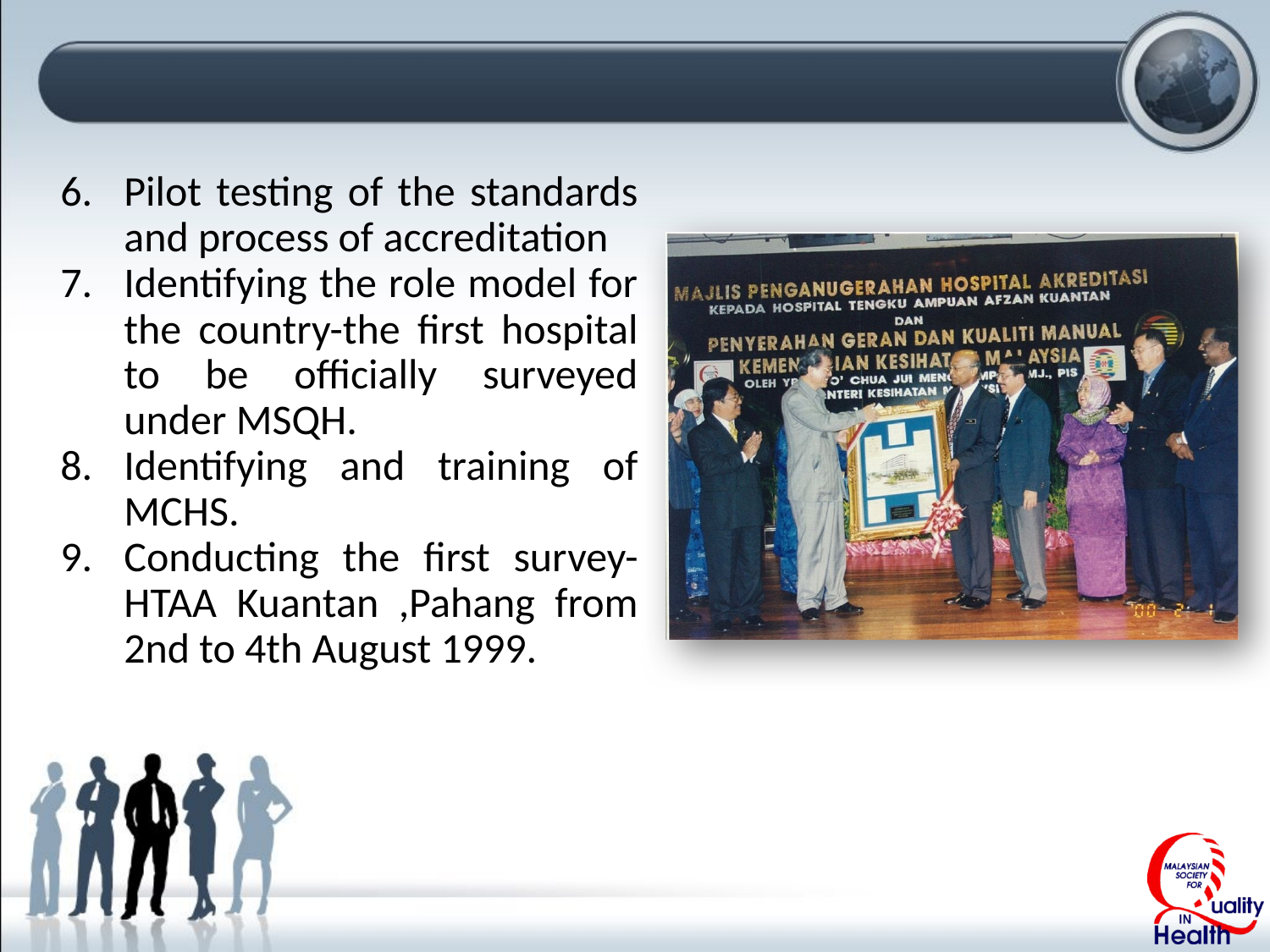

Pilot testing of the standards and process of accreditation
Identifying the role model for the country-the first hospital to be officially surveyed under MSQH.
Identifying and training of MCHS.
Conducting the first survey-HTAA Kuantan ,Pahang from 2nd to 4th August 1999.

## Slide 18
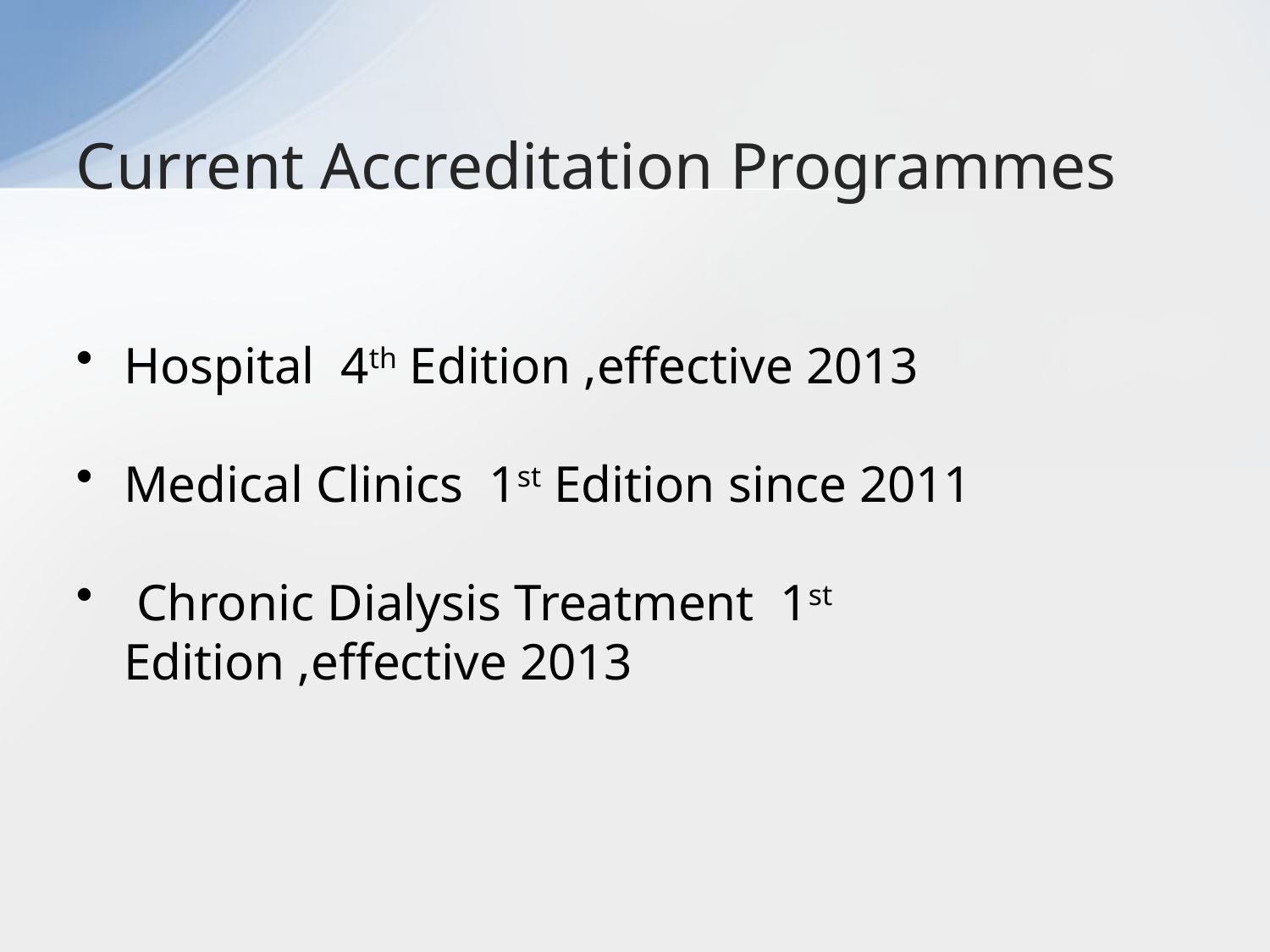

# Current Accreditation Programmes
Hospital 4th Edition ,effective 2013
Medical Clinics 1st Edition since 2011
 Chronic Dialysis Treatment 1st Edition ,effective 2013

## Slide 19
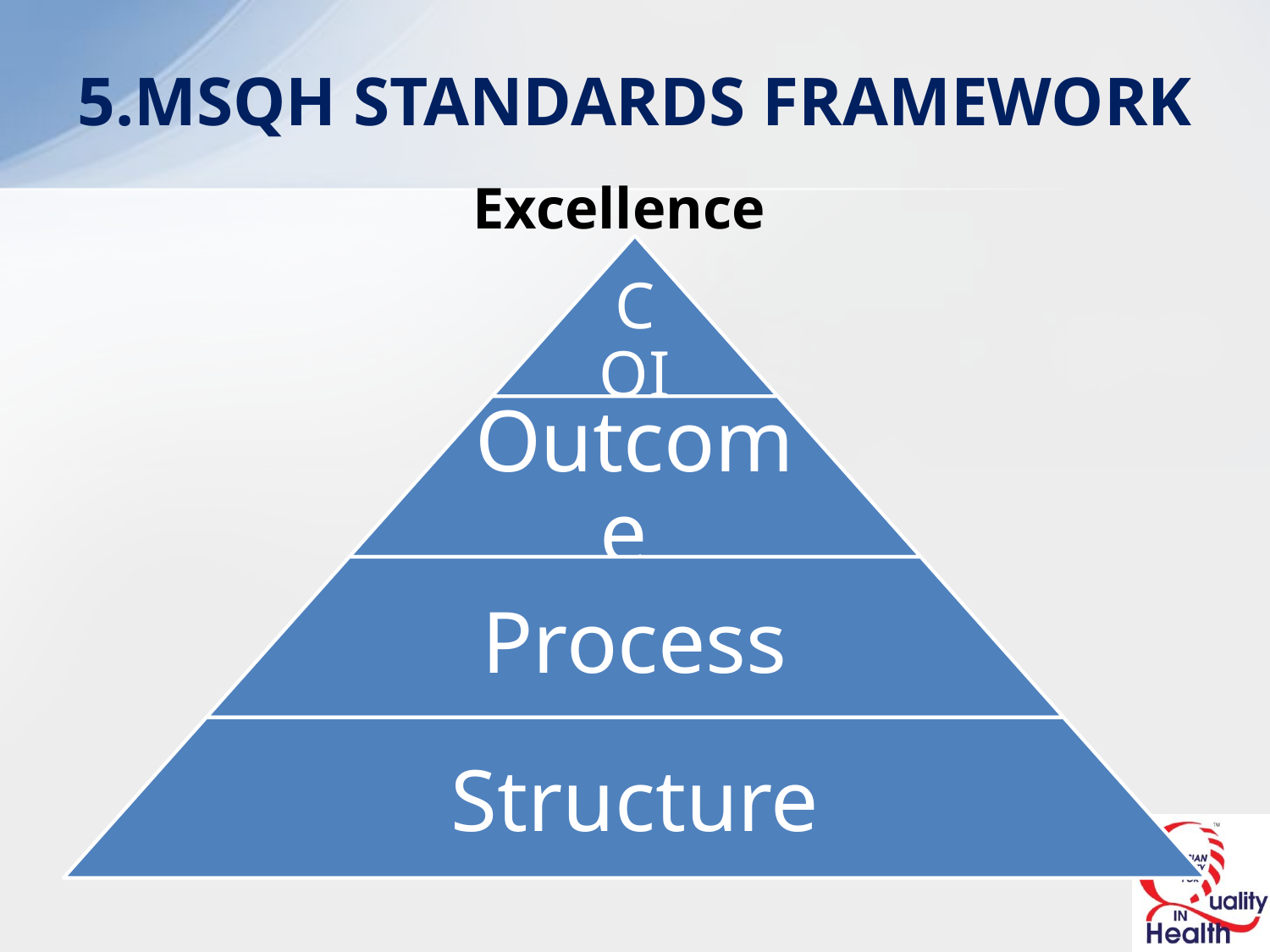

# 5.MSQH STANDARDS FRAMEWORK
Excellence

## Slide 20
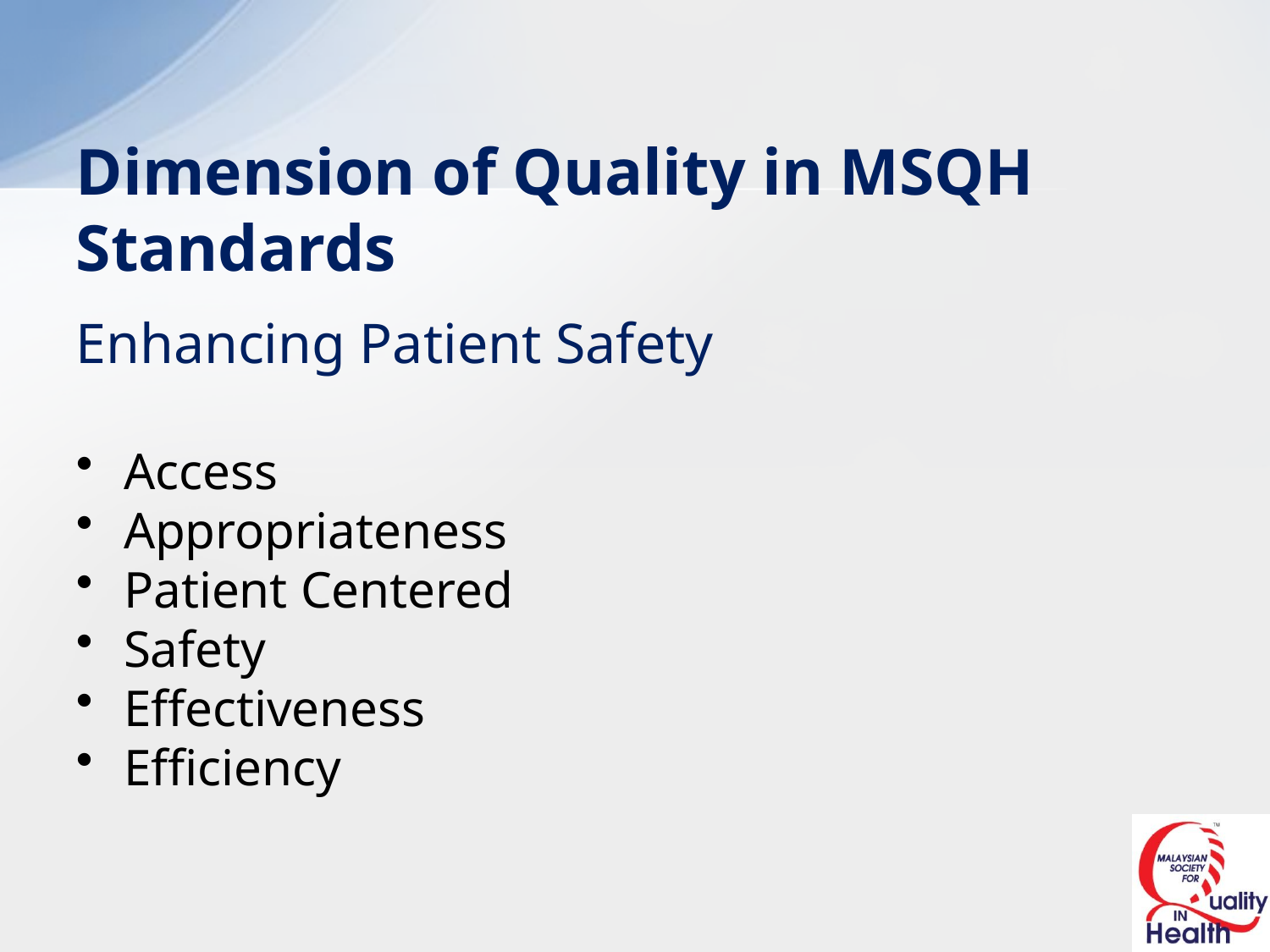

# Dimension of Quality in MSQH Standards Enhancing Patient Safety
Access
Appropriateness
Patient Centered
Safety
Effectiveness
Efficiency

## Slide 21
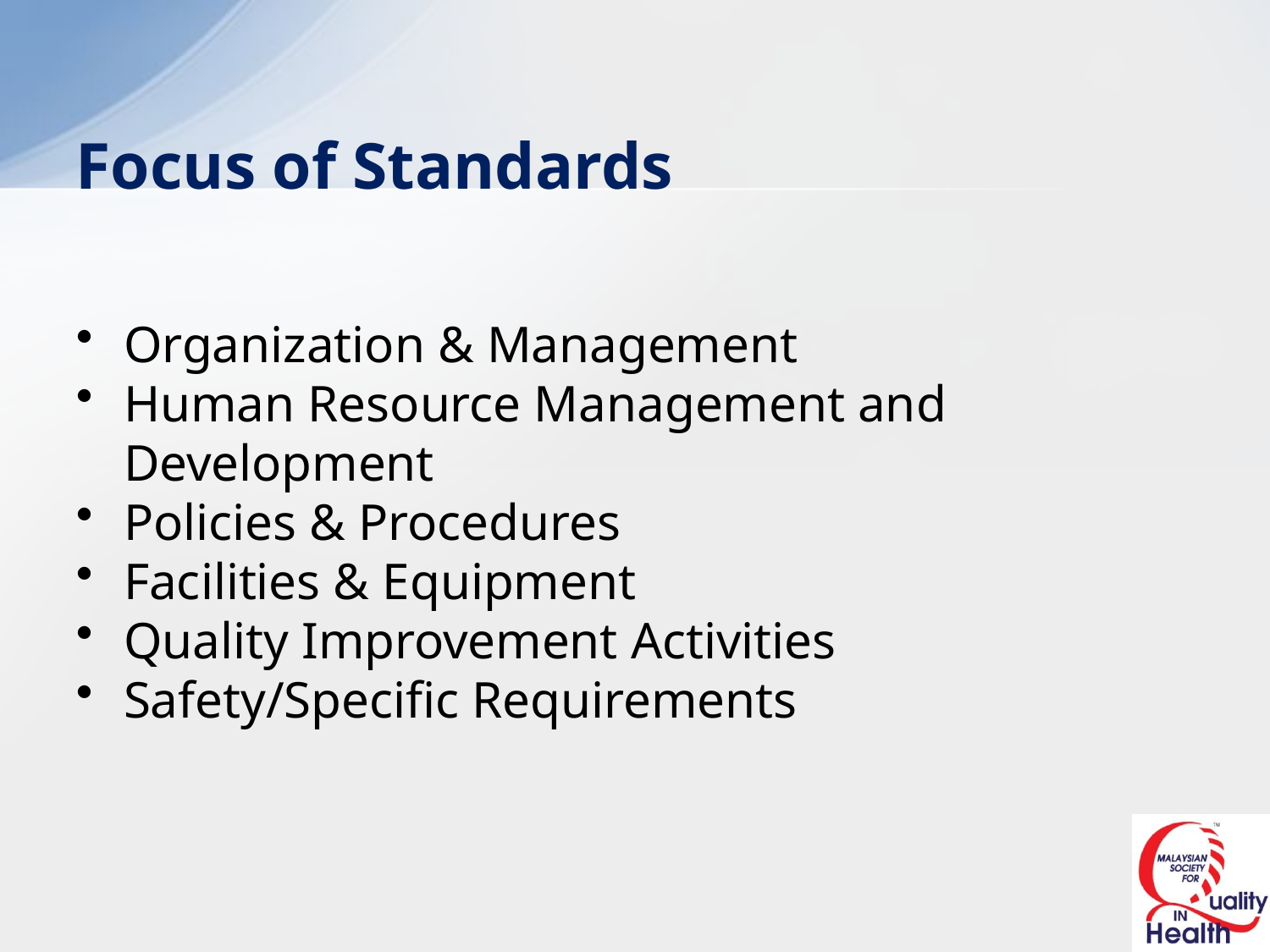

# Focus of Standards
Organization & Management
Human Resource Management and Development
Policies & Procedures
Facilities & Equipment
Quality Improvement Activities
Safety/Specific Requirements

## Slide 22
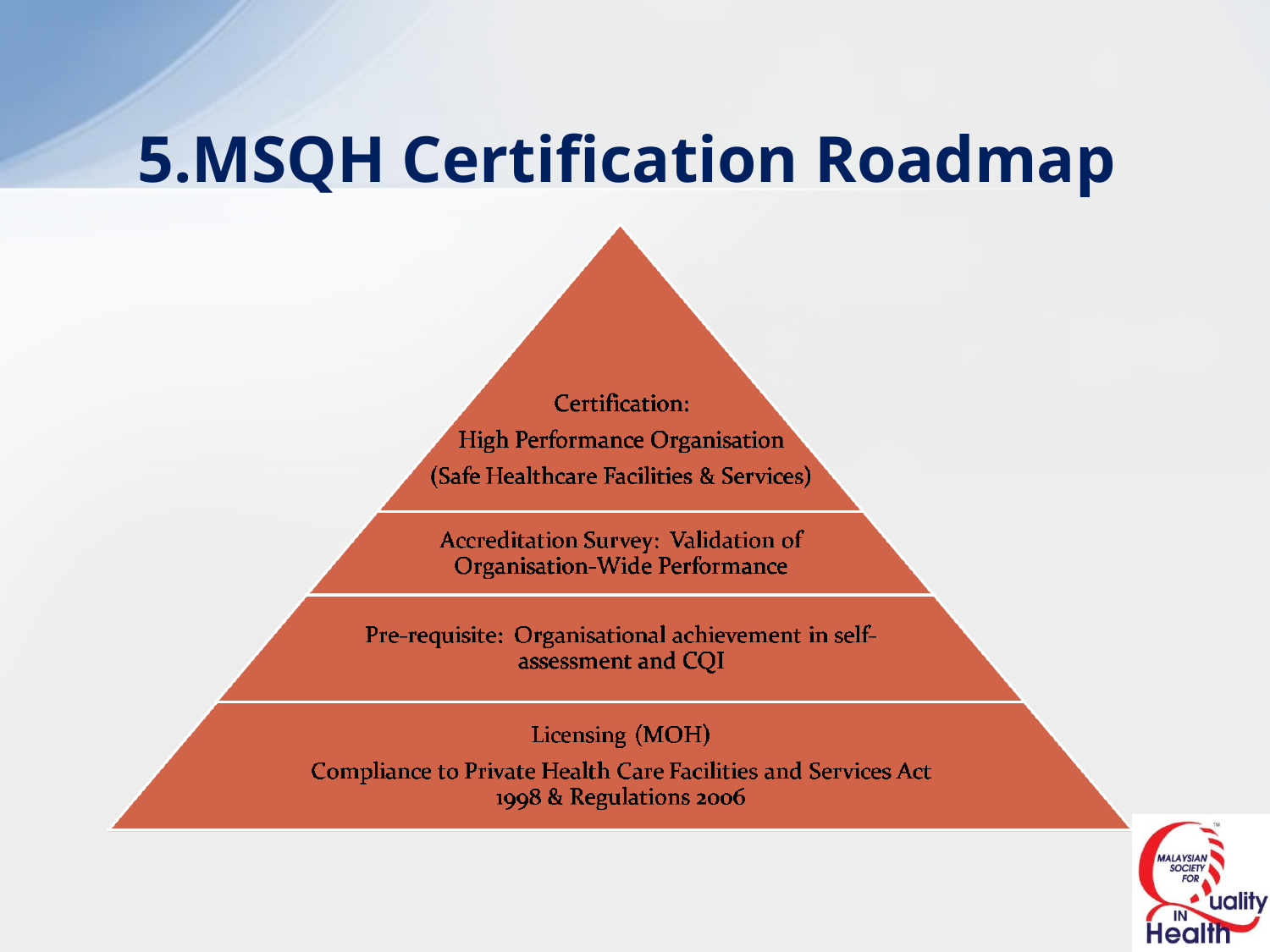

# 5.MSQH Certification Roadmap

## Slide 23
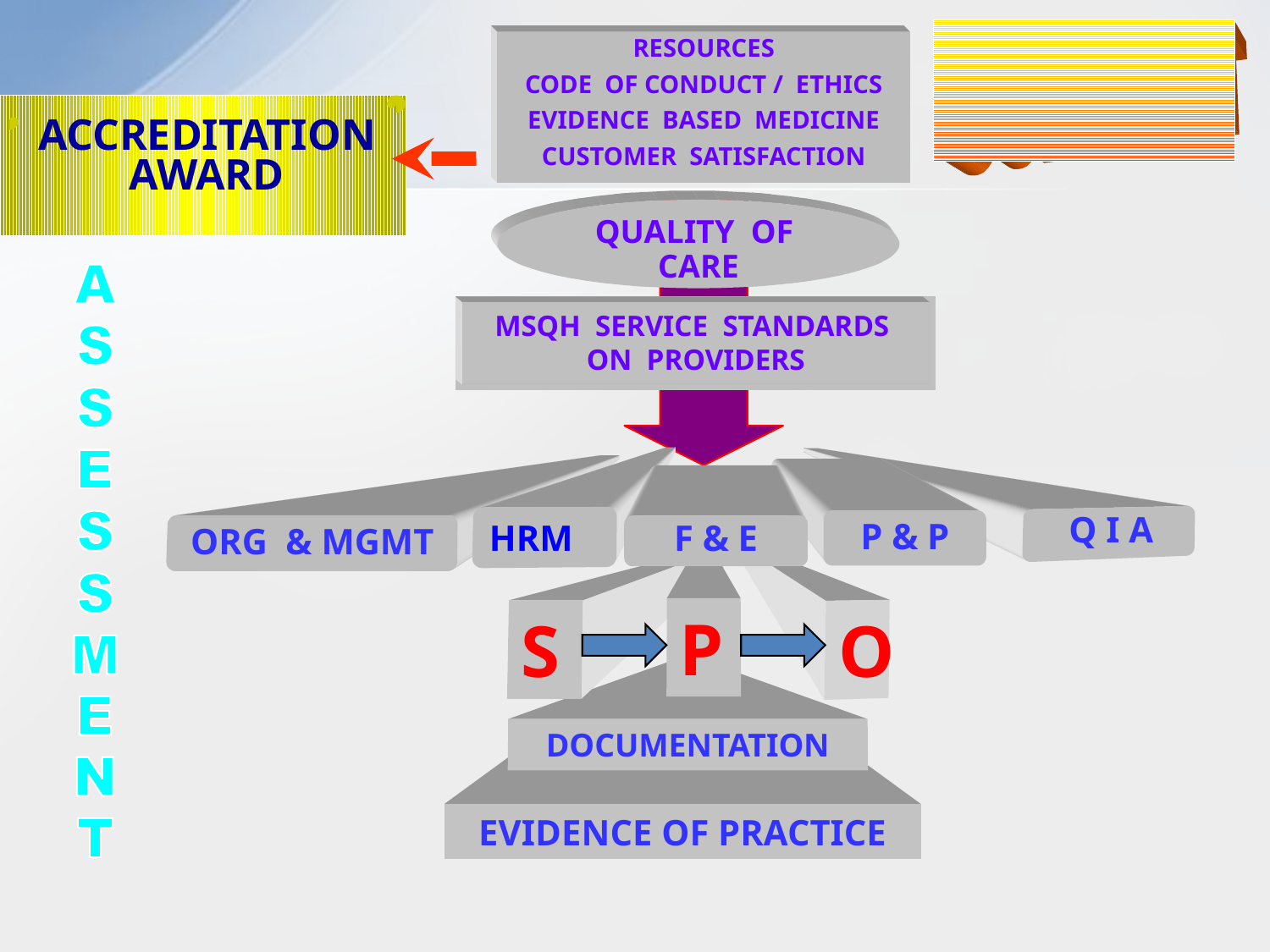

RESOURCES
CODE OF CONDUCT / ETHICS
EVIDENCE BASED MEDICINE
CUSTOMER SATISFACTION
ACCREDITATION AWARD
QUALITY OF CARE
MSQH SERVICE STANDARDS ON PROVIDERS
HRM
Q I A
ORG & MGMT
P & P
F & E
P
S
O
DOCUMENTATION
EVIDENCE OF PRACTICE

## Slide 24
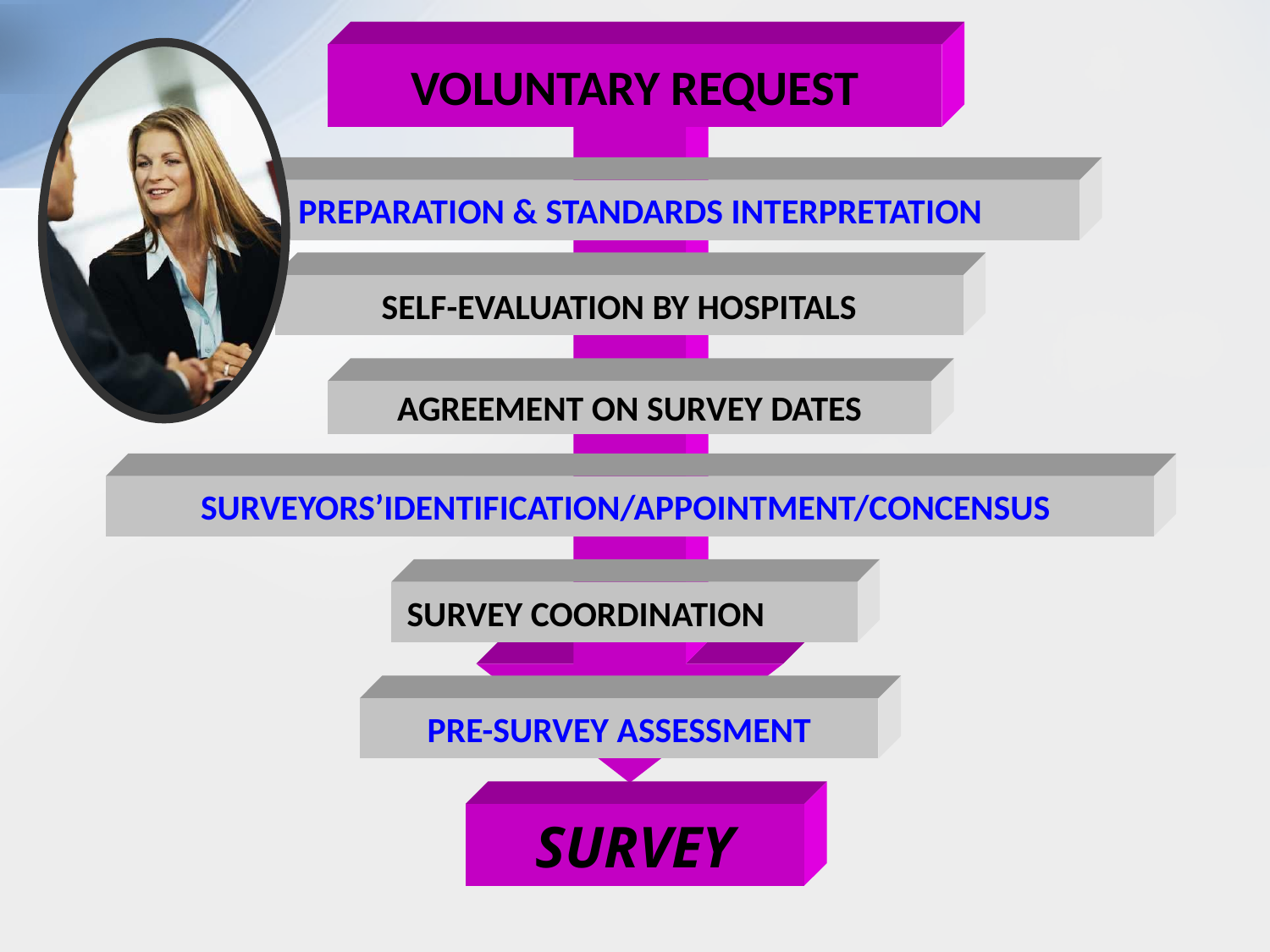

VOLUNTARY REQUEST
PREPARATION & STANDARDS INTERPRETATION
SELF-EVALUATION BY HOSPITALS
AGREEMENT ON SURVEY DATES
SURVEYORS’IDENTIFICATION/APPOINTMENT/CONCENSUS
SURVEY COORDINATION
PRE-SURVEY ASSESSMENT
SURVEY

## Slide 25
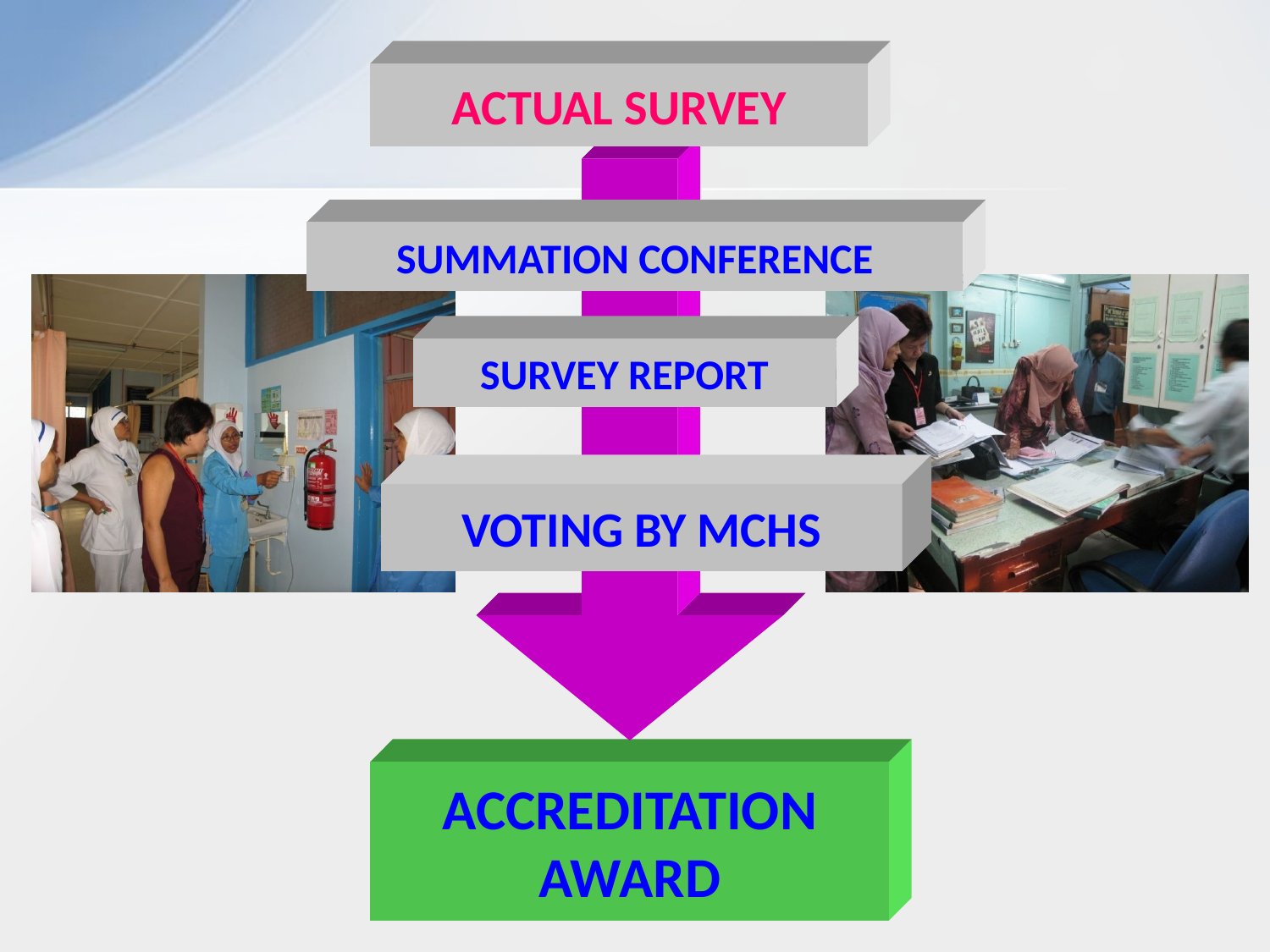

ACTUAL SURVEY
SUMMATION CONFERENCE
SURVEY REPORT
VOTING BY MCHS
ACCREDITATION AWARD

## Slide 26
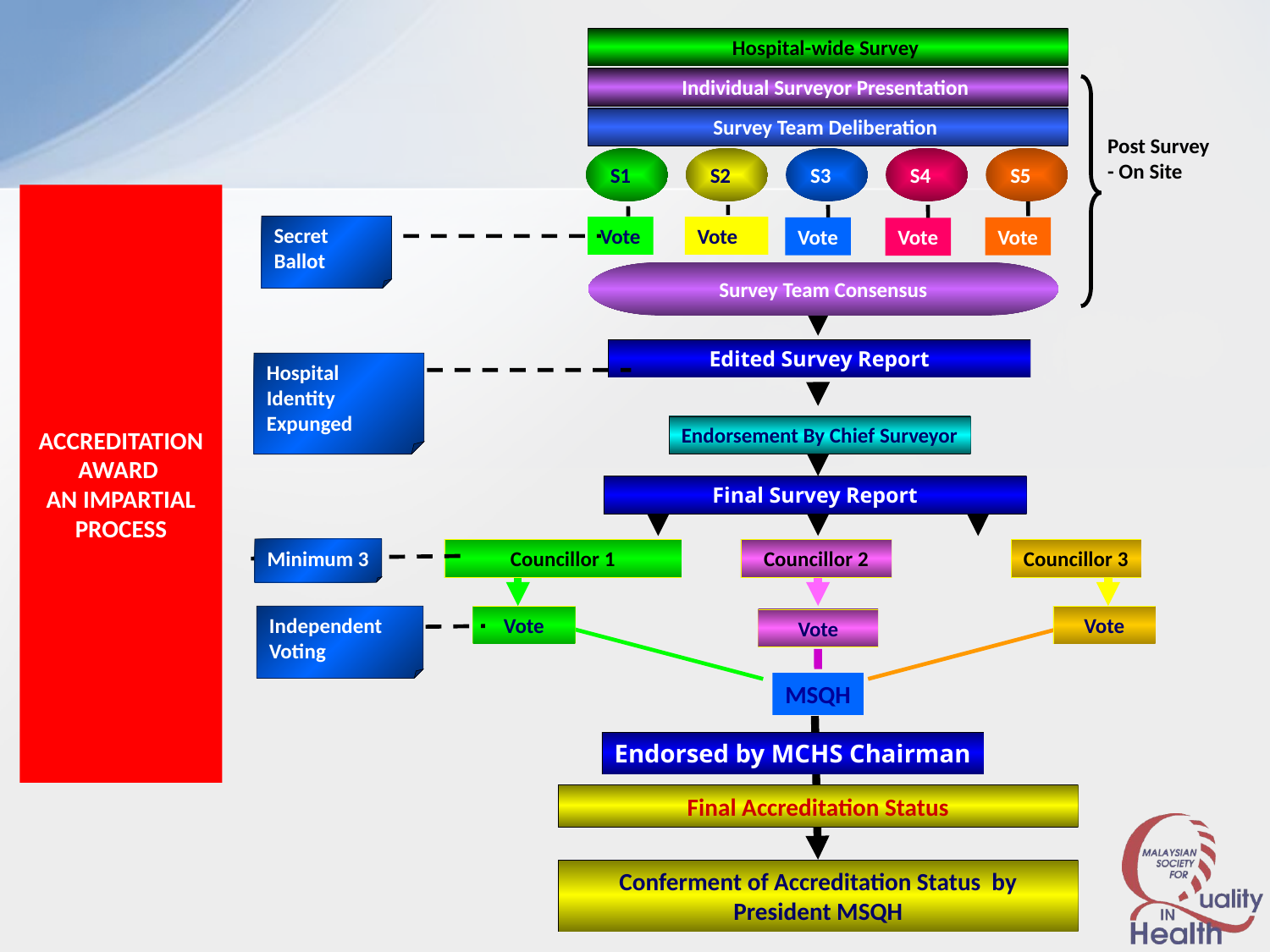

Hospital-wide Survey
Individual Surveyor Presentation
Post Survey
- On Site
Survey Team Deliberation
S1
S2
S3
S4
S5
Vote
Vote
Vote
Vote
Vote
ACCREDITATION AWARD
AN IMPARTIAL PROCESS
Secret Ballot
Survey Team Consensus
Edited Survey Report
Hospital Identity Expunged
Endorsement By Chief Surveyor
Final Survey Report
Councillor 1
Councillor 2
Councillor 3
Vote
Vote
Vote
Minimum 3
Independent Voting
MSQH
Endorsed by MCHS Chairman
Final Accreditation Status
Conferment of Accreditation Status by President MSQH

## Slide 27
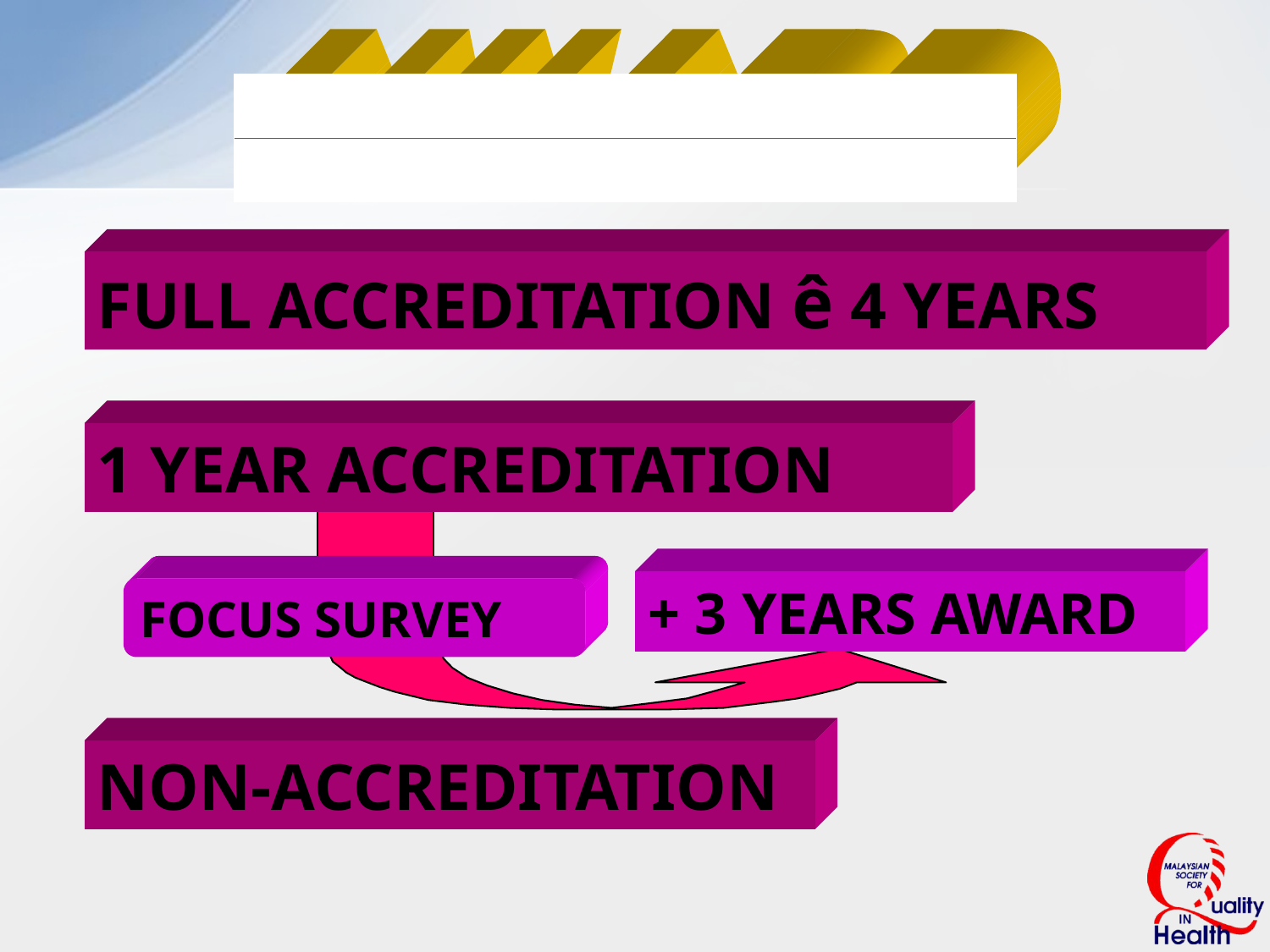

FULL ACCREDITATION ê 4 YEARS
1 YEAR ACCREDITATION
+ 3 YEARS AWARD
FOCUS SURVEY
NON-ACCREDITATION

## Slide 28
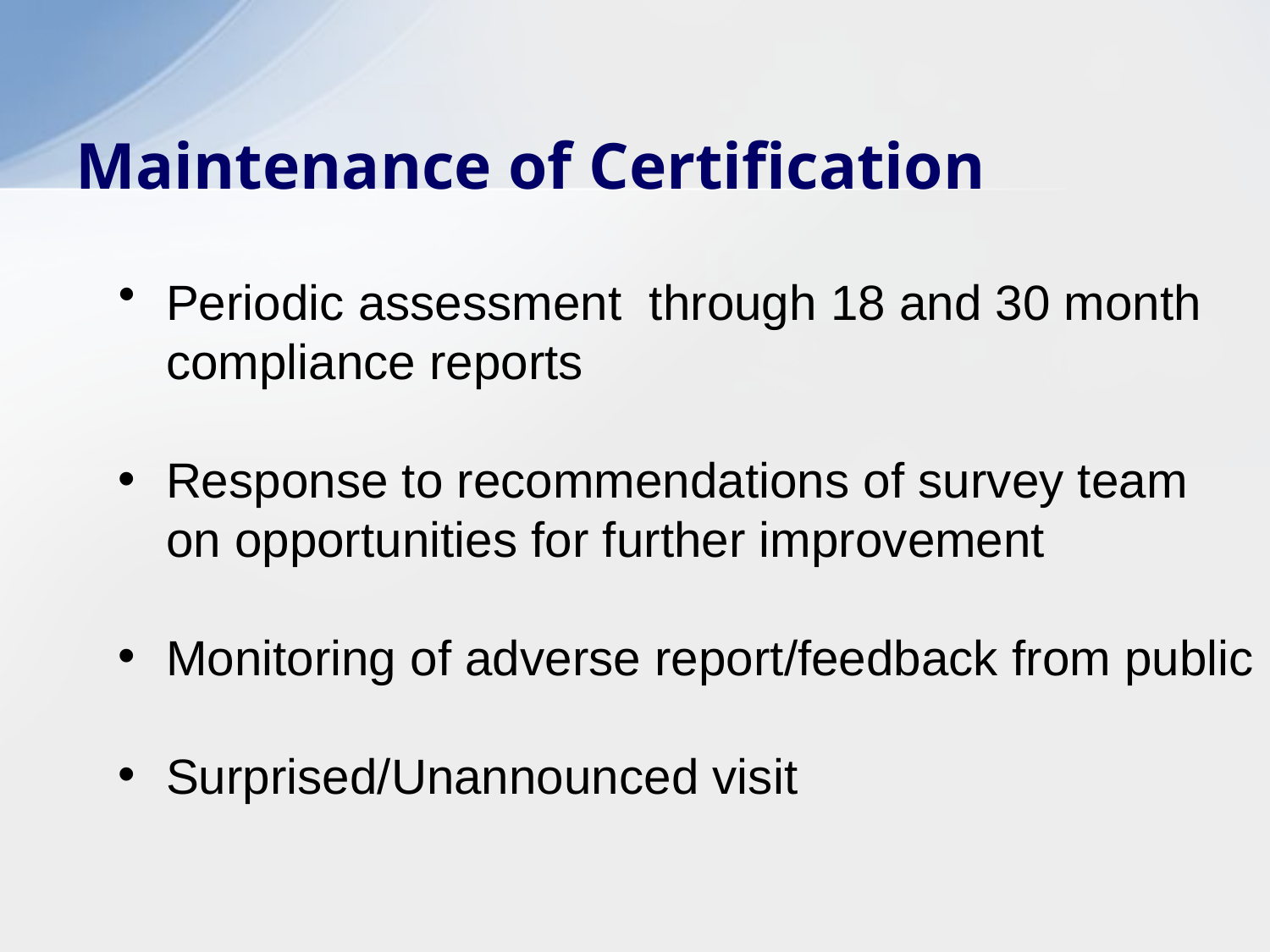

# Maintenance of Certification
Periodic assessment through 18 and 30 month compliance reports
Response to recommendations of survey team on opportunities for further improvement
Monitoring of adverse report/feedback from public
Surprised/Unannounced visit

## Slide 29
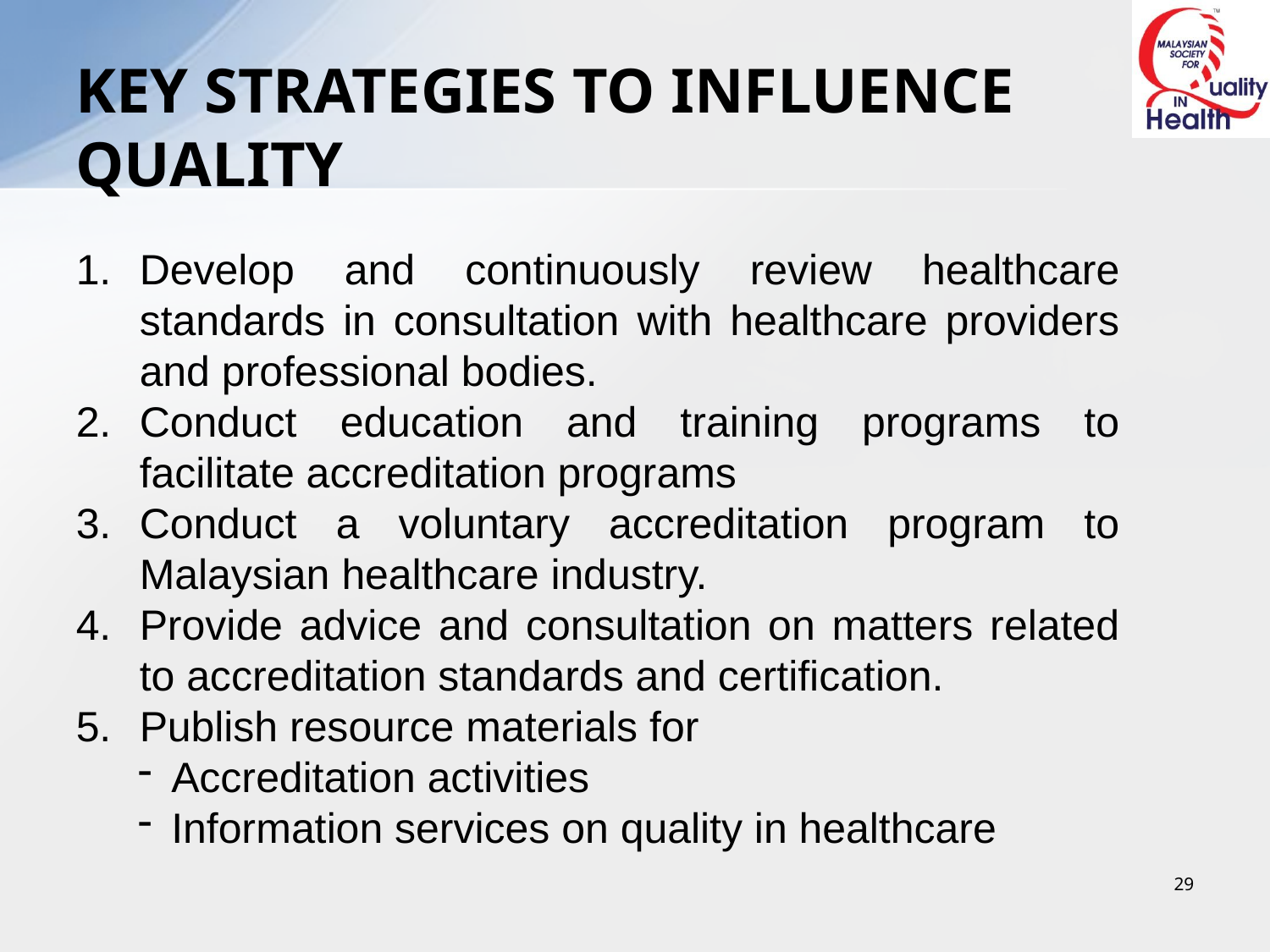

# KEY STRATEGIES TO INFLUENCE QUALITY
Develop and continuously review healthcare standards in consultation with healthcare providers and professional bodies.
Conduct education and training programs to facilitate accreditation programs
Conduct a voluntary accreditation program to Malaysian healthcare industry.
Provide advice and consultation on matters related to accreditation standards and certification.
Publish resource materials for
Accreditation activities
Information services on quality in healthcare
29

## Slide 30
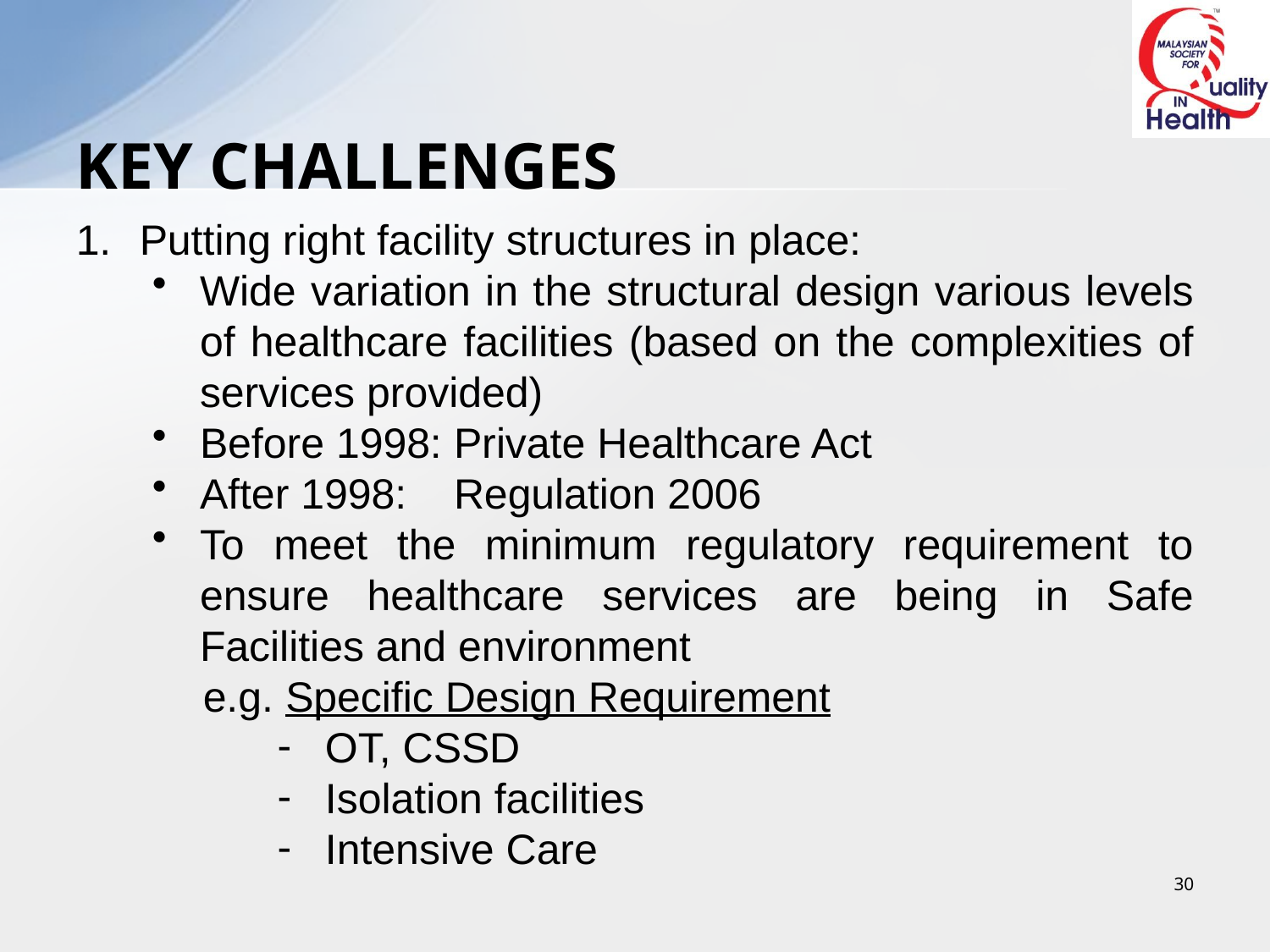

# KEY CHALLENGES
Putting right facility structures in place:
Wide variation in the structural design various levels of healthcare facilities (based on the complexities of services provided)
Before 1998:	Private Healthcare Act
After 1998:	Regulation 2006
To meet the minimum regulatory requirement to ensure healthcare services are being in Safe Facilities and environment
	e.g. Specific Design Requirement
OT, CSSD
Isolation facilities
Intensive Care
30

## Slide 31
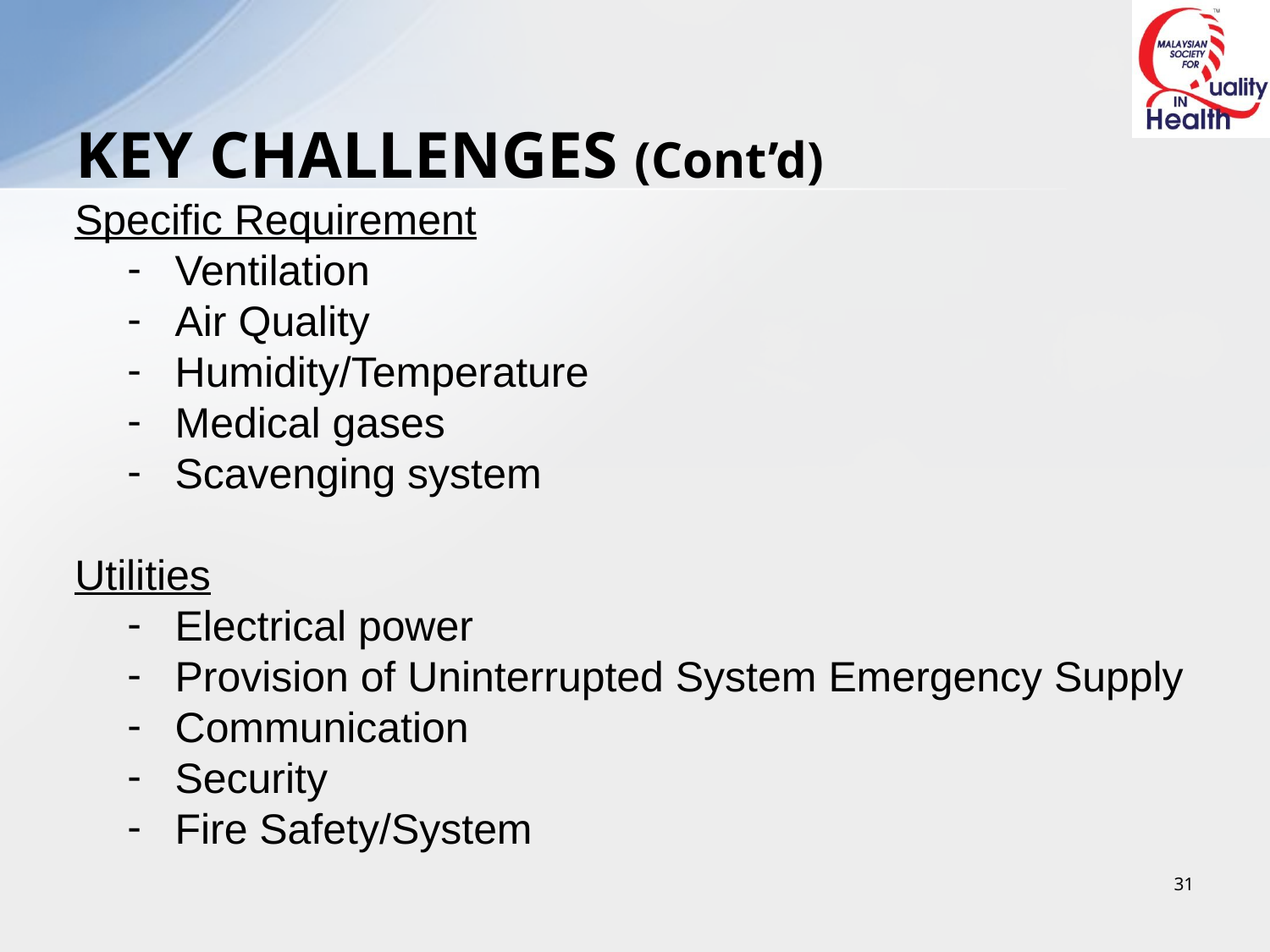

# KEY CHALLENGES (Cont’d)
Specific Requirement
Ventilation
Air Quality
Humidity/Temperature
Medical gases
Scavenging system
Utilities
Electrical power
Provision of Uninterrupted System Emergency Supply
Communication
Security
Fire Safety/System
31

## Slide 32
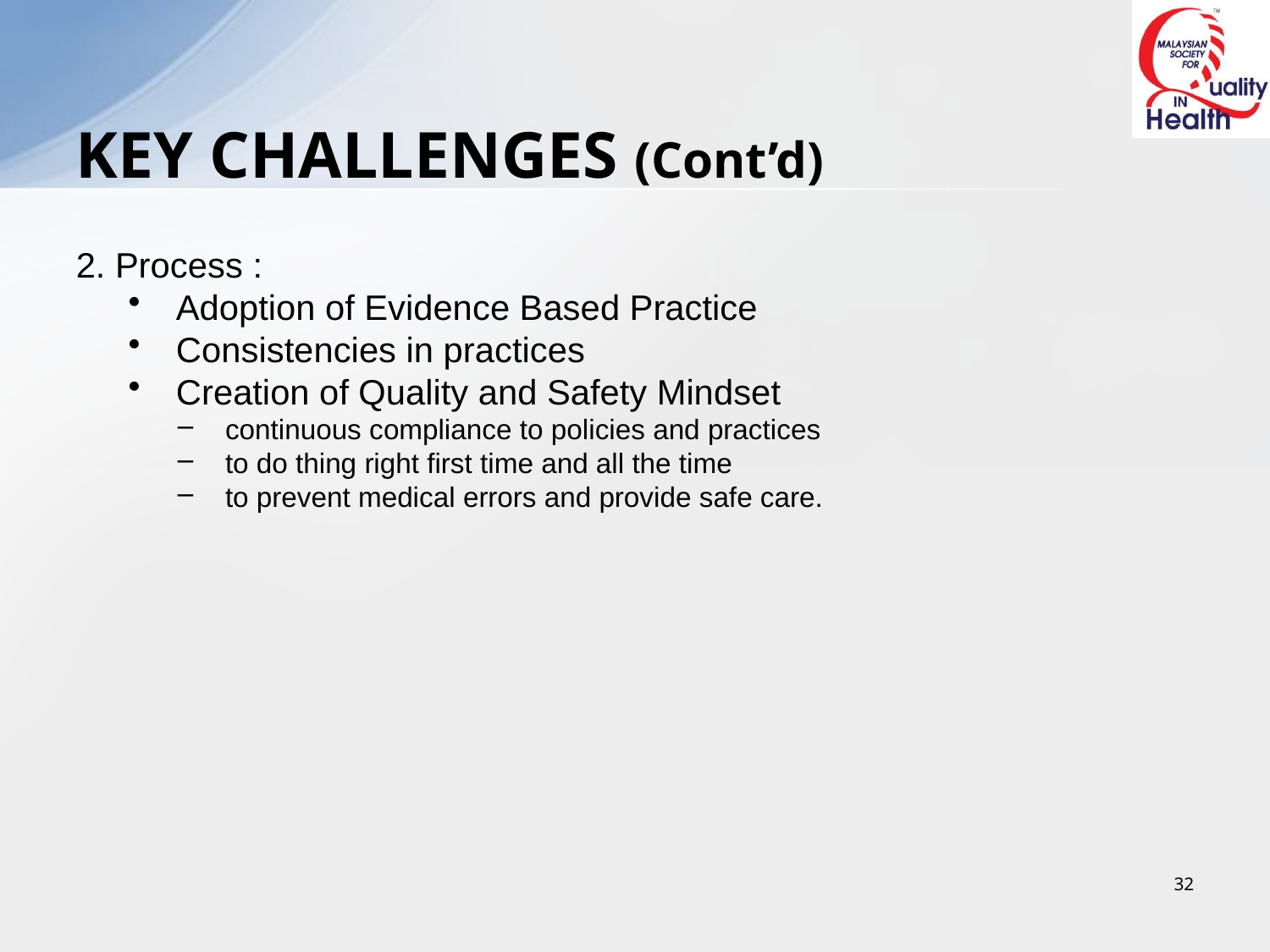

# KEY CHALLENGES (Cont’d)
2. Process :
Adoption of Evidence Based Practice
Consistencies in practices
Creation of Quality and Safety Mindset
continuous compliance to policies and practices
to do thing right first time and all the time
to prevent medical errors and provide safe care.
32

## Slide 33
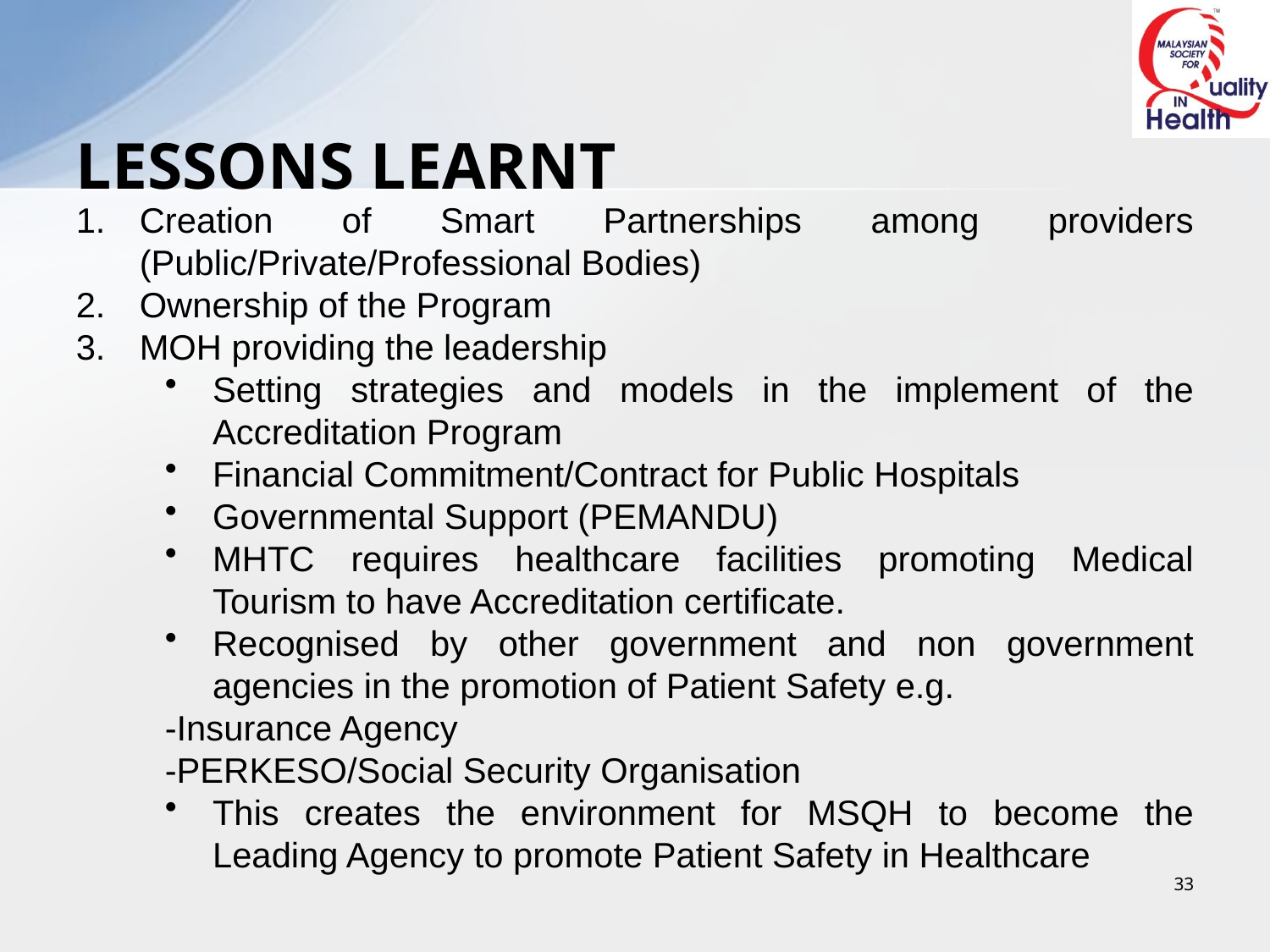

# LESSONS LEARNT
Creation of Smart Partnerships among providers (Public/Private/Professional Bodies)
Ownership of the Program
MOH providing the leadership
Setting strategies and models in the implement of the Accreditation Program
Financial Commitment/Contract for Public Hospitals
Governmental Support (PEMANDU)
MHTC requires healthcare facilities promoting Medical Tourism to have Accreditation certificate.
Recognised by other government and non government agencies in the promotion of Patient Safety e.g.
-Insurance Agency
-PERKESO/Social Security Organisation
This creates the environment for MSQH to become the Leading Agency to promote Patient Safety in Healthcare
33

## Slide 34
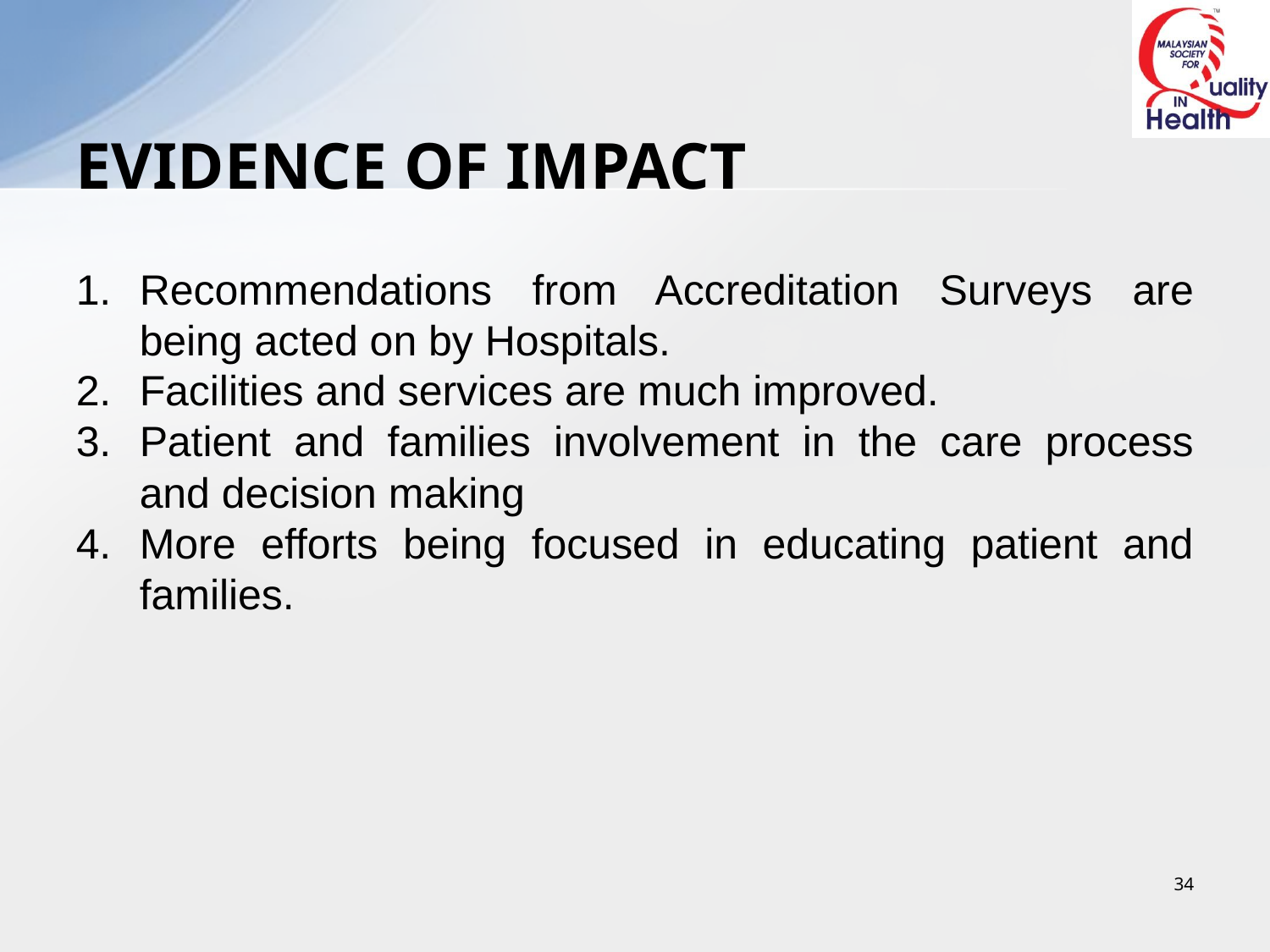

# EVIDENCE OF IMPACT
Recommendations from Accreditation Surveys are being acted on by Hospitals.
Facilities and services are much improved.
Patient and families involvement in the care process and decision making
More efforts being focused in educating patient and families.
34

## Slide 35
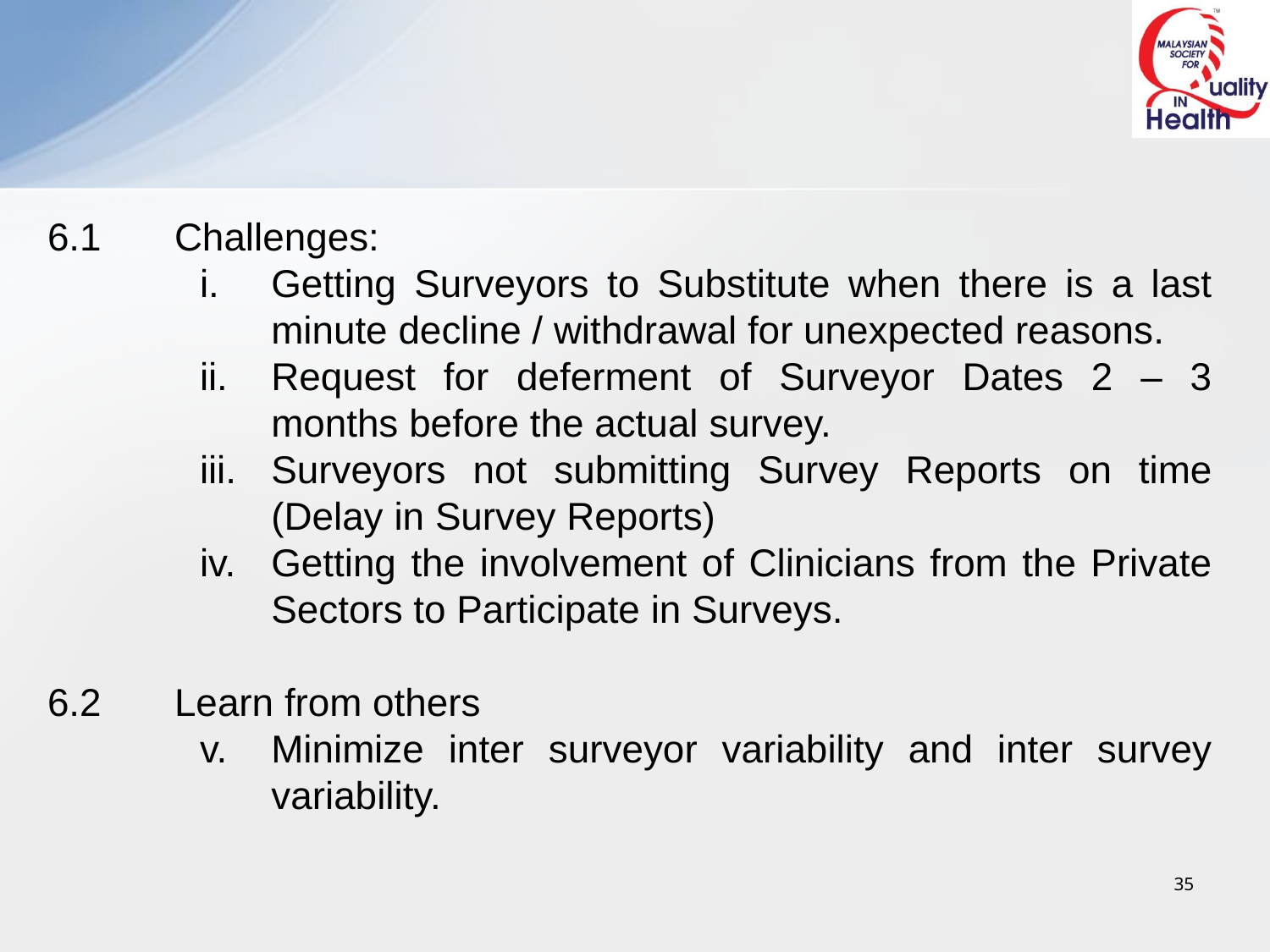

6.1	Challenges:
Getting Surveyors to Substitute when there is a last minute decline / withdrawal for unexpected reasons.
Request for deferment of Surveyor Dates 2 – 3 months before the actual survey.
Surveyors not submitting Survey Reports on time (Delay in Survey Reports)
Getting the involvement of Clinicians from the Private Sectors to Participate in Surveys.
6.2	Learn from others
Minimize inter surveyor variability and inter survey variability.
35

## Slide 36
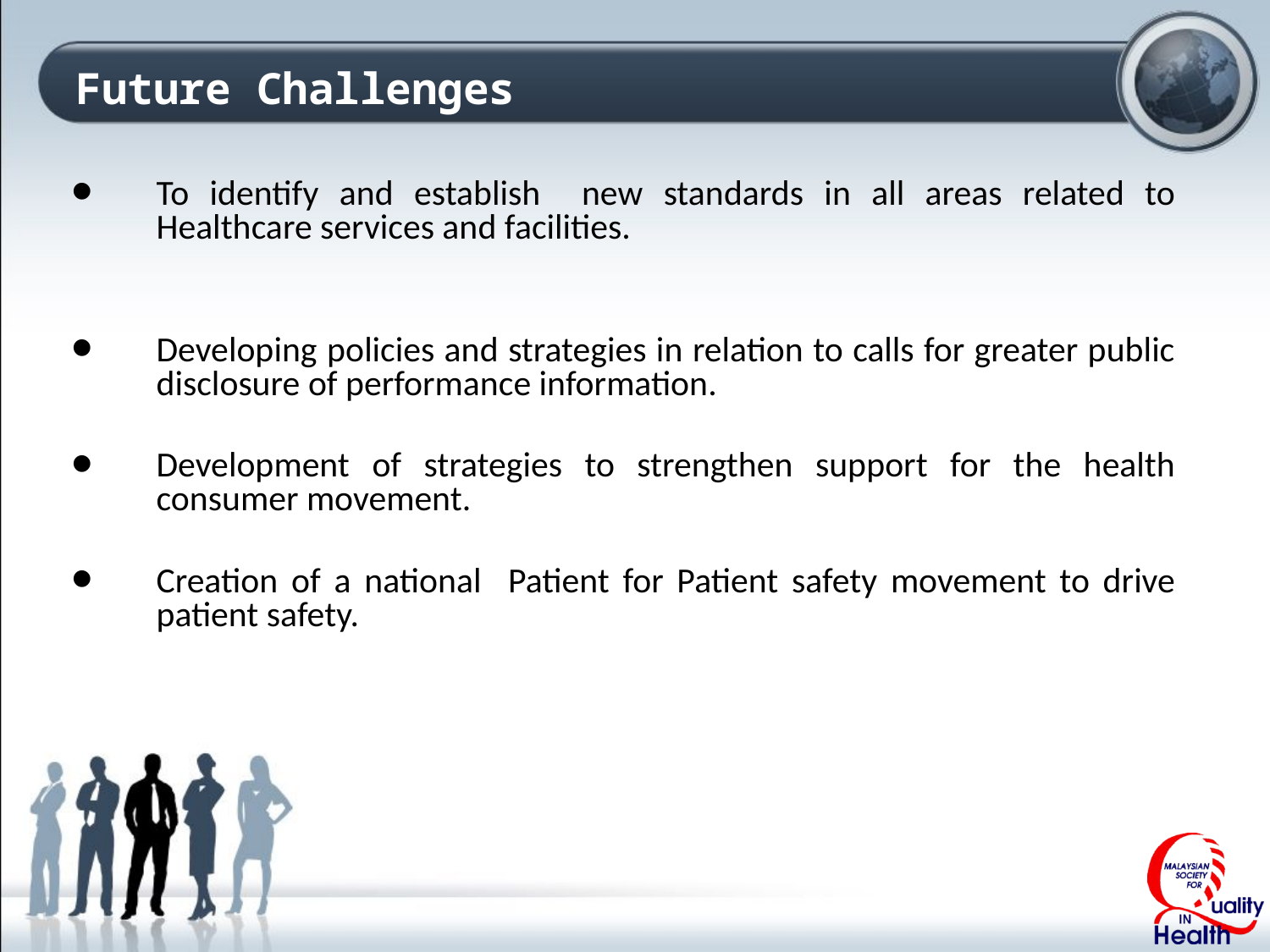

# Future Challenges
To identify and establish new standards in all areas related to Healthcare services and facilities.
Developing policies and strategies in relation to calls for greater public disclosure of performance information.
Development of strategies to strengthen support for the health consumer movement.
Creation of a national Patient for Patient safety movement to drive patient safety.

## Slide 37
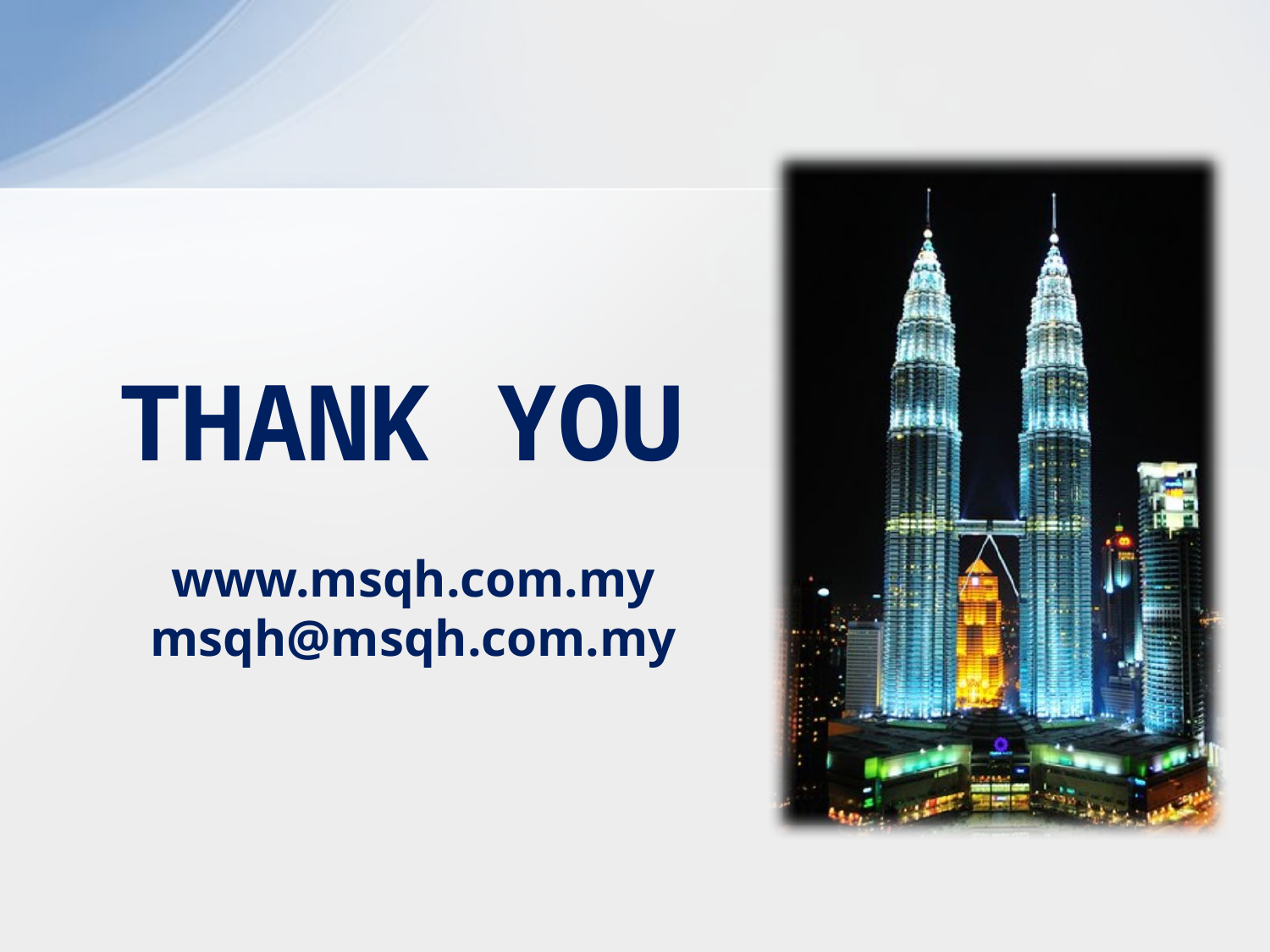

THANK YOU
www.msqh.com.my
msqh@msqh.com.my
