# Supplementary material for: Hospital accreditation: lessons from low- and middle-income countries
Source: Global Health. 2014 Sep 4;10:65. doi: 10.1186/s12992-014-0065-9 (PMC4159532; doi:10.1186/s12992-014-0065-9)
Supplement: Additional file 3: — Hospital Standards, Thailand. [file 12992_2014_65_MOESM3_ESM.pdf]

Healthcare Organization Accreditation Standards

For the 60th Anniversary Celebration of
His Majesty's Accession to the Throne

ที่ รล ๐๐๐๓.๔/๒๗๑๗๓ สำนักราชเลขาธิการ

พระบรมมหาราชวัง กทม. ๑๐๒๐๐

๒๕ ธันวาคม ๒๕๔๙

เรื่อง พระราชทานชื่อมาตรฐานโรงพยาบาล

เรียน ผู้อำนวยการสถาบันพัฒนาและรับรองคุณภาพโรงพยาบาล

อ้างถึง หนังสือสถาบันพัฒนาและรับรองคุณภาพโรงพยาบาล ที่ สวรส.พรพ. ๓๑๖/๒๕๔๙
ลงวันที่ ๖ กุมภาพันธ์ ๒๕๔๙

สิ่งที่ส่งมาด้วย แบบตราสัญลักษณ์การจัดงานฉลองสิริราชสมบัติครบ ๖๐ ปี

ตามหนังสือที่อ้างถึง แจ้งว่า สถานบันพัฒนาและรับรองคุณภาพโรงพยาบาล (พรพ.) ได้ปรับปรุงมาตรฐานโรงพยาบาล โดยนำมาตรฐานการเสริมสร้างสุขภาพและเกณฑ์รางวัลคุณภาพแห่งชาติ เข้ามาบูรณาการกับมาตรฐานโรงพยาบาลที่ใช้อยู่เดิม และจะประกาศใช้ในปี พ.ศ. ๒๕๔๙ เพื่อเฉลิมพระเกียรติ เนื่องในโอกาสฉลองสิริราชสมบัติครบ ๖๐ ปี จึงขออนุญาตใช้ชื่อและตราสัญลักษณ์การจัดงานฉลองสิริราชสมบัติ ครบ ๖๐ ปี ประดับที่ปกเอกสารฉบับดังกล่าว ความแจ้งอยู่แล้ว นั้น

สำนักราชเลขาธิการพิจารณาแล้ว เห็นสมควรให้ใช้ชื่อมาตรฐานโรงพยาบาลฉบับดังกล่าวว่า "มาตรฐานโรงพยาบาลและบริการสุขภาพ ฉบับเฉลิมพระเกียรติฉลองสิริราชสมบัติครบ ๖๐ ปี" และอนุญาตให้เชิญตราสัญลักษณ์การจัดงานฉลองสิริราชสมบัติครบ ๖๐ ปี ประดับที่ปกเอกสาร ซึ่งความทราบฝ่าละอองธุลีพระบาทแล้ว

จึงเรียนมาเพื่อทราบ ทั้งนี้ ตราสัญลักษณ์ ฯ จะต้องมีรูปแบบและสีถูกต้องตามที่ทางราชการกำหนด ตามที่แนบมาพร้อมนี้

ขอแสดงความนับถือ

(ท่านผู้หญิงบุตรี วีระไวทยะ)
รองราชเลขาธิการ ปฏิบัติราชการแทน
ราชเลขาธิการ

กองการในพระองค์
โทร.๐๒-๒๒๕๓๔๕๗-๖๒ ต่อ ๒๓๐๘ - ๒๓๑๑, ๒๓๑๓
โทรสาร ๐๒-๒๒๔๓๒๖๔

This letter was from Office of His Majesty's Principal Private Secretary to the director of the Institute of Hospital Quality Improvement & Accreditation (HA-Thailand) as a response to the request from HA-Thailand in using the name and the Royal emblem of the Sixtieth Anniversary Cerebrations of His Majesty the King's Accession to the Throne to be stamped upon the cover of HA-Thailand Standard Book. The Office proposed using the name "Hospital and Health Care Standard: Version Sixtieth Anniversary Cerebrations of His Majesty the King's Accession to the Throne" and permitted, under the recognition of His Majesty's the King, stamping the Royal emblem upon the cover of the Standard Book.

Preface

Healthcare Organization Accreditation Standards: Version Sixtieth Anniversary Cerebrations of His Majesty's the King Accession to the Throne, which was revised and completed in year B.E. 2549, derived from 3 valuable standards/criteria. These include the first version of HA-Thailand Standard which has been used since B.E. 2540, Health Promotion Standard from Department of Public Health and toward performance excellence.

Integrating these 3 standards/criteria together reduced duplication of details of each standard/criterion. As a result, hospitals can integrate health promotion and health care activities together and establish a platform for directing their staff toward excellent organizations.

HA-Thailand has received His Majesty's the King compassion in granting the name for this new version of HA-Thailand Standard and permitted using of the Royal Ceremonial Emblem in Commemoration of the Sixtieth Anniversary Celebrations of His Majesty's the King Accession to the Throne to be stamped upon the cover of the Standard. His Majesty's benevolence is a highly auspicious motivation for processes of hospital and health care development in Thailand.

The Institute of Hospital Quality Improvement & Accreditation
December B.E. 2549

Contents

Praface ... iii
Guideline for using this standard ... 1
PART I ORGANIZATION MANAGEMENT OVERVIEW ... 3
I-1 Leadership ... 4
I-2 Strategic Management ... 8
I-2 Strategic Management ... 9
I-3 Focus on Patients / Customers ... 12
I-3 Focus on Patients / Customers (PCF) ... 13
I-4 Measurement, Analysis and Knowledge Management ... 18
I-5 Human Resource Focus ... 22
I-6 Process Management ... 30
PART II KEY HOSPITAL SYSTEMS ... 34
II - 1 Risk, Safety, and Quality Management ... 36
II - 2 Professional Governance ... 42
II - 3 Environment of Care ... 46
II - 4 Infection Control and Prevention ... 54
II - 5 Medical Record System ... 58
II - 6 Medication Management System ... 62
II - 7 Diagnostic Investigation and Related Services ... 68
II – 8 Disease and Health Hazard Surveillance ... 74
II – 9 Working with Community ... 78
PART III PATIENT CARE PROCESSES ... 82
III – 1 Access and Entry ... 84
III - 2 Patient Assessment ... 86
III - 3 Planning ... 88
III - 4 Patient Care Delivery ... 92
III - 5 Information and Empowerment for Patient / Family ... 98
III - 6 Continuity of Care ... 101
PART IV ORGANIZATION PERFORMANCE RESULTS ... 102
IV-1 Patient Care Results ... 103
IV-2 Patient and Other Customer-Focused Results ... 103
IV-3 Financial Results ... 103
IV-4 Human Resource Results ... 103
IV-5 Key System & Process Operational Results ... 103
IV-6 Leadership Results ... 104
IV-7 Health Promotion Results ... 104

Guideline for using this Standard

objective of this Standard:

To be a guideline for designing appropriate work systems and to be an evaluation tool for determining opportunities for improvement.

Outline of this Standard:

This Standard can be used for hospitals' system development and performance evaluation. It can be applied to every level of hospital. It also mentions desirable health promotion activities in hospitals.

This Standard can also be used for health care facilities other than hospitals by focusing on codes of practices in part 1 and 4 of the Standard and selectively applying relevant codes of practices in part 2 and 3.

Matters that must be concurrently considered when using this Standard:

1. Contexts of organizations and service units, especially problems, challenges and important risks
2. Core values & concepts of quality improvement and health promotion
    a. Organization direction : visionary leadership, system perspective, agility
    b. Customer : patient & customer focus, focus on health, community responsibility
    c. Staff : value on staff, individual commitment, teamwork, ethic & professional standard
    d. Development ; creativity & innovation, management by fact, continuous process improvement, focus on results, evidence-based approach
    e. Learning and empowerment
3. Quality improvement and learning cycle (Plan-Do-Study-Act or Purpose-Process-Performance)
4. A scoring guideline for evaluating levels of compliance with the Standard

Using this Standard to lead to quality

1. Clarify targets and key words in the Standard
2. Emphasize on using the Standard for organization learning and performance improvement
3. Emphasize on linkages between components within each system (study and understand lines in diagrams that link various components together) and also linkage between different systems
4. Emphasize on using the Standard for learning and reviewing practices in real workplaces and at bed sides
5. Emphasize on integrated development that encompass all work areas
6. Appropriately apply the Standard - such as applying part 1 of the Standard to service units, apply the Standard on process management to all matters
7. Emphasize on evaluation of the overall picture of quality improvement and also achievement of targets of each system

PART I ORGANIZATION MANAGEMENT OVERVIEW

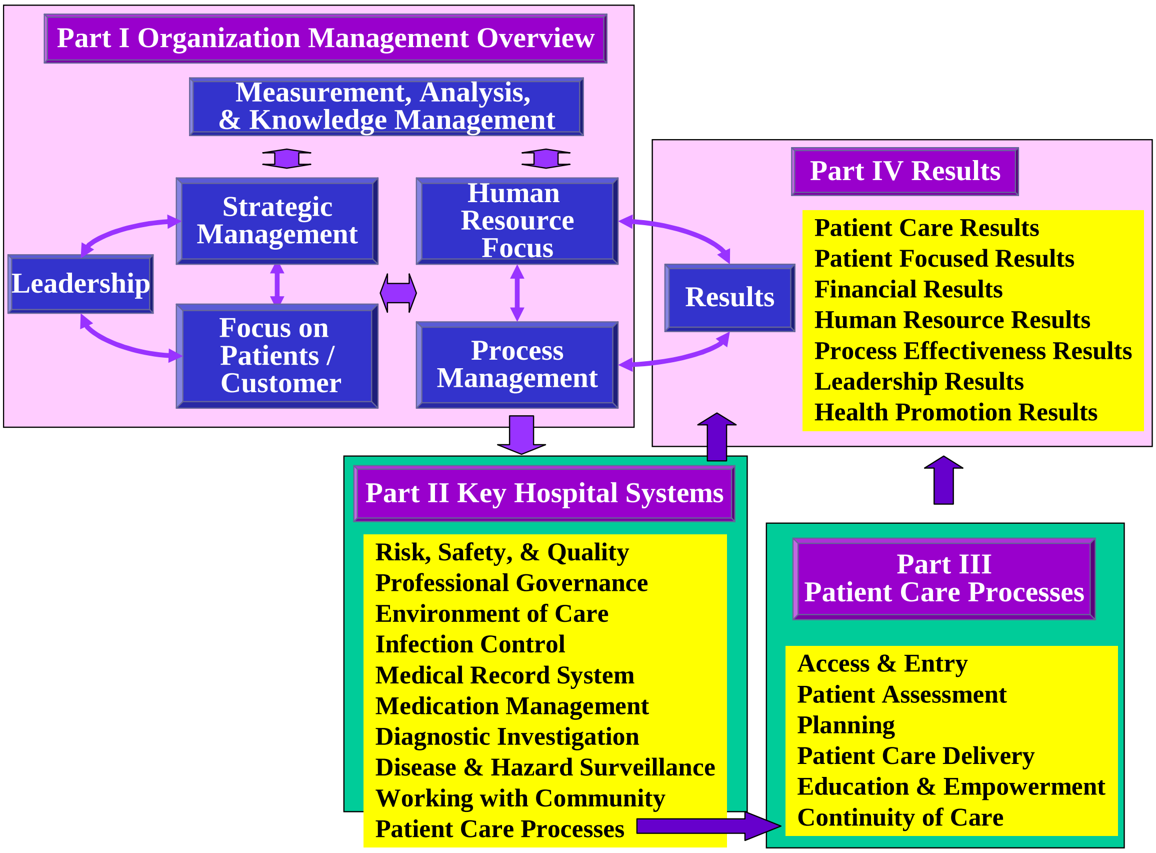

I-1 Leadership

I – 1.1 Senior Leadership

Senior leaders guide the organization. Senior leaders communicate with staff and encourage good performance, ensure quality and safety of care.

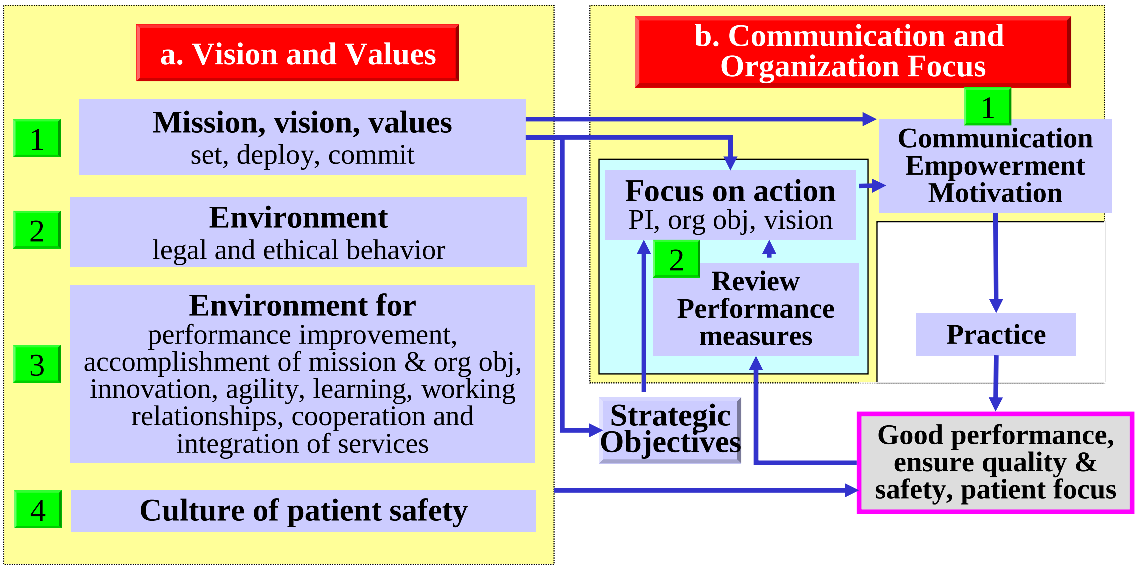

Part I Organization Management Overview

1.1 Senior Leadership

Senior leaders guide the organization. Senior leaders communicate with staff and encourage good performance, ensure quality and safety of care.

a. Vision and Values

(1) Senior leaders set organizational mission, vision and values. Senior leaders deploy mission, vision and values through the leadership system to all staff and key partners. Senior leaders' personal actions reflect a commitment to the organization's values.

(2) Senior leaders personally promote an organizational environment that fosters, requires, and results in legal and ethical behavior.

(3) Senior leaders create an environment for performance improvement, the accomplishment of mission and organization objectives, innovation, organizational agility, organizational and staff learning, effective working relationships, cooperation and integration of services.

(4) Senior leaders create and promote a culture of patient safety.

b. Communication and Organization Focus

(1) Senior leaders communicate with, empower, and motivate all staff throughout the organization. Senior leaders encourage frank, two-way communication throughout the organization. Senior leaders take an active role in staff reward and recognition to reinforce a focus on patients / other customers, quality and safety in patient care, and good performance.

(2) Senior leaders create a focus on action to improve performance, accomplish the organization's objectives, and attain its vision; including performance expectation in those areas. Senior leaders regularly review performance measures to inform the needed action.

I – 1.2 Governance and Social Responsibility

The organization demonstrates its good governance and its responsibilities to the public, ensures ethical behavior, and contributes to the health of key communities.

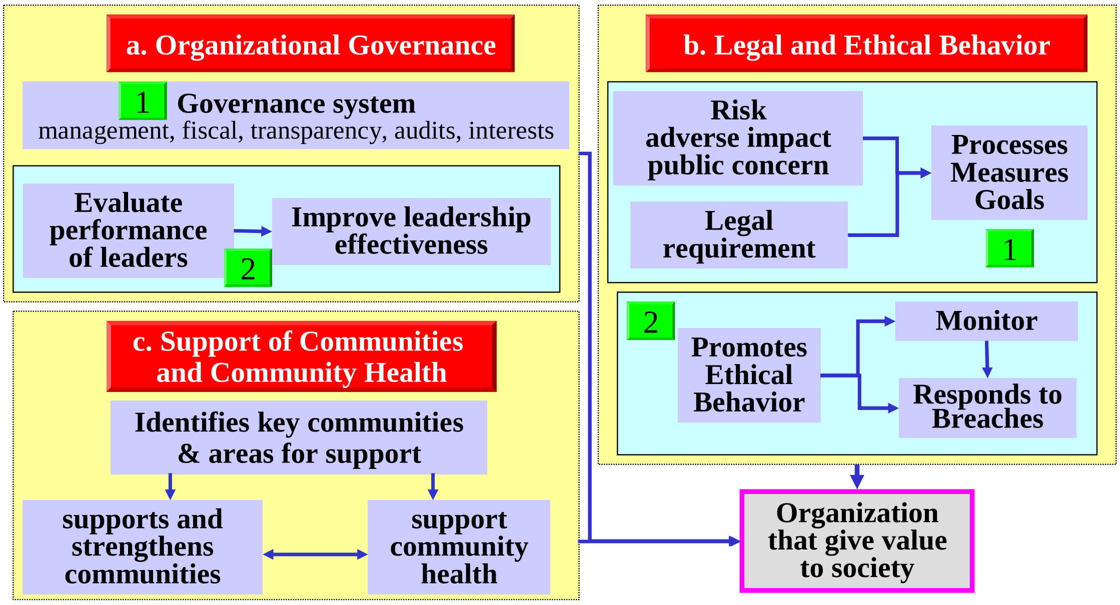

1.2 Governance and Social Responsibility

The organization demonstrates its good governance and its responsibilities to the public, ensures ethical behavior, and contributes to the health of key communities.

a. Organizational Governance

(1) The organization reviews and achieves the following key aspects of its governance system: accountability for management's actions, fiscal accountability, transparency in operation, independence in internal and external audits, and protection of stakeholder interests.

(2) The organization evaluates the performance of leaders at all levels. Senior leaders use these performance reviews to improve both their personal leadership effectiveness and that of leadership system.

b. Legal and Ethical Behavior

(1) The organization addresses and anticipates risks / adverse impacts on society, public concerns with its health care services and operations. The organization prepares for these concerns in a proactive manner, including using resource-sustaining processes. Key processes, measures, and goals for complying with legal requirement and reducing those risks or negative impacts are addressed.

(2) The organization promotes and ensures ethical behavior in all interactions, monitors and responds to breaches of ethical behavior.

c. Support of Communities and Community Health

The organization identifies its key communities and determines areas of emphasis for support. The organization supports and strengthens communities. The organization supports the community health, forms linkages and partnerships with other organization to develop coordinated services and share resources.

I-2 Strategic Management

I – 2.1 Strategy Development

The organization establishes its strategy and strategic objectives to address its challenges and strengthen its performance.

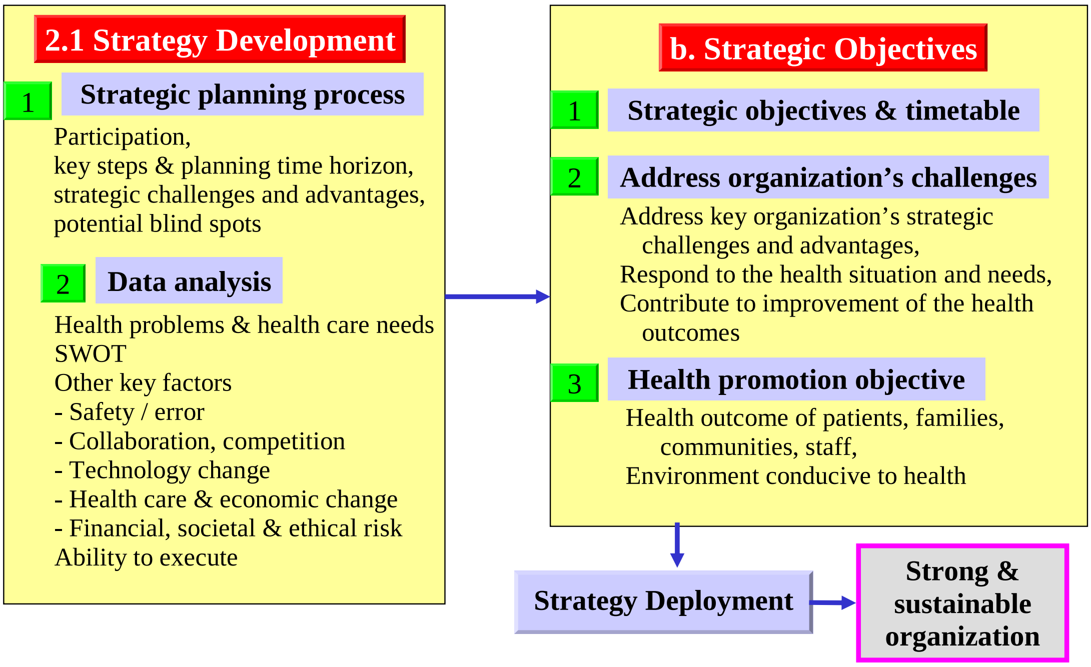

I-2 Strategic Management

2.1 Strategy Development

The organization establishes its strategy and strategic objectives to address its challenges and strengthen its performance.

a. Strategy Development Process

(1) Senior leaders, with participation of staff and key stakeholders, conduct a strategic planning process with appropriate key process steps and planning time horizon. Strategic challenges and advantages are analyzed and determined. The potential blind spots are identified during the planning process.

(2) Health problems and health care needs of the clients / communities; the organization's strengths, weaknesses, opportunities, threats, and other key factors; and the ability to execute the strategic plan are analyzed in the strategic planning process.

b. Strategic Objectives

(1) Key strategic objectives are developed as well as a timetable for accomplishing them. (Show key strategic objectives)

(2) The strategic objectives address key organization's strategic challenges and advantages, respond to the community's (or its served population's) health situation and needs, and contribute to improvement of the health outcomes.

(3) A strategic objective related to health promotion aims at improving health outcome of patients, families, communities, staff, and at environment that is conducive to health.

I – 2.2 Strategy Deployment

The organization converts its strategic objectives into action and tracks progress to ensure goal achievement.

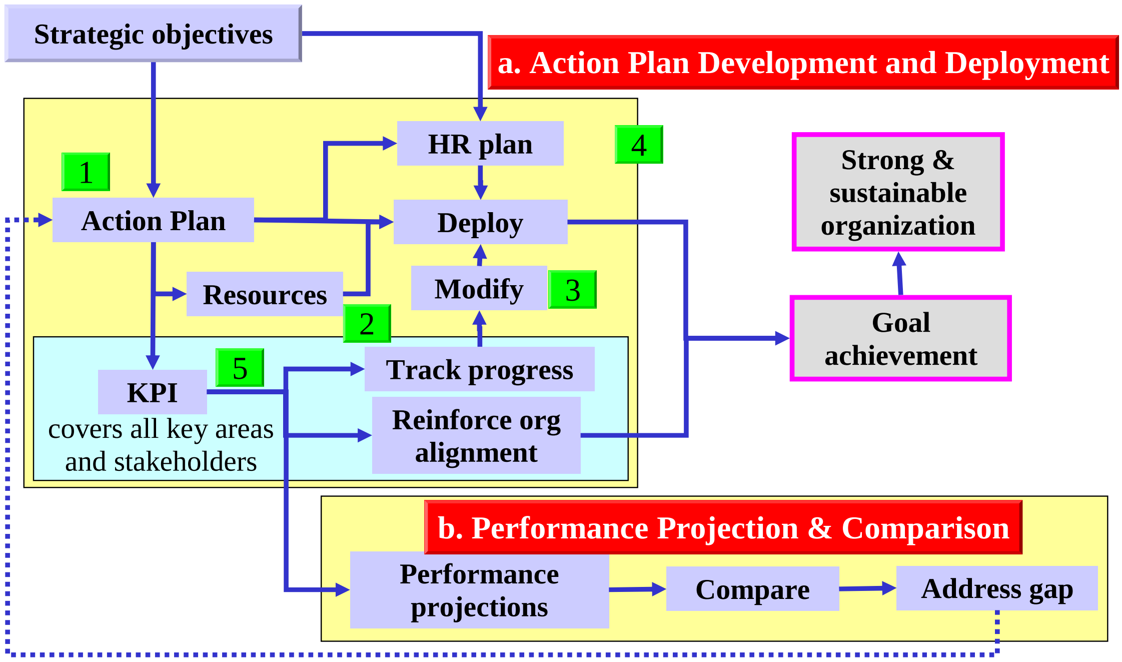

2.2 Strategy Deployment

The organization converts its strategic objectives into action and tracks progress to ensure goal achievement.

a. Action Plan Development and Deployment

(1) Action plans are developed and deployed to achieve the key strategic objectives, ensuring that key outcomes of the action plans can be sustained. Staffs are aware of their role and the contribution they make in achieving the strategic objectives.

(2) The organization ensures that adequate financial and other resources are available to support accomplishment of its action plans. The organization assesses the financial and other risks associated with the plans. The organization balances resources to ensure adequate resource to meet current obligations.

(3) Modified action plans are established and deployed if circumstances require

(4) The organization establishes key human resource plans to accomplish its strategic objectives and action plans. The plans address potential impacts on staff and potential changes to staff capability and capacity needs.

(5) Key performance indicators for tracking progress on the action plans are developed. The measurement system reinforces organizational alignment and covers all key areas and stakeholders.

b. Performance Projection & Comparison

For the key performance indicator identified in 2.2 a (5), the organization determines its performance projections for the planning time horizons, comparing with its goals, past performance, and appropriate comparative data. The organization addresses current and projected gaps in performance against comparable organization.

I-3 Focus on Patients / Customers

I – 3.1 Patient / Customer Knowledge

The organization learns its key patient / customer needs and expectations to ensure the relevance of its services.

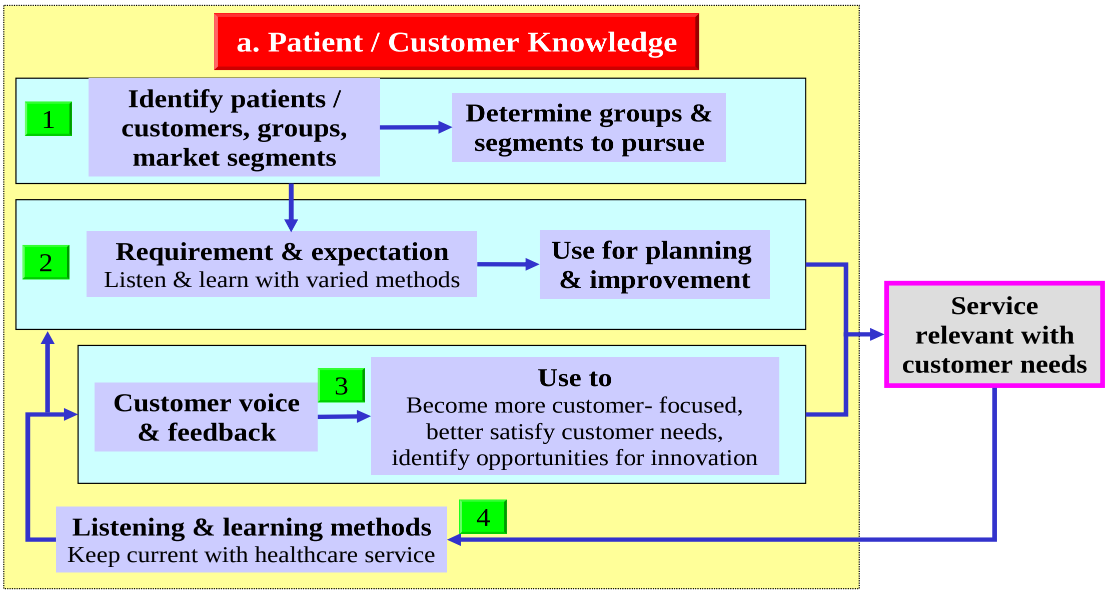

I-3 Focus on Patients / Customers (PCF)

3.1 Patient / Customer Knowledge

The organization learns its key patient / customer needs and expectations to ensure the relevance of its services.

a. Patient / Customer Knowledge

(1) The organization identifies patients / customers, customer groups, and health care market segments. The organization determines which patients / customers, customer groups, and market segments to pursue for health care services.

(2) The organization listens and learns key patient / customer requirements and expectations, and their relative importance to patients' / customers' purchasing decisions. The determination methods vary for different patient / customer groups. This information is used for purposes of health service planning and process improvements.

(3) The organization uses the patient / customer voice and feedback to become more patient- / customer- focused, to better satisfy patient / customer needs, and to identify opportunities for innovation.

(4) The organization keeps its listening and learning methods current with health care service needs and directions.

I – 3.2 Customer Relationship and Satisfaction

The organization builds relationships with patients / customers to promote trust, satisfaction, and cooperation. The organization determines and uses patient / customer satisfaction information for improvement.

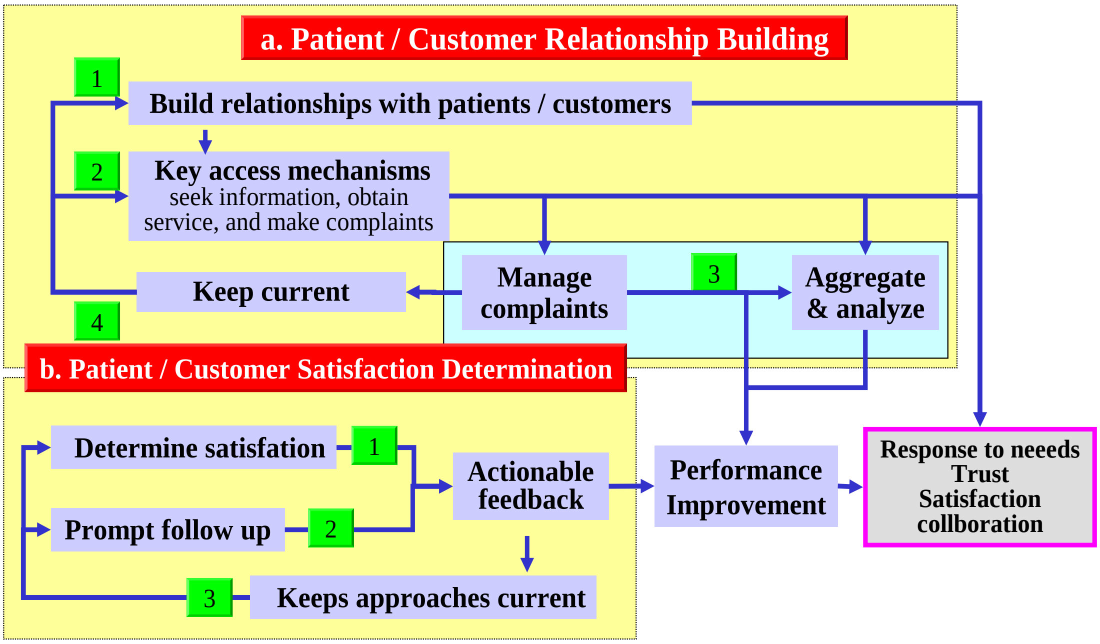

3.2 Customer Relationships and Satisfaction

The organization builds relationships with patients / customers to promote trust, satisfaction, and cooperation. The organization determines and uses patient / customer satisfaction information for improvement.

a. Patient / Customer Relationship Building

(1) The organization builds relationships with patients / customers to meet their expectation, to create trust, and to gain cooperation.

(2) The key access mechanisms enable patients / customers to seek information, obtain service, and make complaints. The organization determines key contact requirements for each mode of patient / customer access and ensures that all these contact requirements are deployed to all people and processes involved in the customer response chain. Accountability to the public for information on the services and their quality is demonstrated.

(3) The organization manages patient / customer complaints, ensuring that complaints are resolved effectively and promptly. Complaints are aggregated and analyzed for use in improvement throughout the organization.

(4) The organization keeps its approaches to building relationships and providing patient and other customer access current with health care service needs and directions.

b. Patient / Customer Satisfaction Determination

(1) Patient / customer satisfaction and dissatisfaction are determined, and this information is used for improvement. The measurement method is appropriate for each patient / customer group, and is able to capture actionable information.

(2) Patients / customers are followed up on care and service quality to receive prompt and actionable feedback.

(3) The organization keeps its approaches to determining satisfaction current with health care service needs and directions.

I – 3.3 Patients' Rights

The organization recognizes and protects the rights of patients.

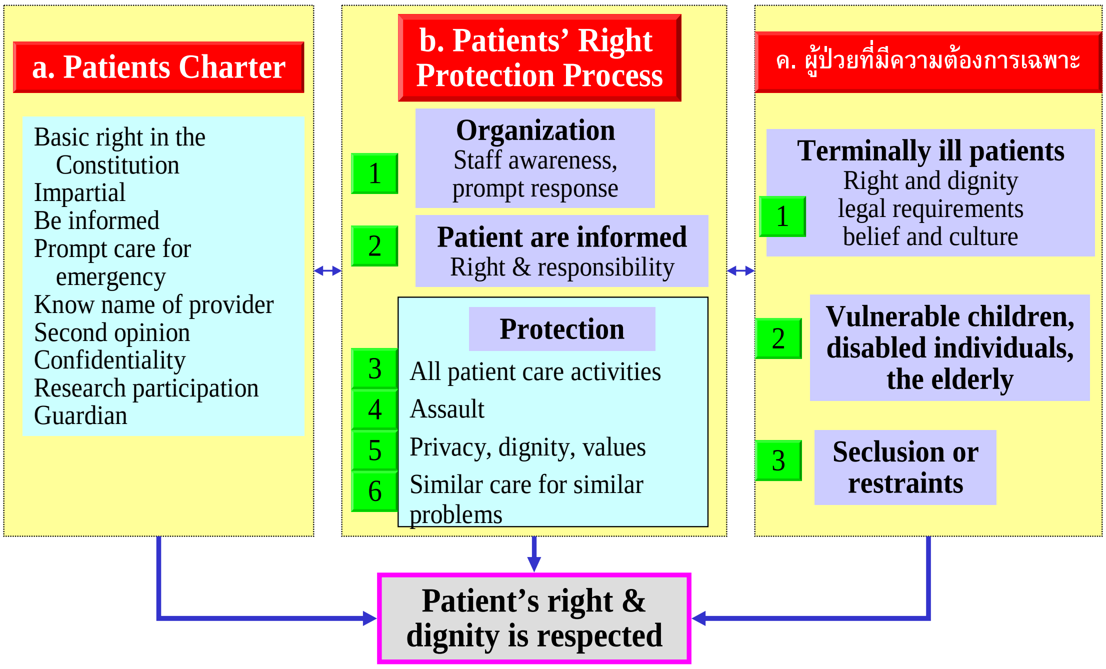

3.3 Patients' Rights

The organization recognizes and protects the rights of patients.

a. Patients Charter

(1) The patients' rights according to the Patient Charter issued by the professional organizations and the Ministry of Health are protected.

b. Patients' Right Protection Process

(1) The organization ensures that staff members are aware of their role in protecting patients' rights. The care system provides prompt response to the request of patients' right.

(2) Patients are informed about their rights and responsibilities in a manner they can understand.

(3) All patient care related activities support and protect patients' rights.

(4) Patients are protected from physical, psychological, and social assault.

(5) The patients' privacy, human dignity, personal values and belief are respected.

(6) The organization ensures that patients with similar problems and severity will receive similar care.

c. Care for Patients with Specific Needs

(1) The terminally ill patients receive care with respect to patient's right and human dignity. The decisions about providing, foregoing, or withdrawing life-sustaining treatment meet legal requirements, belief and culture, and are shared with patients and families.

(2) Vulnerable children, disabled individuals, the elderly, receive appropriate protection.

(3) The patient who needs seclusion or restraints is treated properly.

I-4 Measurement, Analysis and Knowledge Management

I – 4.1 Measurement, Analysis and Improvement of Organizational Performance

The organization measures, analyzes, aligns, reviews, and improve its performance through the use of data and information at all levels and in all parts of the organization.

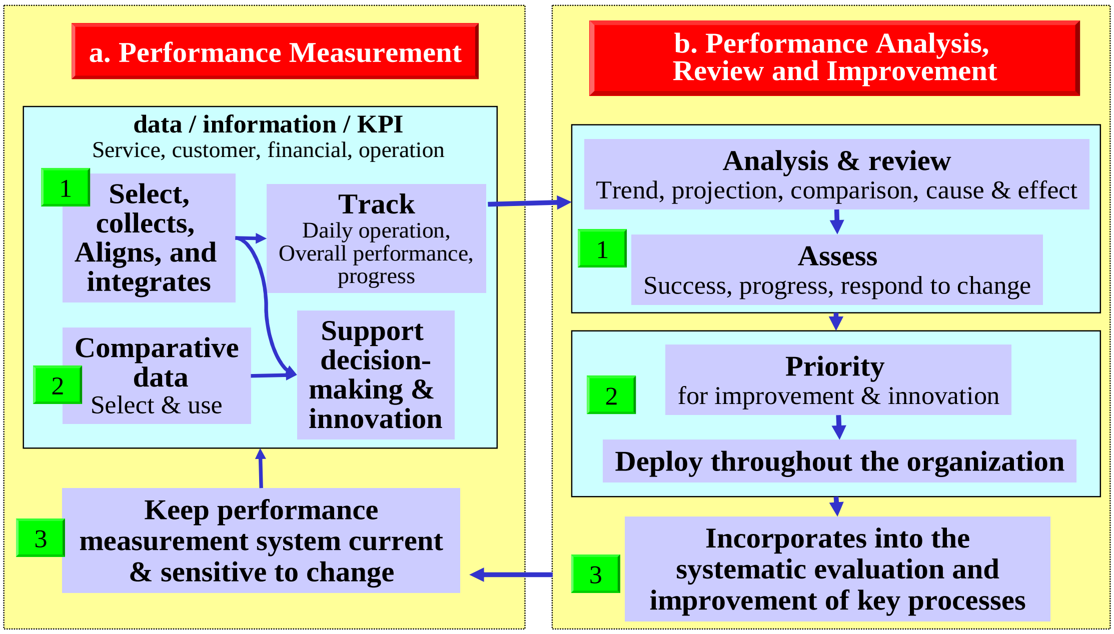

4.1 Measurement, Analysis and Improvement of Organizational Performance

The organization measures, analyzes, aligns, reviews, and improve its performance through the use of data and information at all levels and in all parts of the organization.

a. Performance Measurement

(1) The organization selects, collects, aligns and integrates data / information / key performance indicators (KPI) for tracking daily operations, tracking overall organizational performance, tracking progress relative to strategic objectives and action plans, and supporting organizational decision-making and innovation.

(2) The organization selects and ensures the effective use of key comparative data and information to support decision-making.

(3) The organization keeps its performance measurement system current with health care service needs and directions. The organization ensures that its performance measurement system is sensitive to rapid or unexpected organizational or external changes.

b. Performance Analysis, Review and Improvement

(1) The organization reviews organizational performance and capabilities. The organization performs analyses to support these reviews and to ensure that conclusions are valid. The organization uses these reviews to assess organizational success, progress relative to strategic objectives and action plans, ability to respond to organization and external environment change.

(2) The organization translates organizational performance review findings into priorities for improvement and into opportunities for innovation, of which are deployed throughout the organization.

(3) The organization incorporates the results of organizational performance review into the systematic evaluation and improvement of key processes.

I – 4.2 Management of Information, Information Technology, and Knowledge Management

The organization ensures the quality and availability of needed data, information, software and hardware for staff and patients / customers. The organizational builds and manages its knowledge assets to improve organizational efficiency and effectiveness.

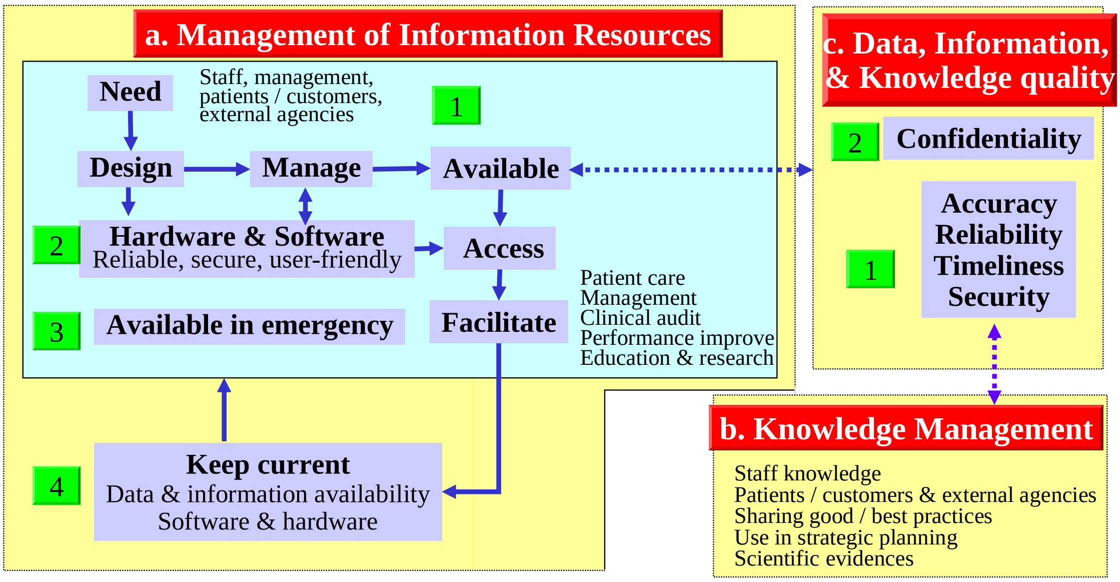

4.2 Management of Information, Information Technology, and Knowledge Management

The organization ensures the quality and availability of needed data, information, software and hardware for staff and patients / customers. The organizational builds and manages its knowledge assets to improve organizational efficiency and effectiveness.

a. Management of Information Resources

(1) Needed data and information are made available to staff, management, patients / customers, and external agencies to facilitate patient care, organization management, clinical audit and performance improvement, education and research. Information plan and management are appropriate to the organization's size and complexity.

(2) The organization ensures that hardware and software are reliable, secure, and user-friendly.

(3) The organization ensures the continued availability of data and information, including the availability of hardware and software systems, in the event of an emergency.

(4) The organization keeps its data and information availability mechanisms, including its software and hardware systems, current with health care service needs and directions and with technological changes.

b. Organizational Knowledge Management

Organizational knowledge is managed to accomplish: the collection and transfer of staff knowledge, the transfer and sharing of relevant knowledge with patients / customers and external agencies, the sharing and implementation of good / best practices, the assembly and transfer of relevant knowledge for use in the strategic planning process, and the adoption of scientific evidences on effectiveness of healthcare intervention.

c. Data, Information, and Knowledge quality

(1) The organization ensures accuracy, reliability, timeliness, and security of its data, information, and organizational knowledge:

(2) The organizational ensures confidentiality of data and information.

I-5 Human Resource Focus

I – 5.1 Staff Engagement

The organization engages, compensates, and motivates its staff to achieve organizational and personal success. Staff and leaders are developed to achieve good performance.

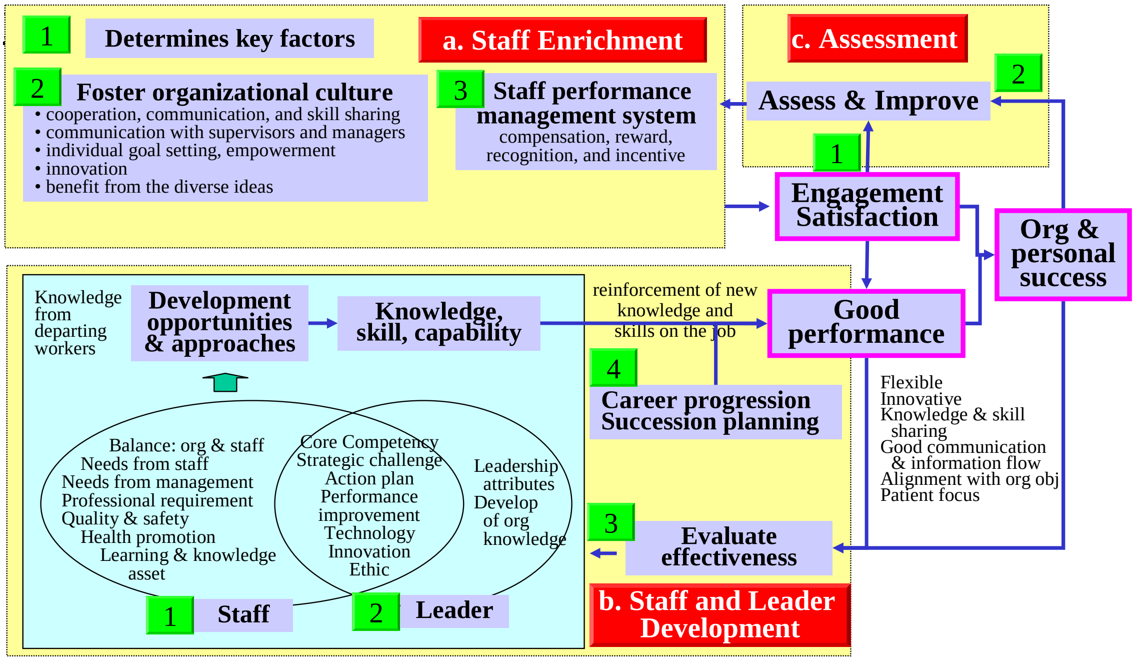

5.1 Staff Engagement

The organization engages, compensates, and motivates its staff to achieve organizational and personal success. Staff and leaders are developed to achieve good performance.

a. Staff Enrichment

(1) The organization determines the key factors that affect staff engagement and satisfaction for different staff groups and segments.

(2) The organization fosters an organizational culture conducive to good performance and a motivated staff to accomplish the following:

- cooperation, effective communication, and skill sharing within and across health care professions, work units, and locations, as appropriate
- effective information flow and two-way communication with supervisors and managers
- individual goal setting, empowerment, and initiative
- innovation in the work environment

• the ability to benefit from the diverse ideas, cultures, and thinking of the staff

(3) The staff performance management system support high performance work and staff engagement, reinforces a patient / customer / health care service focus and achievement of action plans. The staff performance management system considers compensation, reward, recognition, and incentive practices.

b. Staff and Leader Development

(1) The staff development and learning system address the following:

- needs and desires for learning and development identified by the staff, including supervisors and managers
- licensure and re-credentialing requirements
- area of greatest expertise, strategic challenges, and accomplishment of action plans
- ethics, quality, safety, health promotion
- organizational performance improvement, technological change, and innovation
- balance of the organizational objectives and staff needs/desires
- organizational learning and knowledge assets
- the breadth of development opportunities and approaches, including education, training, coaching, mentoring, and work-related experiences, as appropriate
- the transfer of knowledge from departing or retiring workers
- the reinforcement of new knowledge and skills on the job

(2) The development and learning system for leaders address the following:

- development of personal leadership attributes
- development of organizational knowledge
- ethical health care and business practices
- area of greatest expertise, strategic challenges, and accomplishment of action plans
- organizational performance improvement, change, and innovation
- the breadth of leadership development opportunities, including education, training, coaching, mentoring, and work-related experiences, as appropriate

(3) The organization evaluates the effectiveness of its staff and leader development and learning systems, taking into account individual and organizational performance.

(4) The organization manages effective career progression for the entire staff. Effective succession planning for management and leadership positions is accomplished.

c. Assessment of staff engagement

(1) The organization assesses staff engagement and staff satisfaction using formal and informal methods, appropriate with different staff groups and segments, and use these indicators to improve staff engagement.

(2) The organization relates assessment findings to key health care / business results reported in Part IV to identify opportunities for improvement in both staff engagement and health care / business results.

Part I Organization Management Overview

I – 5.2 Staff Environment

The organization manages staff capability and capacity to accomplish the work of the organization. The organization maintains a work environment and a staff support climate that contribute to the health, safety and security.

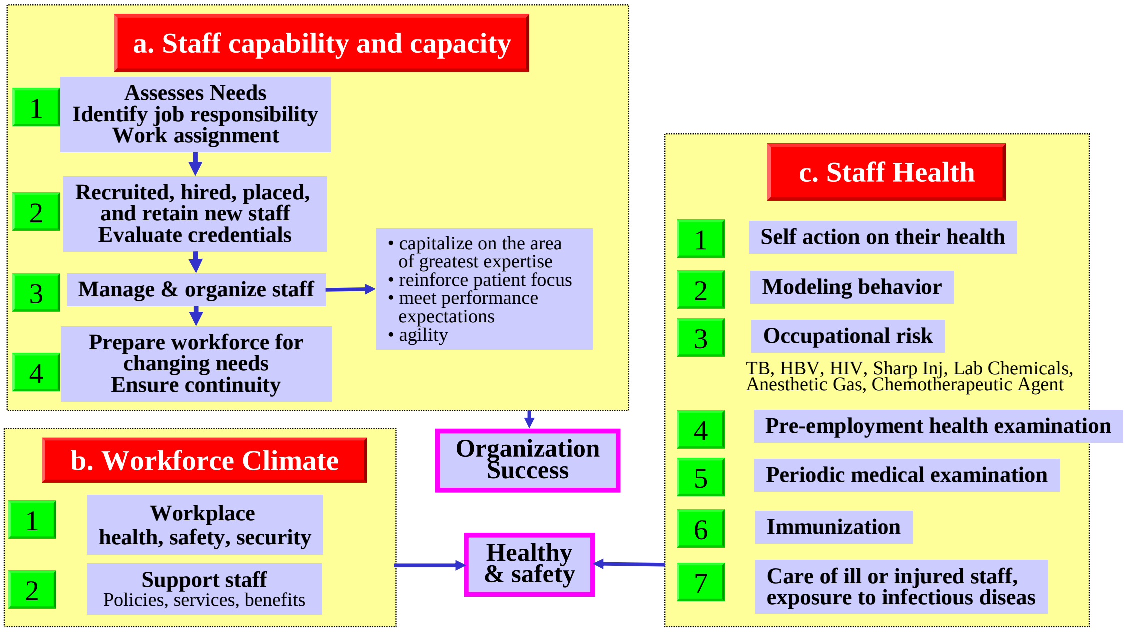

5.2 Staff Environment

The organization manages staff capability and capacity to accomplish the work of the organization. The organization maintains a work environment and a staff support climate that contribute to the health, safety and security.

a. Staff capability and capacity

(1) The organization assesses its staff capability and capacity needs. Job responsibilities are identified and work assignments are based on staff members' credentials and any regulatory requirements.

(2) New staff are effectively recruited, hired, placed, and retained. There is an effective process to gather, verify, and evaluate professional staff members' credentials: license, education, training, and experience. The organization ensures that its staff represents the diverse ideas, cultures, and thinking of its hiring community.

(3) The organization manages and organizes its staff to:

- accomplish the work of your organization
- capitalize on the organization's area of greatest expertise
- reinforce a patient and other customer and health care service focus
- meet or exceed performance expectations
- address its strategic challenges and action plans
- achieve the agility to address changing needs.

(4) The organization prepares its workforce for changing capability and capacity needs. The organization manages its staff, the staff needs and the organization needs to ensure continuity, to prevent staff reductions, and to minimize the impact of staff reductions, if they do become necessary.

b. Workforce Climate

(1) The organization ensures and improves workplace health, safety, and security. The organization determines its performance measures and improvement goals for each of these workplace factors.

(2) The organization supports its staff via policies, services, and benefits, of which are tailored to the needs of a diverse staff and different staff groups and segments.

c. Staff Health

(1) The staff involve, learn, decide and take action on enhancing their health, both physical, mental, and social health.

(2) The staff come to the agreement on modeling behavior of individual staff and organization culture for health promotion.

(3) The organization assesses and manages, systematically and continuously, major occupational risks for healthcare personnel especially TB, HBV, HIV transmission from patient, needle stick injuries, laboratory chemicals, anesthetic gases and chemotherapeutic agents.

(4) All staff have a pre-employment health examination which includes at least the following information, baseline health examination, general health assessment, immunization status, illness, accident, and health behavior. In particular, the staffs should have job specific health assessment.

(5) Appropriate staff have periodic medical evaluations to assess for work-related illness and infectious diseases that may have an impact on patient care and other staff.

(6) Staff are offered appropriate immunizations for communicable diseases.

(7) Ill or injured staff receive appropriate evaluation and care. Policies and procedures are developed for assessment of disease communicability, indications for work restrictions, and management of employees who have been exposed to infectious diseases, including post exposure prophylaxis and work restrictions.

Part I Organization Management Overview

I-6 Process Management

I – 6.1 Work Systems Design

The organization determines its area of greatest expertise and designs its work systems and key processes to deliver value to patients / other customers, prepare for potential emergencies, and achieve organizational success.

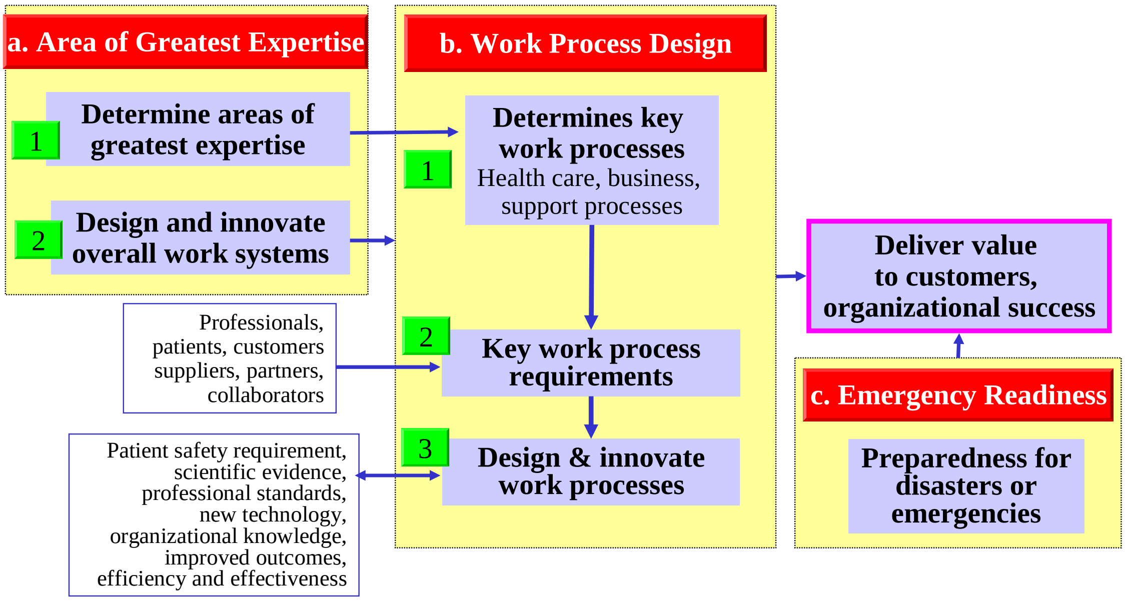

6.1 Work Systems Design

The organization determines its area of greatest expertise and designs its work systems and key processes to deliver value to patients / other customers, prepare for potential emergencies, and achieve organizational success.

a. Area of Greatest Expertise

(1) The organization determines its areas of greatest expertise, of which relate to its mission, environment, and action plans.

(2) The organization designs and innovates its overall work systems with appropriate use of external resources.

b. Work Process Design

(1) The organization determines its key work processes, of which relate to its area of greatest expertise. These processes contribute to delivering value to patients and other customers, and organizational success. (Show the key work processes)

(2) The organization determines its key work process requirements, incorporating input from professionals, patients, other customers, suppliers, partners, and collaborators. (Show the key process requirements)

(3) The organization designs and innovates its work processes to meet all the key requirements, incorporating patient safety requirement, scientific evidence and professional standards, improved outcome, new technology, organizational knowledge, the potential need for agility, improved health care outcomes, cycle time, productivity, efficiency and effectiveness factors into the design of these processes.

c. Emergency Readiness

The organization ensures work system and workplace preparedness for disasters or emergencies, considering prevention, management, continuity of operations for patients and the community, evacuation, and recovery.

I – 6.2 Work Process Management and Improvement

The organization implements, manages, and improves its key work processes to deliver value to patients / other customers and achieve organizational success.

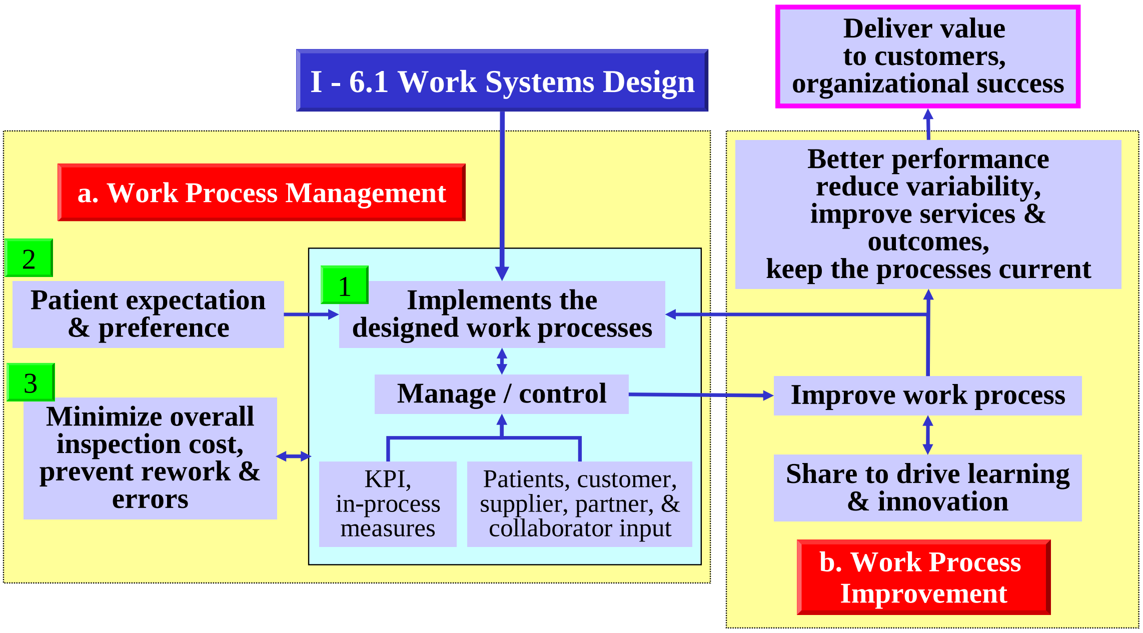

6.2 Work Process Management and Improvement:

The organization implements, manages, and improves its key work processes to deliver value to patients / other customers and achieve organizational success.

a. Work Process Management

(1) The organization implements its work processes and ensures they meet the key process requirements. Patients, other customer, supplier, partner, and collaborator input are used in managing these processes. The organization uses key performance indicators and in-process measures for the control and improvement of its work processes.

(2) Patients' expectations are considered. Health care service delivery processes and likely outcomes are explained to set realistic patient expectations. Patient decision-making and patient preferences are factored into the delivery of health care services.

(3) The organization minimizes overall costs associated with inspections and audits. Rework and errors are prevented.

b. Work Process Improvement

The organization improves its work processes to achieve better performance, to reduce variability, to improve health care services and health care outcomes, and to keep the processes current with health care needs and directions. The improvements and lessons learned are shared with other organizational units and processes to drive organizational learning and innovation.

PART II KEY HOSPITAL SYSTEMS

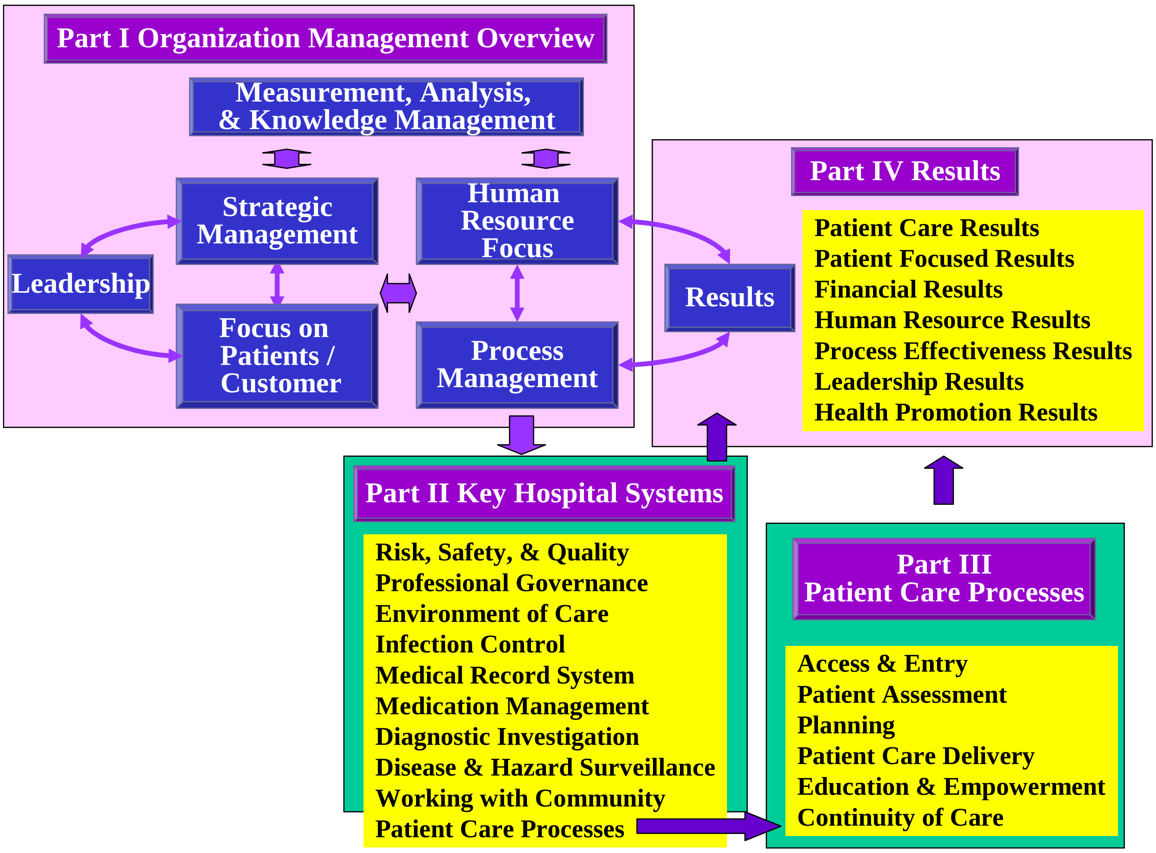

II-1 Risk, Safety, and Quality Management

II – 1.1 Quality Improvement Overview

There is a concerted and coordinated effort for quality program at all levels.

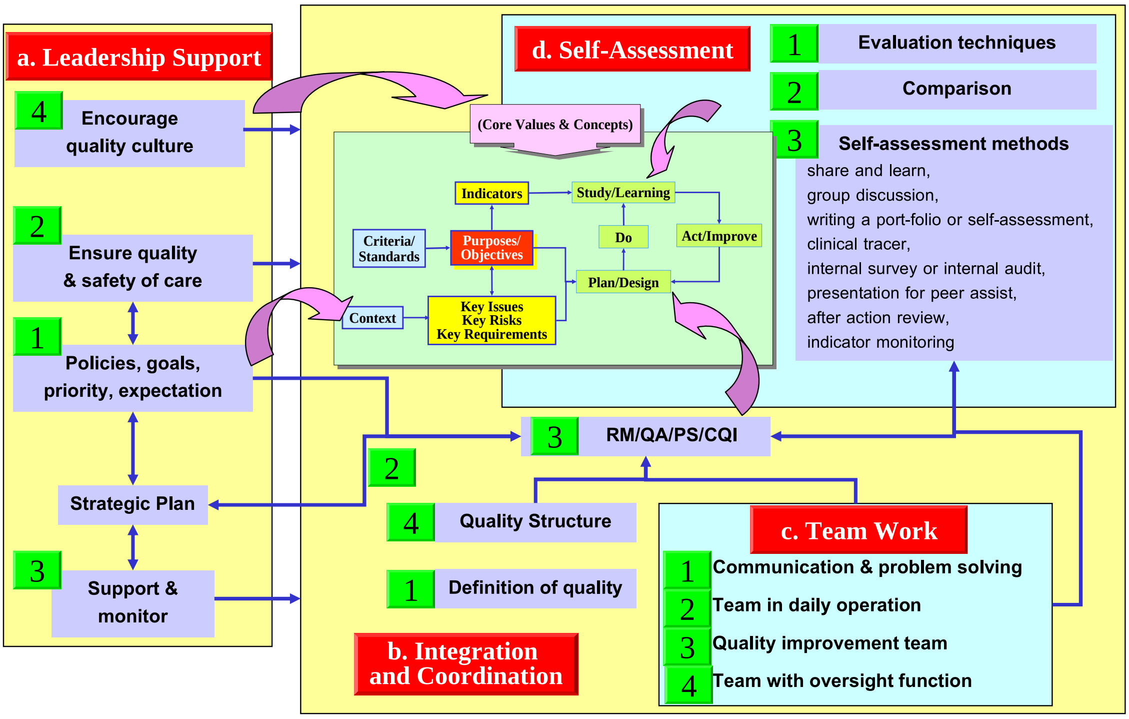

Part II Key Hospital Systems

1.1 Quality Improvement Overview

There is a concerted and coordinated effort for quality program at all levels.

a. Leadership Support

(1) Senior leaders set policies, goals, priorities and expectation for quality and safety.

(2) Senior leaders ensure the safety and quality of care provided.

(3) Leadership at all levels support and monitor the safety and quality improvement efforts.

(4) Senior leaders encourage culture of customer responsiveness, continuous improvement, and learning.

b. Integration and Coordination

(1) The operating definitions for “risk” and “quality” of the organization are determined.

(2) The risk, safety, and quality management program is a part of and aligns with the organization’s strategic plan.

(3) The risk management program, quality assurance program, patient safety program, and continuous quality improvement program are integrated and coordinated at all steps of planning, implementation and evaluation.

(4) An effective quality structure appropriate to the organization has been established up to facilitate and coordinate the program. Accountability and responsibilities for quality and performance improvement are defined.

c. Team Work

(1) There are effective communication and problem solving within work units, between work units, between professions, between staff and management, and between staff and patient / customer.

(2) The staffs collaboratively provide high quality care and service with safety conscious and professional responsibility (team work in daily operation).

(3) Varieties of quality improvement teams are encouraged: both self-directed team and commissioned team, both single unit team and cross-functional or multidisciplinary team, both clinical and non-clinical team (quality improvement team).

(4) There are cross-functional or multidisciplinary oversight mechanisms to give direction, support and monitor quality and safety initiatives, e.g. clinical lead team, system specific team (team with oversight function).

d. Self-Assessment

(1) A spectrum of evaluation techniques is used appropriately to identify opportunity for improvement, i.e. from a qualitative method to a systematic quantitative method or using research methodology.

(2) The comparison with patient / customer requirement, the hospital standards and other standard requirement, goals and objectives of the organization and work units, national or international benchmarks are used as appropriate.

(3) Varieties of self-assessment methods are used, i.e. share and learn, group discussion, writing a port-folio or self-assessment form, clinical tracer, internal survey or internal audit, presentation for peer assist, after action review, indicator monitoring.

Part II Key Hospital Systems

II – 1.2 Risk, Safety, and Quality Management System

There is an effective and coordinated hospital risk, safety, and quality management system, including integrated approach for patient care quality improvement.

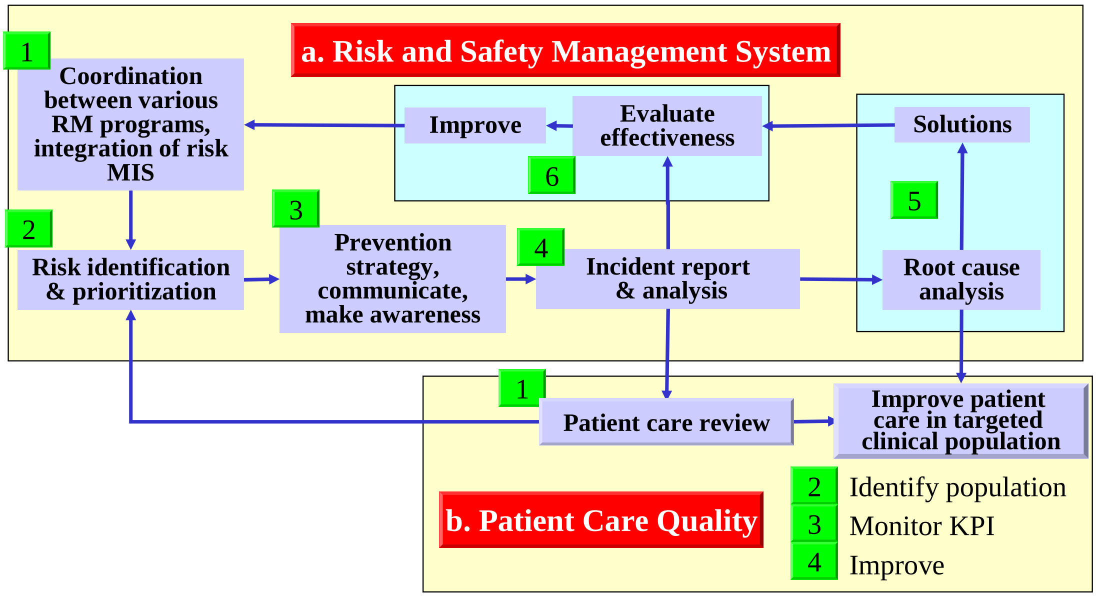

1.2 Risk, Safety, and Quality Management System

There is an effective and coordinated hospital risk, safety, and quality management system, including integrated approach for patient care quality improvement.

a. Risk and Safety Management System

(1) There is an effective coordination and collaboration between various risk management programs, including integration of risk management information system.

(2) Clinical and non-clinical risks are identified and prioritized at all work units and at all levels to determine safety goals and prevention strategies.

(3) Prevention strategies and measures are carefully designed, thoroughly communicated and made awareness for effective implementation.

(4) An effective and appropriate incidence reporting system, including near miss event, is established. Data is analyzed and used for evaluation, improvement, learning and planning.

(5) Root cause analysis is used to identify the underlying system factors contributed to the adverse events and appropriate solutions are implemented accordingly.

(6) The effectiveness of the hospital risk and safety management program is evaluated regularly and used for improvement.

b. Patient Care Quality

(1) Patient care and service are regularly reviewed to evaluate the quality and efficiency of care and identify opportunity for improvement.

(2) The healthcare teams identify clinical populations as targets for improvement, as well as goals and objectives of patient care and improvement.

(3) The healthcare teams use appropriate indicators to monitor performance of patient care for the identified population.

(4) The healthcare teams use a concerted action and varieties of methods to improve patient care, e.g. multidisciplinary approach, holistic approach, evidence-base approach, root cause analysis, innovation and benchmarking. The improvement includes dimension of prevention, promotion, curative and rehabilitation, as appropriate.

II-2 Professional Governance

II – 2.1 Nursing Governance

There is an organized nursing administration responsible for high quality nursing service to fulfill the mission of the organization.

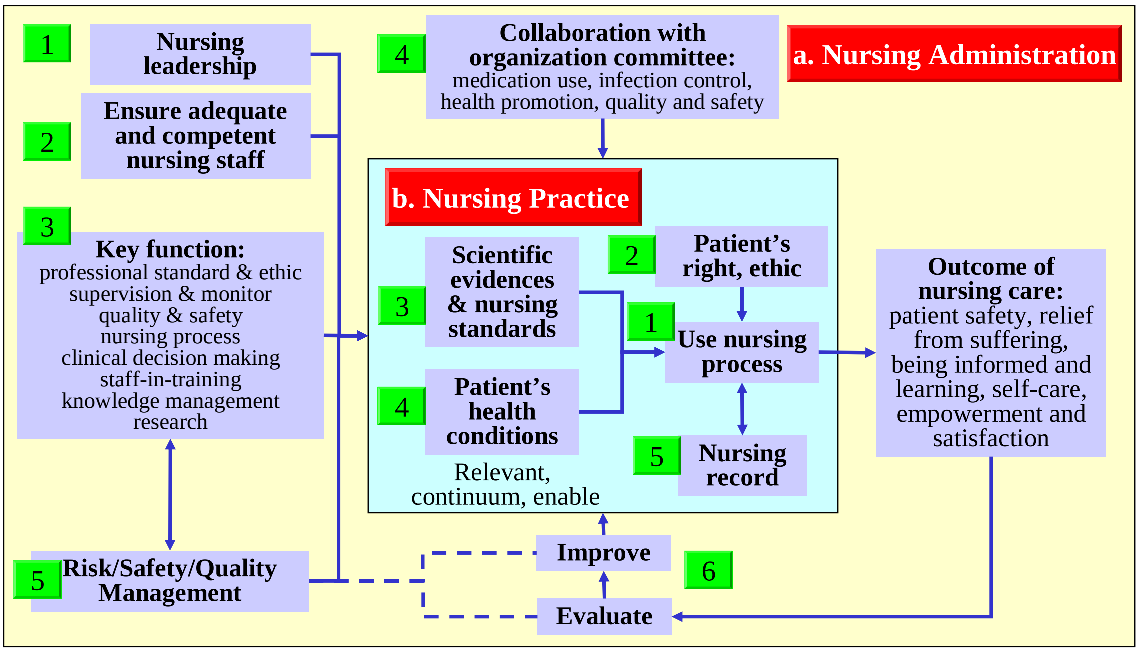

Part II Key Hospital Systems

2.1 Nursing

There is an organized nursing administration responsible for high quality nursing service to fulfill the mission of the organization.

a. Nursing Administration

(1) Nursing leadership at all level is responsible by qualified registered nurses with experience on both nursing practice and nursing administration.

(2) The nursing administration ensures adequate and competent nursing staff for the service provided.

(3) The nursing administration effectively carries out these key functions:

-oversight of professional standards and ethics

-supervision, monitoring, and encouraging improvement in quality and safety for patient care

-encouraging the use of nursing process

-support appropriate clinical decision making and use of technology

-supervision of nursing staff-in-training

-knowledge management and research for professional development

(4) The nursing administration work collaboratively with the organization committee on medication use, infection control, health promotion, quality and safety.

(5) The risk, safety, and quality management in nursing care aligns with and supports the organization goals and objectives, professional standards and ethics.

(6) The goals of patient safety, relief from suffering, being informed and learning, self-care, empowerment and satisfaction are used for evaluation and improvement of nursing care.

b. Nursing Practice

(1) The nursing staffs, in collaboration with other professionals, use nursing processes to provide high quality, comprehensive and holistic nursing care to individuals, families, and communities.

(2) The nursing staffs provide nursing care with respect to the patient's right and professional ethics.

(3) The nursing staffs provide nursing care on the basis of updated scientific evidence and nursing standards, with systematic monitoring, evaluation and continuous improvement.

(4) The nursing staffs provide a continuum of care according to the patient's health condition, lifestyle and social context, plan for continuum of care with healthcare team and clients / families from entry to after discharge, to enable patients and families for self-care and control of risk factors, and use resources for appropriate self-care.

(5) The nursing record reflects the holistic and continuum of nursing care and is useful for communication, continuum of care, evaluation of nursing care quality, and research.

II – 2.2 Medical Staff Governance

There is an organized medical staff organization, responsible for supporting and oversight of standard and ethical practice of medical professional to fulfill the mission of the organization.

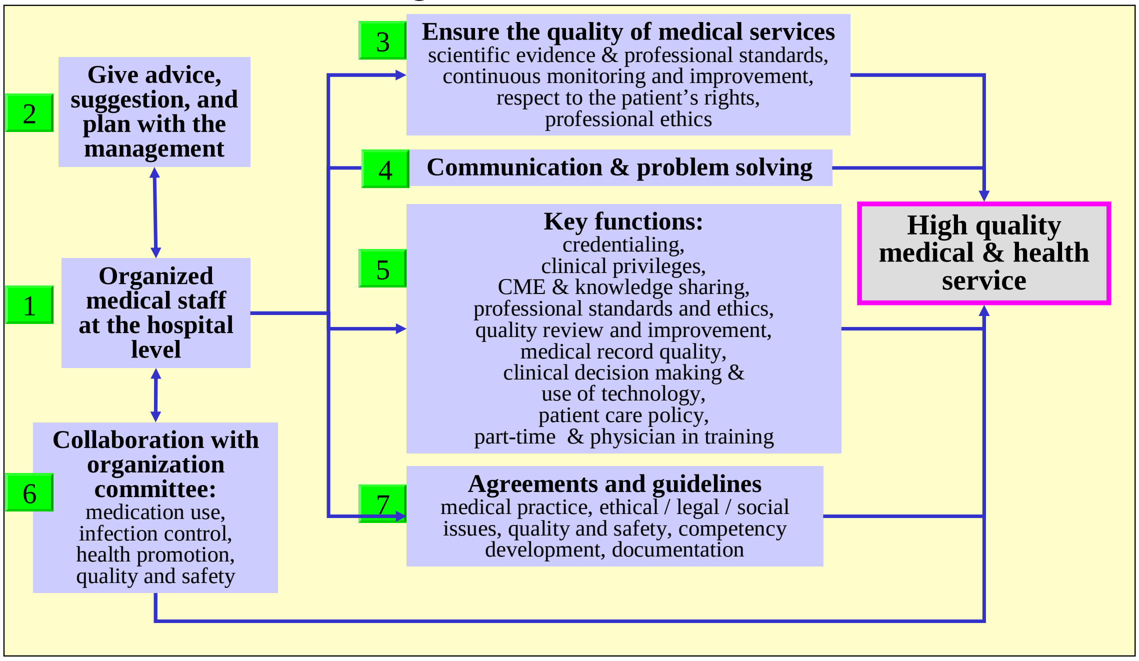

2.2 Medical Staff

There is an organized medical staff organization, responsible for supporting and oversight of standard and ethical practice of medical professional to fulfill the mission of the organization.

(1) The medical staffs are organized at the hospital level to ensure high quality professional practices and accountability.

(2) The medical staff organization gives advice, suggestion, and collaboratively plans with the management on the provision of high quality medical and public health services.

(3) The medical staff organization ensures the provision of medical services based on scientific evidence and professional standards, with continuous monitoring and improvement of quality, and respect to the patient’s rights and professional ethics.

(4) Structure and mechanism to support communication and problem solving within the medical professional, between physicians and other professionals or other units, between physician and clients is established.

(5) The medical staff organization effectively carries out these key functions:

-credentialing
-granting of clinical privileges
-continuing medical education and knowledge sharing
-oversight of professional standards and ethics
-oversight of patient care quality review and improvement
-ensure medical record quality
-support appropriate clinical decision making and use of technology
-patient care policy development or endorsement
-supervision of physician in training and part-time on call physician

(6) The medical staff organization work collaboratively with the organization committee on medication use, infection control, health promotion, quality and safety.

(7) Agreements and guidelines for physician are established and followed, including medical practice, ethical / legal / social issues, quality and safety, competency development, documentation.

II-3 Environment of Care

II – 3.1 Physical Environment and Safety

The organization's physical environment contributes to the safety and well-being of patients, staff, and visitors. The organization ensures that all occupants are safe from fire, hazardous material and waste, or other emergencies in the facilities.

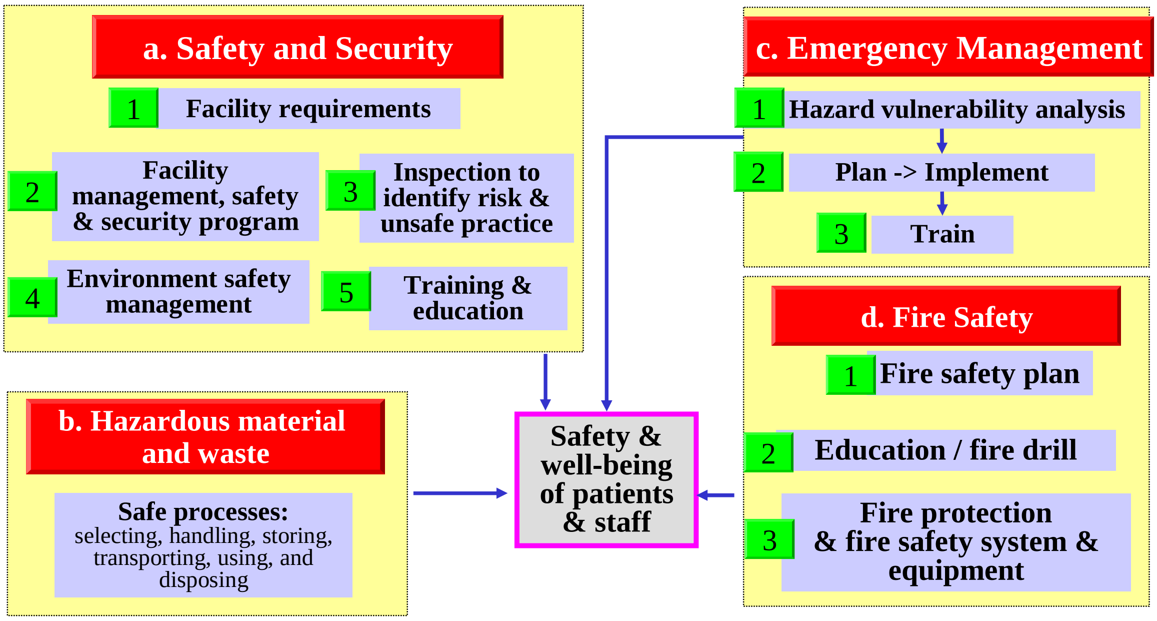

3.1 Physical Environment and Safety

The organization's physical environment contributes to the safety and well-being of patients, staff, and visitors. The organization ensures that all occupants are safe from fire, hazardous material and waste, or other emergencies in the facilities.

a. Safety and Security

(1) The organization complies with relevant laws, regulations, and facility inspection requirement. The design and layout of the buildings ensure a safe, comfortable, patient privacy and work effective environment.

(2) The facility management, safety and security program oversight is assigned to one or more individual. All aspects of the program are monitored and improved.

(3) The organization conducts facility and environment inspection to identify all environmental risks and unsafe practices at least every six months in all areas where patients / visitors are served and at least annually in other areas.

(4) The organization conducts a proactive risk assessment, develops and implements a safety management plan to reduce the identified risk, to prevent injury, and to maintain clean and safe conditions for patients / visitors and staff.

(5) All staff members are educated and trained about their roles in providing a safe and effective patient care environment.

b. Hazardous material and waste

(1) The organization safely manages its hazardous materials and waste, identifies and implements processes for selecting, handling, storing, transporting, using, and disposing of hazardous material and waste.

c. Emergency Management

(1) The organization conducts a hazard vulnerability analysis to identify potential emergency that could affect the need for its services.

(2) The organization develops an emergency management plan describing the process for disaster readiness and emergency management, and implements it when appropriate.

(3) The organization conduct drills regularly to test emergency management.

d. Fire Safety

(1) The organization develops and implements a fire safety plan. The fire safety plan includes fire prevention / risk reduction, early detection, suppression, abatement, and safe exit from fire, or other emergencies in the facility.

(2) The organization conducts hospital wide safety education programs to promote awareness and conducts fire drills regularly. Deficiency and opportunities for improvement is identified, readiness of fire equipment is evaluated, effectiveness of fire response training and staff knowledge is evaluated from the fire drill.

(3) The organization regularly inspects, tests and maintains fire protection and fire safety systems and equipment.

Part II Key Hospital Systems

II – 3.2 Equipment and Utility System

The organization ensures that essential equipment is available for use and functions properly and ensures continuity of essential utility services.

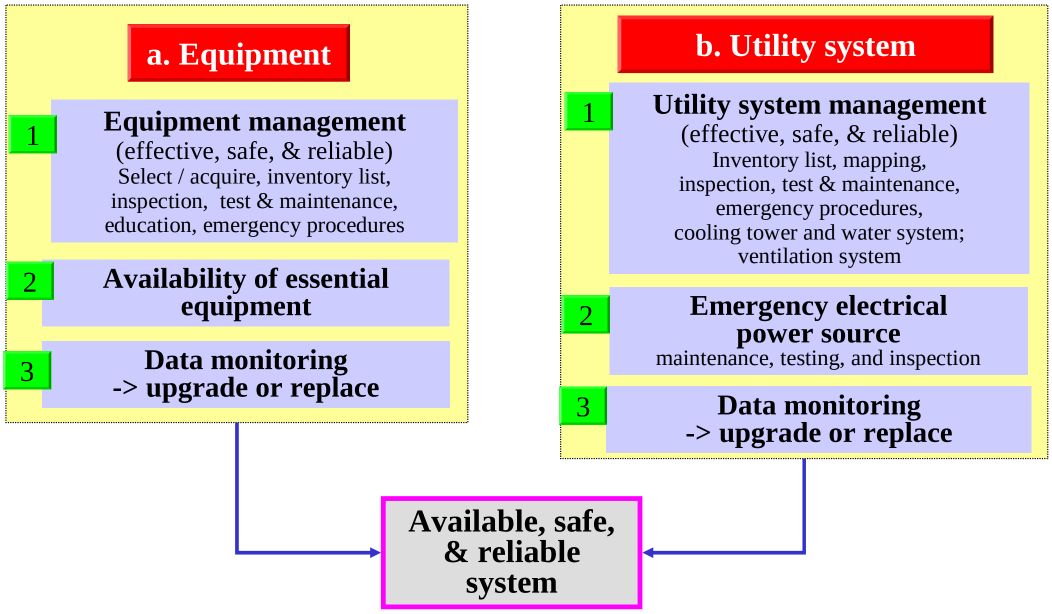

3.2 Equipment and Utility System

The organization ensures that essential equipment is available for use and functions properly and ensures continuity of essential utility services.

a. Equipment

(1) The organization develops and implements an equipment management plan for effective, safe, and reliable operation of equipment. The plan includes a process for selecting and acquiring equipment; creating an inventory of equipment to be included in the equipment management plan; performance and safety testing of equipment before initial use; appropriate inspection, test and maintenance strategies at defined interval; users' education, and emergency procedures with equipment.

(2) Essential medical equipment is available and ready for safe patient care.

(3) The organization collects monitoring data for equipment management program. These data are used to plan for long term upgrading or replacing equipment.

b. Utility system

(1) The organization develops and implements a management plan for effective, safe, and reliable operation of utility systems. The plan includes creating an inventory of operating component of systems; mapping the distribution of utility system; appropriate inspection, test and maintenance strategies at defined interval; and emergency procedures for utility systems disruption; minimizing pathogenic biological agents in cooling tower and water system; efficiency of ventilation system to control airborne contaminants.

(2) The organization provides an emergency electrical power source to all critical service areas with appropriate and regular maintenance, testing, and inspection.

(3) The organization collects monitoring data for the utility management program and used to plan for upgrading or replacing the utility system.

II – 3.3 Environment for Health Promotion and Environment Protection

The organization demonstrates its commitment for the hospital to be a healthy and safe workplace, to support health promotion activities, and to protect the environment.

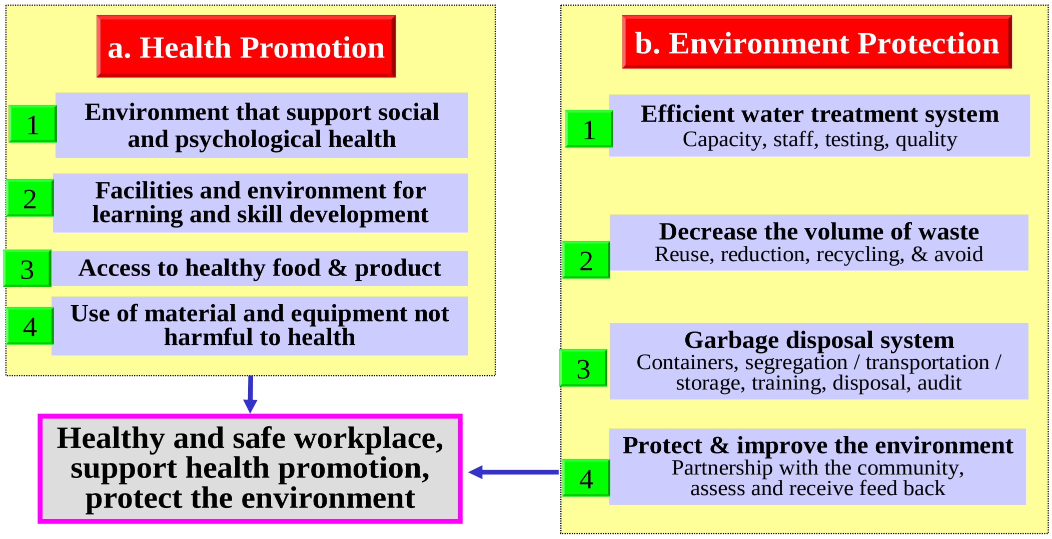

3.3 Environment for Health Promotion and Environment Protection

The organization demonstrates its commitment for the hospital to be a healthy and safe workplace, to support health promotion activities, and to protect the environment.

a. Health Promotion

(1) The organization establishes environment that support social and psychological health of patients, families, and staff.

(2) The organization establishes facilities and environment for learning and skill development of staff, patients and other customers, and general public.

(3) The organization promotes access to and consumption of appropriate healthy food and product.

(4) The organization promotes the use of material and equipment which are not harmful to health.

b. Environment Protection

(1) There is an efficient water treatment system:

-the system has capacity that match amount of waste water

-the system is taken care by trained staff

-treated water is tested according to the rules and regulations

-quality of treated water at the time of peak load complies with the standards

(2) The organization manages to decrease the volume of waste through a program of reuse, reduction, recycling and avoid material that is harmful to the environment.

(3) The garbage disposal system conforms with sanitation requirements.

-proper and adequate containers

-proper segregation / transportation / storage of general, infectious and hazardous waste

-training of staff for proper waste transportation and disposal

-proper processes for disposal of infectious and hazardous waste

-audit of the infectious waste disposal by contractors

(4) The organization, in partnership with the community and other organizations, protects and improves the environment. The organization assesses and receives feed back on hospital waste management that may have an impact on the communities.

II-4 Infection Control and Prevention

II – 4 Infection Prevention and Control

4.3 The organization performs appropriate methods of surveillance and monitoring to detect and control infections, and manage nosocomial outbreak situations.

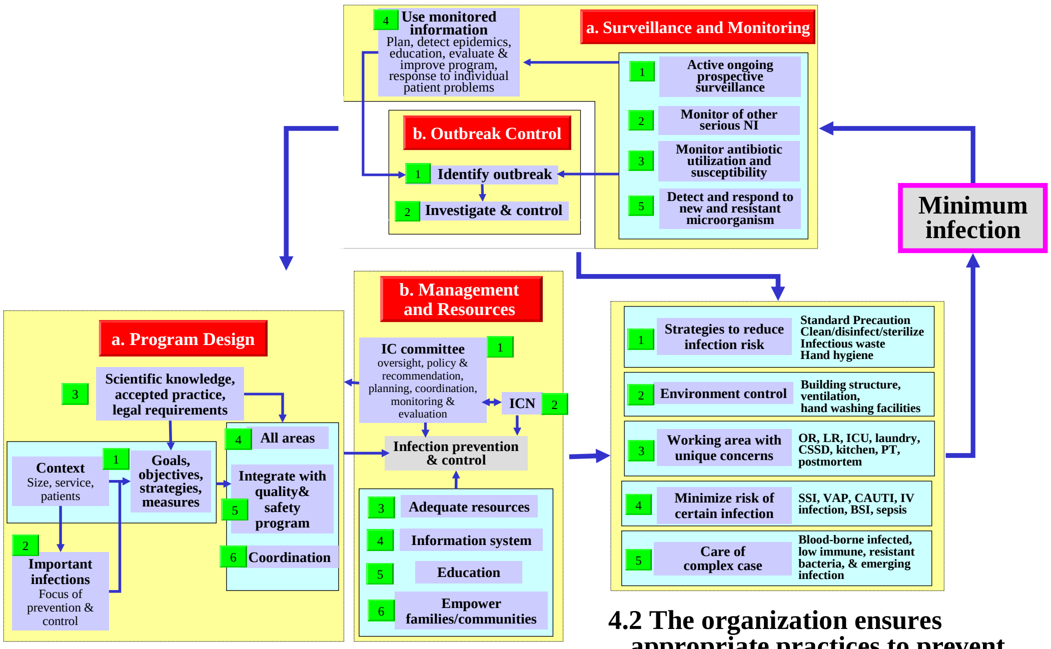

4.1 The organization's infection prevention and control program is appropriately designed, adequately supported, and well coordinated.

4.2 The organization ensures appropriate practices to prevent nosocomial infection.

4.1 Infection Prevention and Control Program

The organization's infection prevention and control program is appropriately designed, adequately supported, and well coordinated.

a. Program Design

(1) The goals, objectives, strategies and measures of the infection control program appropriate to the organization's size, service and patients are established.

(2) Epidemiological important infections, infectious site and associated devices that will provide the focus of the nosocomial infection prevention and control are identified.

(3) The infection control program is based on current scientific knowledge, accepted practice, meets legal requirements and is documented in policies and procedures.

(4) All areas of the organization that serve patients, staff and visitor are included in the infection control program.

(5) The infection control process is integrated with the organization's overall program for quality improvement and safety.

(6) The processes for preventing and controlling infections are coordinated and consistently carried out by all staff across the organization and the patients' homes, if applicable.

b. Management and Resources

(1) A designated individual or committee, as appropriate to the organization, is responsible for oversight of the program, policy and recommendation development, planning, coordination, monitoring and evaluation.

(2) One or more individuals (ICN), as appropriate to the number of hospital beds, is / are responsible for implementing the infection prevention and control program. This individual(s) is qualified in infection control practices through education, training, or experience, and has a clear role definition. The ICN(s) have written authority to institute infection control measures or studies when there is a perceived danger to the patients or hospital staffs.

(3) There are adequate resources to prevent and control infections.

(4) The organization information systems support the infection control program.

(5) Staff members are educated on an ongoing basis about the risks of infection, the organization policies, and their role in preventing infections.

(6) Activities that educate and empower the family / community how to reduce the risk of infection and prevent infections from spreading in the household / community are established.

4.2 Infection Prevention

The organization ensures appropriate practices to prevent nosocomial infection.

a. Infection Prevention

(1) Procedures and processes associated with the risk of infection are identified and strategies implemented to reduce infection risk:
- -standard precautions and isolation precautions
- -cleaning, disinfection, and sterilization
- -handling, storing, and disposing of infectious material
- -promoting hand washing and personal hygiene

(2) The organization establishes an environment control to minimize the risk of infection transmission and contamination of the environment:
- -building structure, ventilation, and maintenance to prevent the spread of contaminants and infection
- -physical setting and facilities for hand washing, cleaning, and separation of clean and dirty utility area

(3) Working areas with unique infection control concerns are identified and strategies implemented to reduce infection risk, especially in these area:
- -operating room
- -labor room
- -ICU / critical care unit
- -laundry
- -CSSD
- -kitchen
- -physical therapy
- -postmortem area

(4) Programs are implemented to minimize risk of certain important infection of the organization, e.g. surgical site infection, respiratory tract infection, urinary tract infection, IV infection and bloodstream infection.

(5) There are policies and procedures for dealing with blood-borne infected patients, low immune patients, resistant bacteria, and emerging infection.

4.3 Surveillance, Monitoring, and Outbreak Control

The organization performs appropriate methods of surveillance and monitoring to detect and control infections, and manage nosocomial outbreak situations.

a. Surveillance and Monitoring

(1) There is ongoing surveillance of nosocomial infections that is appropriate to the organization. The organization proactively monitors and tracks risks, rates and trends in nosocomial infection.

(2) Patient areas in which active prospective surveillance in (1) is not conducted are monitored for the occurrence of serious nosocomial infection.

(3) Antibiotic utilization and susceptibility (if possible) are monitored and communicated to appropriate individuals and committees.

(4) The monitored information is used for planning, detecting epidemics, directing education, evaluation and improvement of the program, including response to individual patient problems.

(5) The organization works with government agencies, other organizations and the community to promptly detect and respond to the presence of new and resistant microorganism.

b. Outbreak Control

(1) Occurrence of outbreaks or clusters of infectious diseases are identified by analysis of surveillance data, maintaining regular contact with clinical staff, regularly reviewing microbiology record to identify unusual clusters or a greater-than-usual incidence of certain species or strains of microorganisms (if possible).

(2) When an outbreak occurs, the infection control team has adequate resources and authority to ensure a comprehensive and timely investigation and the implementation of appropriate control measures.

II-5 Medical Record System

II – 5.1 Record Management System

The organization establishes an efficient medical record management system to serve the needs of all parties.

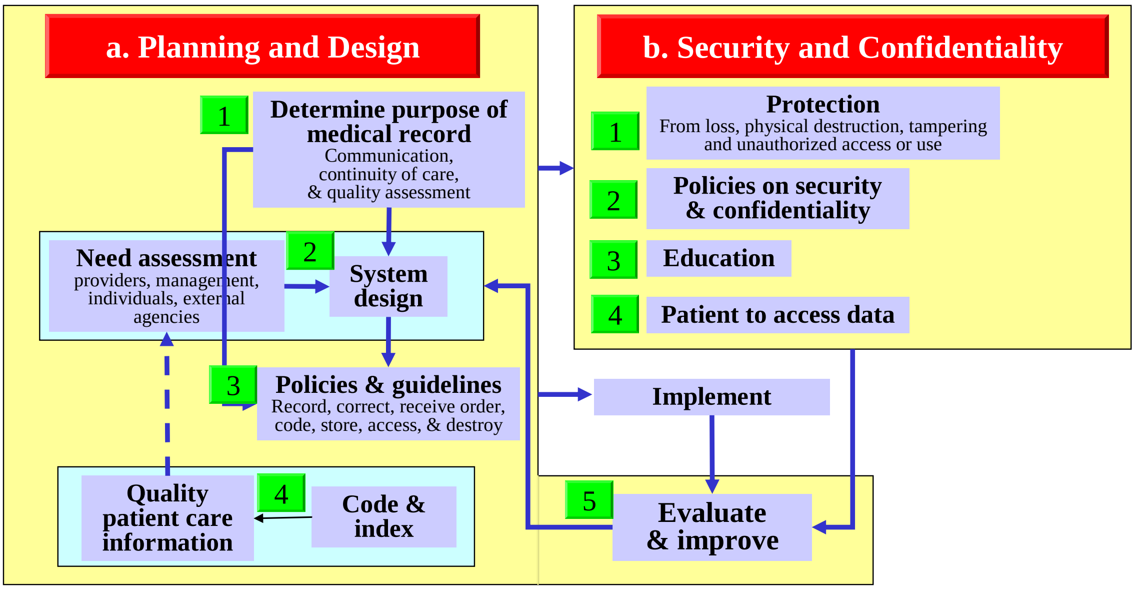

5.1 Record Management System

The organization establishes an efficient medical record management system to serve the needs of all parties.

a. Planning and Design

(1) The purposes of medical record are collaboratively determined by all relevant health professionals. The purposes cover communication, continuity of care, and quality assessment.

(2) The design of the medical record system is based on the assessment of the needs of the care providers, management, including individuals and agencies outside the organization.

(3) The organization has appropriate policies and guidelines for patient's medical record, including recording, correcting, order receiving, using standard code, storing, providing access, and destroying.

(4) Medical record data are coded and indexed to ensure the timely production of quality patient care information.

(5) The organization regularly evaluates and improves medical record management system to ensure that it meets the need of the organization and its patients.

b. Security and Confidentiality

(1) Records are protected from loss, physical destruction, tampering and unauthorized access or use.

(2 The organization defines essential policies and guidelines to maintain the security and confidentiality of patient's data and information in the medical records.

(3) The organization continually educates staffs about their responsibilities regarding confidentiality, how to handle situations where requests for release of information would violate confidentiality.

(4) The organization has a process for patients to access their data and information in the medical records with a designated staff present.

II – 5.2 Patient Medical Record

Every patient has a sufficiently detailed medical record for the purpose of communication, continuity of care, education, research, evaluation, and medico-legal requirement.

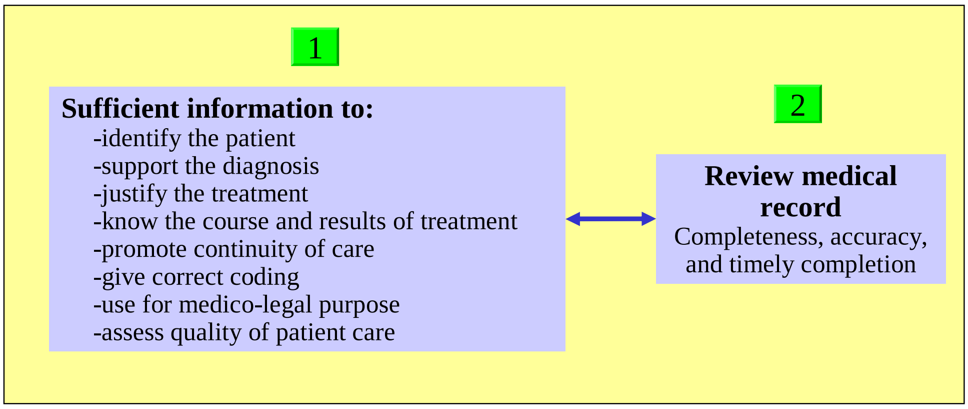

5.2 Patient Medical Record

Every patient has a sufficiently detailed medical record for the purpose of communication, continuity of care, education, research, evaluation, and medico-legal requirement.

(1) The medical record contains sufficient information to:

-identify the patient

-support the diagnosis

-justify the treatment

-know the course and results of treatment

-promote continuity of care

-give correct coding

-use for medico-legal purpose

-assess quality of patient care

(2) The medical records are reviewed periodically for completeness, accuracy, and timely completion.

II-6 Medication Management System

II – 6.1 Medication Planning, Management, and Storage

The organization ensures safety, appropriateness and effectiveness of medication management system and availability of high quality medication.

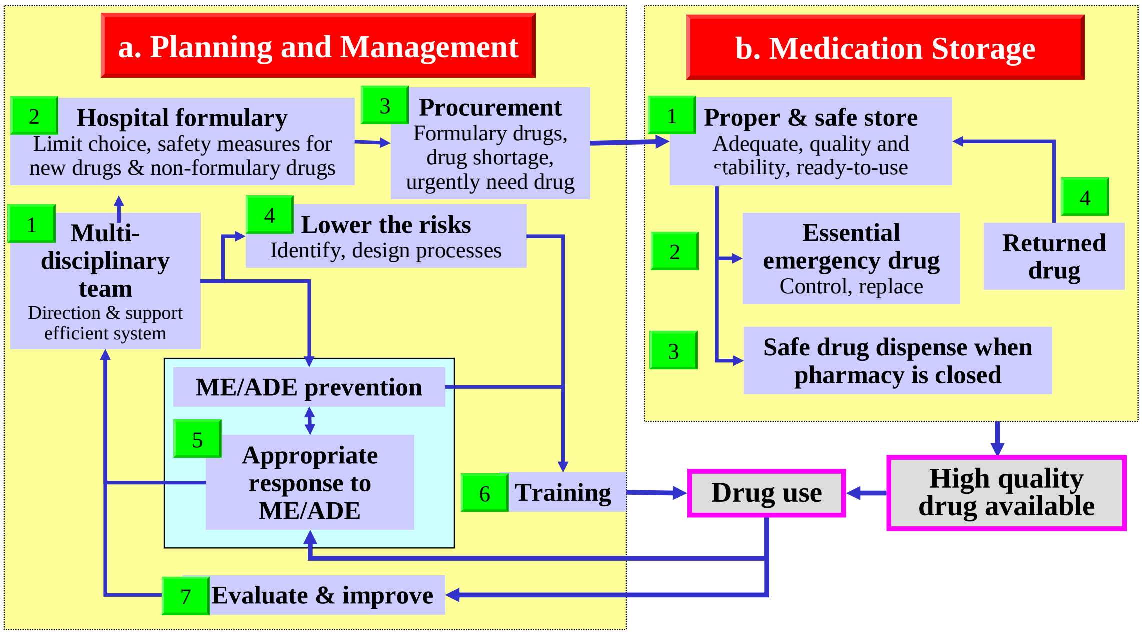

<u>6.1 Planning, Management, and Storage</u>

The organization ensures safety, appropriateness and effectiveness of medication management system and availability of high quality medication.

a. Planning and Management

(1) There is a multidisciplinary team responsible for determining direction and support efficient medication management system.

(2) The hospital formulary is developed to limit choice to essential drugs. The approved formulary is reviewed at least once a year. Safety measures are established for new drugs with heightened error potential, and for the requests to use necessary non-formulary medication.

(3) The procurement is based on the approved formulary. There is a process for handling drug shortage, and drug that is urgently need.

(4) The organization identifies the high-risk or high-alert medications used and design appropriate processes for procuring, storing, ordering, transcribing, preparing, dispensing, administering, and monitoring to lower the risks.

(5)The organization develops and implements policy to prevent medication errors and adverse drug events. The healthcare team responds appropriately to actual or potential adverse drug events and medication errors.

(6) Practitioners undergo competency evaluation and training on knowledge and skills related to medication system, appropriate and safe medication practices at the beginning and then annually.

(7) The organization evaluates and improves its medication management system comparing with its goals. The organization regularly reviews the literature for successful practices or new technologies to improve its medication management system.

b. Medication Storage

(1) All medications are properly and safely stored to ensure adequacy, quality and stability, ready-to-use, prevention of unauthorized access, prevention of medication errors and adverse drug events, tracing to the original sources, with regular inspection of medication storage area throughout the organization.

(2) Essential emergency medication and / or supplies are consistently available in the patient care areas, controlled, secured, and replaced as soon as possible after their use.

(3) There is a safe system for providing medication to meet patient needs when the pharmacy is closed.

(4) Medications returned to the pharmacy are appropriately managed, e.g. discontinued medication

II – 6.2 Medication Use

The organization ensures safety, accuracy, appropriateness and effectiveness in the prescribing and administration of the medication.

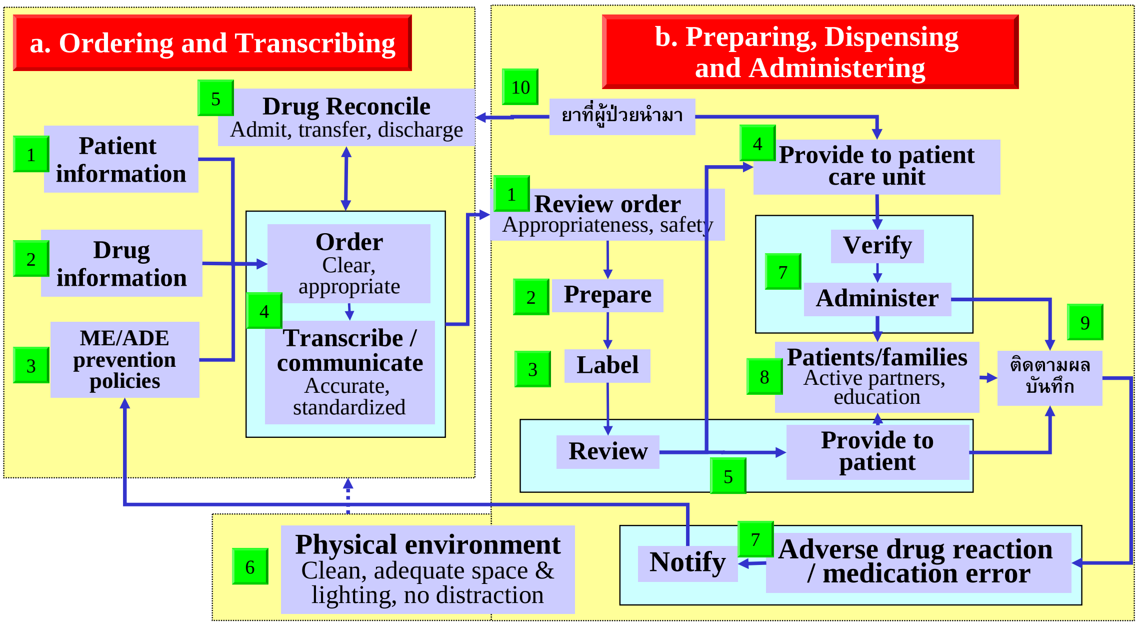

. 6.2 Medication Use

The organization ensures safety, accuracy, appropriateness and effectiveness in the prescribing and administration of the medication.

a. Ordering and Transcribing

(1) Patient-specific information is readily accessible to those involved in the medication management system, including general patient information, a diagnosis or an indication, and necessary laboratory information.

(2) Essential drug information is readily available in useful form and considered when ordering, dispensing, and administering medications.

(3) The organization establishes and implements policies to prevent error and adverse event from ordering and transcribing, including necessary details in prescription, special precaution for look-alike or sound-alike names, measures to prevent the potentially problematic orders and fatal drug interaction.

(4) Medication orders are written clearly and transcribed accurately. Methods of communicating drug orders are standardized to minimize the risk for error. All preprinted order sheets are reviewed and updated on a regular basis.

(5) There is a process of identifying the most accurate list of all medications a patient is taking, and using this list to provide correct medications for patients anywhere within the health care system. The patient's list of current medications is compared against the physician's admission, transfer, and / or discharge orders.

b. Preparing, Dispensing and Administering

(1) All medications prescriptions are reviewed for appropriateness and safety.

(2) Medications are prepared appropriately and safely prepared. Extemporaneous preparation or non-manufacturing drug preparation are prepared by the pharmacy and comply with standard practice.

(3) Medications are appropriately, clearly and legibly labeled at all drug containers. All drug containers taken to the bedside are labeled with at least the patient's name, drug name, strength, and dose.

(4) Medications are provided to patient care units in a safe and secure manner and available for administration within a time frame that meets essential patient needs.

(5) Medications provided directly to patient by pharmacists or designated trained personnel, with appropriate pre-dispensing review and medication advice given to patients.

(6) Medications are prescribed, transcribed, prepared, dispensed, and administered in a clean physical environment that offers adequate space and lighting, and allows practitioners to remain focused on medication use without distractions.

(7) Medications are safely and accurately administered by qualified staff and standardized devices; verifying the correct medication, quality, contraindication, and proper time / dose / route. Prescribers are notified in the event of adverse drug reaction or medication error.

(8) Patients and families are included as active partners in their care through education about their medications for the purpose of accuracy, efficiency, and safety.

(9) The effects of medications on patients are monitored and recorded to assure that medication therapy is appropriate and minimizes the adverse events.

(10) Medications brought into the organization by patients or their families are managed safely and consistently with current patient care plan.

Part II Key Hospital Systems

II - 7 Diagnostic Investigation and Related Services

II – 7.1 Diagnostic Investigation and Related Services: General Requirements

The diagnostic investigation services provide accurate and reliable diagnostic information with minimum hazards to patient and staff.

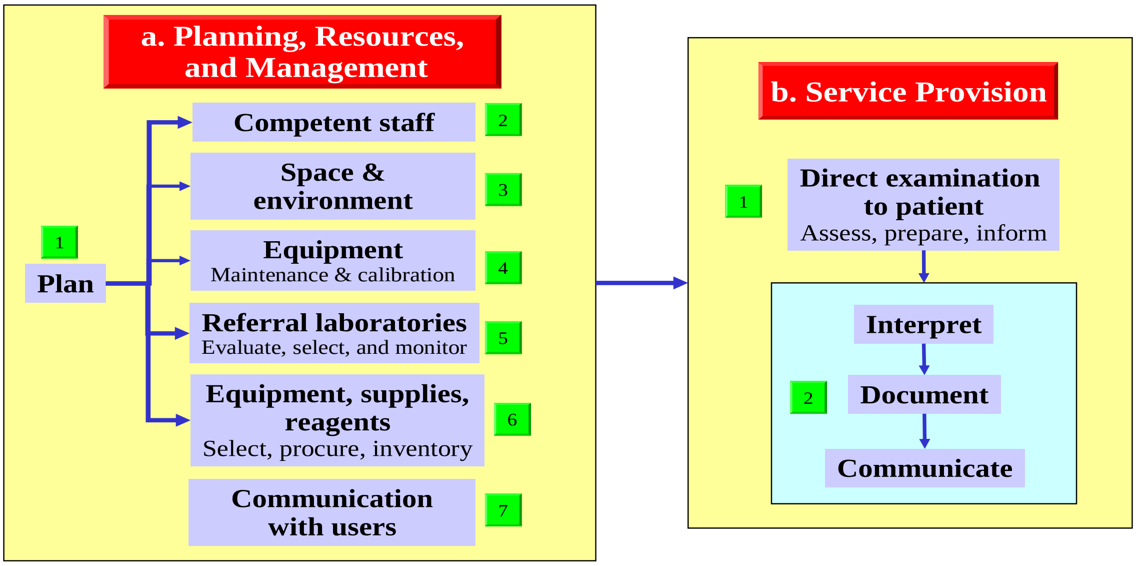

II – 7.1 General Requirements

The diagnostic investigation services provide accurate and reliable diagnostic information with minimum hazards to patient and staff.

a. Planning, Resources, and Management

(1) The services are planned based on the defined goals and user expectation. The plan includes range of services, resource requirements, and expected performance level.

(2) There are adequate and competent staffs to undertake the work required.

(3) The services have adequate space, are designed for the efficiency and safety of their operations; including proper space separation and storage, and attention to the environment that affects the examination results.

(4) The equipment is capable of achieving the performance required, maintained in a safe working condition, with proper preventive maintenance program, calibration and the use of calibration result. The equipment is examined and approved by the responsible authority stated by the law (if any).

(5) The services evaluate, select, and monitor competency and quality of referral laboratories / investigation services and also evaluate consultants who provide second opinion for some specific tests (when necessary, e.g. histopathology and cytology.)

(6) Purchased external services, equipment, and consumable supplies that affect quality of laboratory / investigation services are carefully selected and verified. Suppliers of critical reagents, supplies and services are evaluated. An inventory control system with proper record is established.

(7) There is effective communication with users, both regular meeting and other modes of communication; including advice, interpretation of laboratory / investigation results, consultation on scientific matter, clinical rounds, changing of an examination procedure.

b. Service Provision

(1) For any direct examination to the patient, he / she shall be appropriately assessed (before request and before examination) and prepared to prevent adverse event, minimize patient harm and to ensure the desired quality of examination. In case of high-risk procedures, the patients should be adequately informed and consent signed.

(2) The result of the examination is interpreted by a qualified staff, document a finding summary or a definite diagnosis. The result of the examination is communicated to the responsible physician in a document form and in a timely manner.

II – 7.2 a. Medical Laboratory / Clinical Pathology

The examination processes and quality management system of the medical laboratory ensure reliable and accurate results.

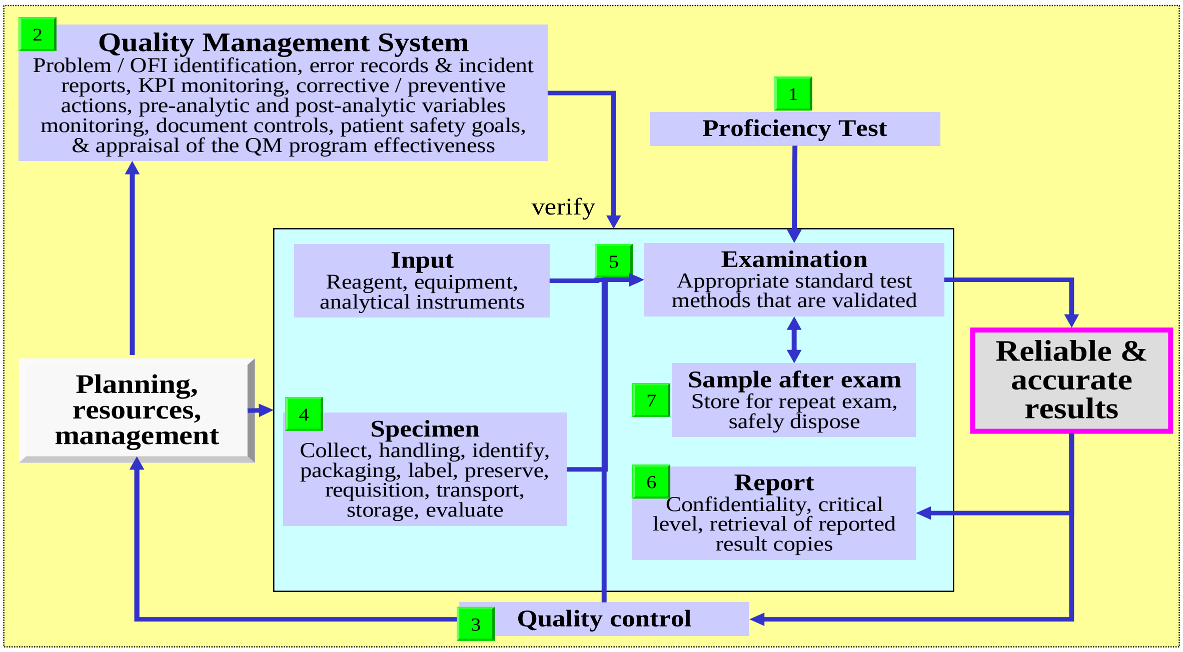

II – 7.2 Additional Specific Requirements

a. Medical Laboratory / Clinical Pathology

(1) The laboratory participates in the proficiency testing (PT) program sufficient for the extent and complexity of the examination done, or exercises an alternative performance assessment system for determining the reliability of the examination. There is evidence that all problems identified by proficiency testing and alternative performance assessment have been corrected promptly.

(2) The laboratory implements the quality management (QM) program / system that covers all aspects of the laboratory service and is coordinated with others in the organization. The QM program / system includes problem / opportunity for improvement identification, error records and incident reports, KPI monitoring, corrective / preventive actions, pre-analytic and post-analytic variables monitoring, document controls, patient safety goals, and appraisal of the QM program effectiveness.

(3) The laboratory implements a quality control program to monitor analytic performance, including establishment of tolerance limits, number and frequency of controls, corrective actions based on quality control data and related information, and procedures to verify the reliability of test results for which neither calibration nor control materials are available.

(4) The specimen collection, handling, identification, packaging, labeling, preservation, accompanied requisition, transportation, and storage are complied with good laboratory practice. Samples are evaluated for acceptance / rejection / interpreting the results. Specimen and sample portion are traceable.

(5) The examination processes ensure reliable and accurate result, using the appropriate standard test methods that are validated for their intended use. The laboratory verifies that purchased reagents, test methods, laboratory equipment, analytical instruments, or products or services meet specified requirements.

(6) The results of examination are accurately and timely reported to the users, considering confidentiality, critical level that may result in patient harm, and retrieval of reported result copies.

(7) The sample is properly managed after examination to enable repetition of the examination when necessary, and safely disposal of sample no longer required for examination.

(8) The highly performed laboratory is encouraged to implement any recognized standard of medical laboratories and apply for assessment from appropriate agencies, such the Department of Medical Science, the Thai Medical Technician Association, or the Royal College of Pathology.

II – 7.2 b. Blood Bank

The organization complies with the Standard of Blood Transfusion and Blood Banking issued by the National Blood Service, Thai Red Cross Society.

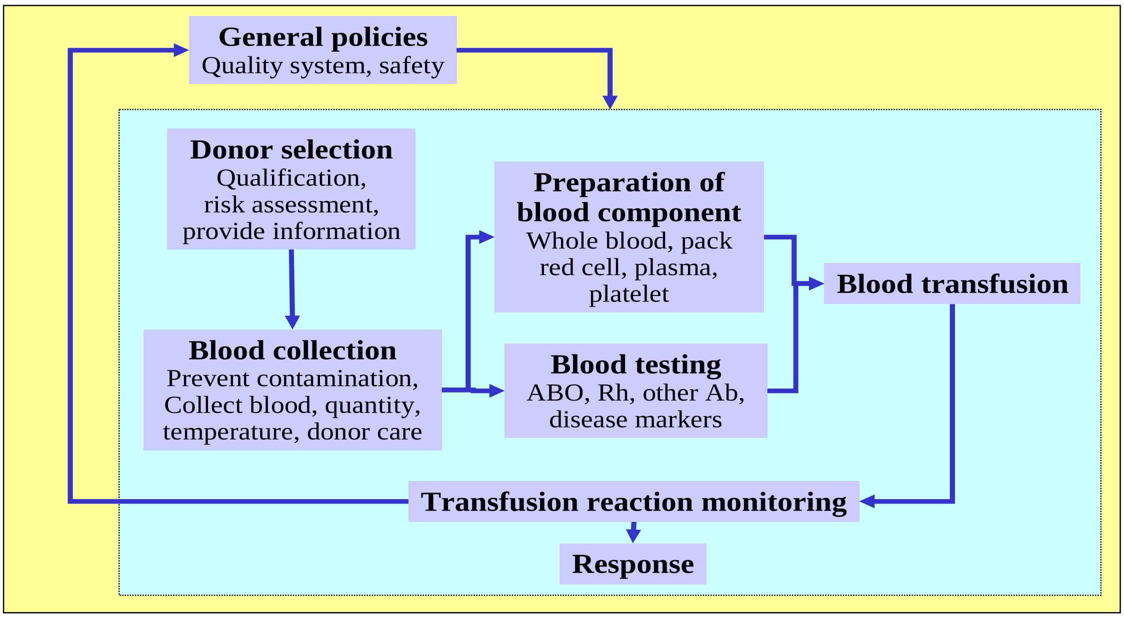

II – 7.2 c. Radiology and Other Medical Imaging Services

The radiology and other medical imaging services provide quality pictures, reliable and accurate results, with minimal hazards to patient and staff.

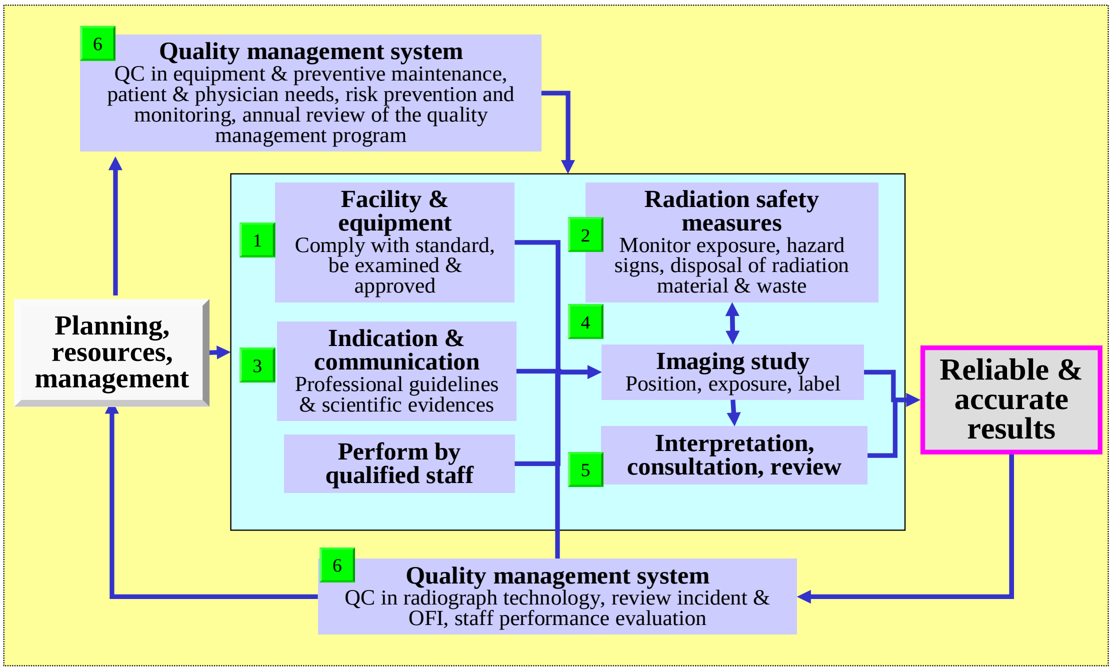

b. Blood Bank

(1) The organization complies with the Standard of Blood Transfusion and Blood Banking issued by the National Blood Service, Thai Red Cross Society.

c. Radiology / Medical Imaging Service

(1) The radiology / medical imaging service has a well-equip facility, of which ensures prevention from radiation hazard for patients, relatives, and staff. The service complies with standards, rules and regulation. The facility and equipment are examined and approved by the responsible authorities specified by a law.

(2) Radiation safety measures are strictly followed. Radiation exposure are monitored and reported. Radiation hazard signs are clearly demonstrated. There is a safe and proper disposal of radiation material and waste.

(3) There is a sufficient clinical indication to request a medical imaging study. The indications should be communicated to the service and the physician responsible for performing and interpretation of the study. The indication and communication is based on professional guidelines and scientific evidence.

(4) All medical imaging studies are accurately performed, accurately and properly labeled. The radiographic positioning and exposure should be standardized and regularly reviewed to optimize imaging quality while minimizing radiation exposure.

(5) The medical imaging studies and related therapies are performed and the interpreted by qualified staff. There is an appropriate consultation with a radiologist and review of the radiographic interpretation (as appropriate).

(6) The service implements a quality management program / system to ensure optimal diagnostic image quality with minimum hazards to patient and staff. The program includes:

- QC in diagnostic imaging equipment and a preventive maintenance program
- QC in radiograph technology
- a process to ensure the needs of patients and referring physicians are being met
- risk prevention and monitoring, e.g. radiation safety, drug safety
- a process to review incidents and opportunities for improvement
- staff performance evaluation

an annual review of the quality management program

II – 8 Disease and Health Hazard Surveillance

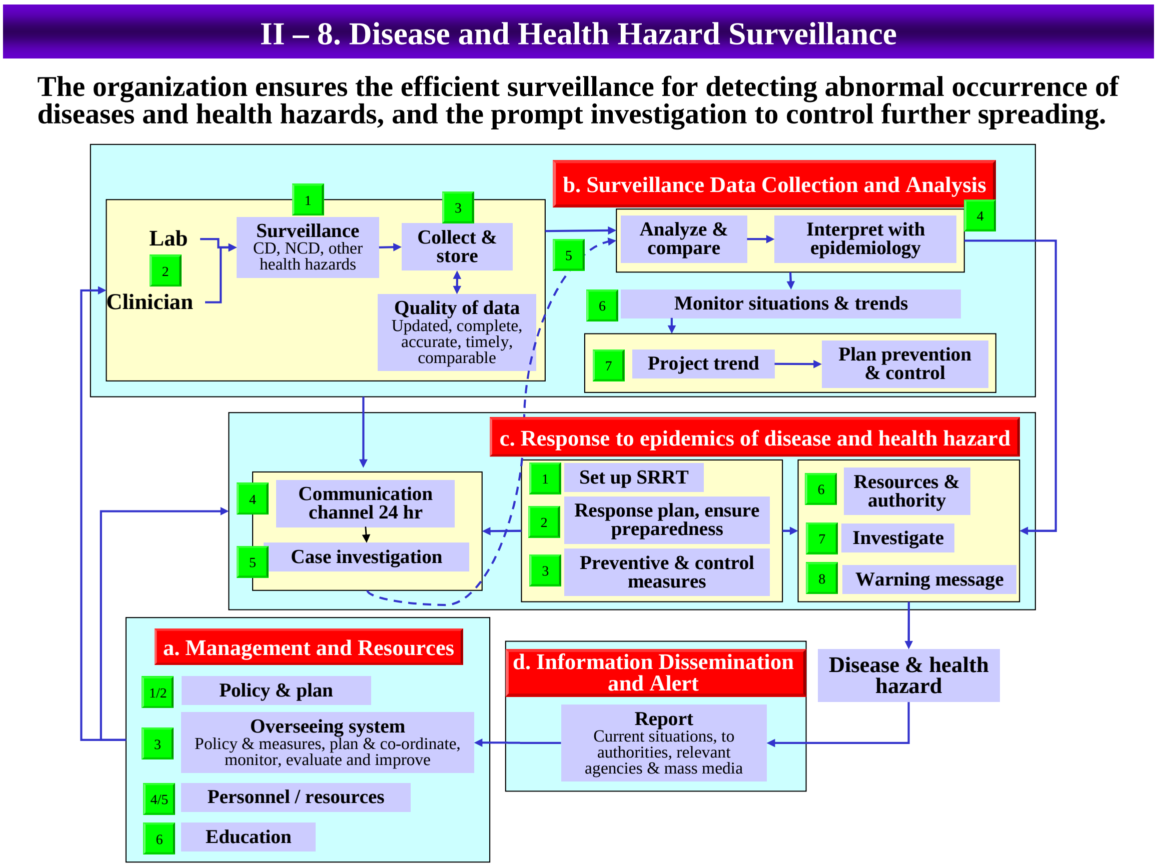

8. Disease and Health Hazard Surveillance

The organization ensures the efficient surveillance for detecting abnormal occurrence of diseases and health hazards, and the prompt investigation to control further spreading.

a. Management and Resources

(1) The disease and health hazard surveillance policy that covers all the areas of service to patients, personnel and public is established.

(2) A clear strategic and action plan relevant to disease and health hazard problems in the service area is established.

(3) A system for overseeing, formulating policy and measures, planning and co-ordination, monitoring, evaluation and improving surveillance procedure is established.

(4) A number of knowledgeable and skillful personnel, as appropriate, are available for operating disease and health hazard surveillance and investigation.

(5) Adequate budget and other resources, and appropriate technologies are available for efficient disease and health hazard surveillance, investigation, and control.

(6) Staff members at all levels are educated and aware of the concept, policy, plans and procedures for disease and health hazard surveillance.

b. Surveillance Data Collection and Analysis

(1) Surveillances for communicable diseases, non-communicable diseases, and other health hazards appropriate to the local health problems are continuously operated, and as required by laws or regulation of the Ministry of Public Health.

(2) Healthcare providers, e.g. physicians, nurses, laboratory technicians, participate in the surveillance process.

(3) Surveillance data is systematically and continuously collected and stored. Data is updated, complete, accurate, timely, and can be compared with previous surveillance data.

(4) Surveillance data is continuously and consistently analyzed, compared and interpreted, using epidemiologic methods.

(5) Detection of an abnormal increase or outbreak of disease is identified by regular surveillance data analysis, receiving information from clinicians, and reviewing laboratory reports.

(6) Situations and trends of diseases under surveillance are continuously monitored and updated.

(7) Trends of important diseases are projected for the purpose of disease prevention and control planning.

c. Response to epidemics of disease and health hazard

(1) A multidisciplinary surveillance and rapid response team (SRRT) is set up for timely response to epidemics of disease & health hazard.

(2) Response plans for public health emergency are established, and ongoing preparedness is ensured.

(3) Essential preventive and control measures for diseases and health hazards are established.

(4) Communication channels and personnel are prepared to receive reports of public health emergency and case patients from clinicians 24 hours a day.

(5) Case investigation is done immediately to prevent a spread of the disease in a timely manner.

(6) Whenever an epidemic occurs, the team responsible for investigation and control has resources and authority to investigate and implement appropriate and comprehensive control measures in a timely manner.

(7) Investigation of epidemic, unusual situation, cluster of illness, or epidemic trend is carried out to prevent widespread epidemic in a timely manner.

(8) Warning messages about the disease and health hazard occurrence are disseminated to communities, governmental agencies and other relevant organizations in a timely manner.

d. Information Dissemination and Alert

(1) Up-to-date reports of the current situation of diseases and health hazards under surveillance, including epidemics and investigations are established.

(2) Cases report is disseminated to the local health authorities and relevant agencies according to the communicable disease act, international health regulations, and other regulations.

(3) Up-to-date information on diseases and health hazards is continuously and consistently disseminated to the relevant agencies and mass media for prevention and control planning.

Part II Key Hospital Systems

II – 9 Working with Community

II – 9.1 Health Promotion for the Community

The healthcare team, in collaboration with the communities, provides health promotion program to meet the need of the communities it serves.

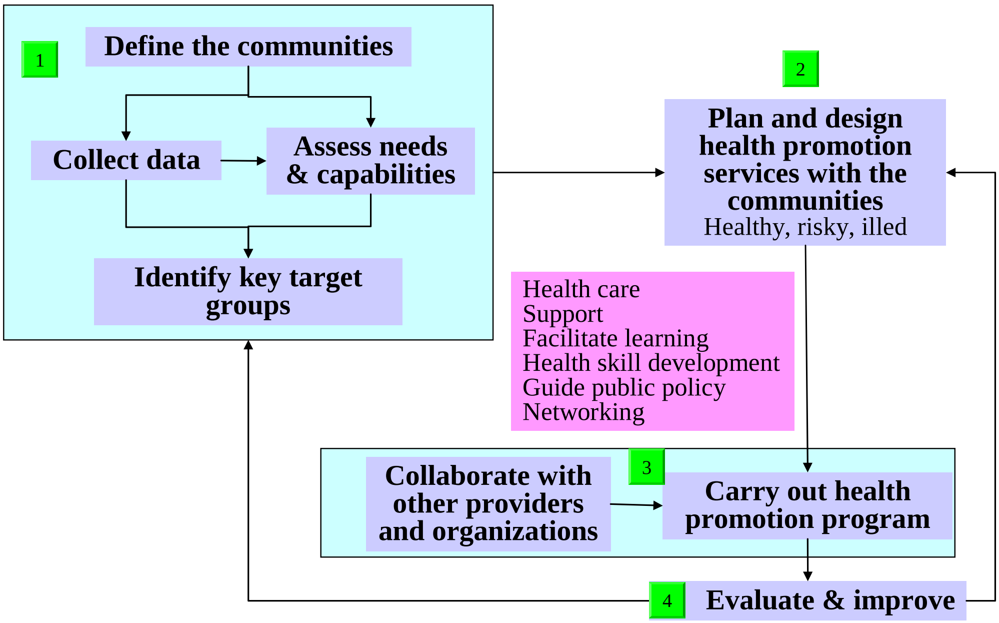

9.1 Health Promotion for the Community

The healthcare team, in collaboration with the communities, provides health promotion program to meet the need of the communities it serves.

(1) The healthcare team defines communities it serves, assesses the needs and capabilities of the communities it serves, and identifies key target groups.

(2) The healthcare team, in collaboration with the communities, plans and designs its health promotion services to meet the needs and problems of the communities.

(3) The healthcare team, in collaboration with other providers and organizations, carries out health promotion program for the communities.

(4) The healthcare team evaluates and improves health promotion program for the communities.

II – 9.2 Community Empowerment

The healthcare team works with the communities to support the development of the communities' capacities to improve its own health and well-being.

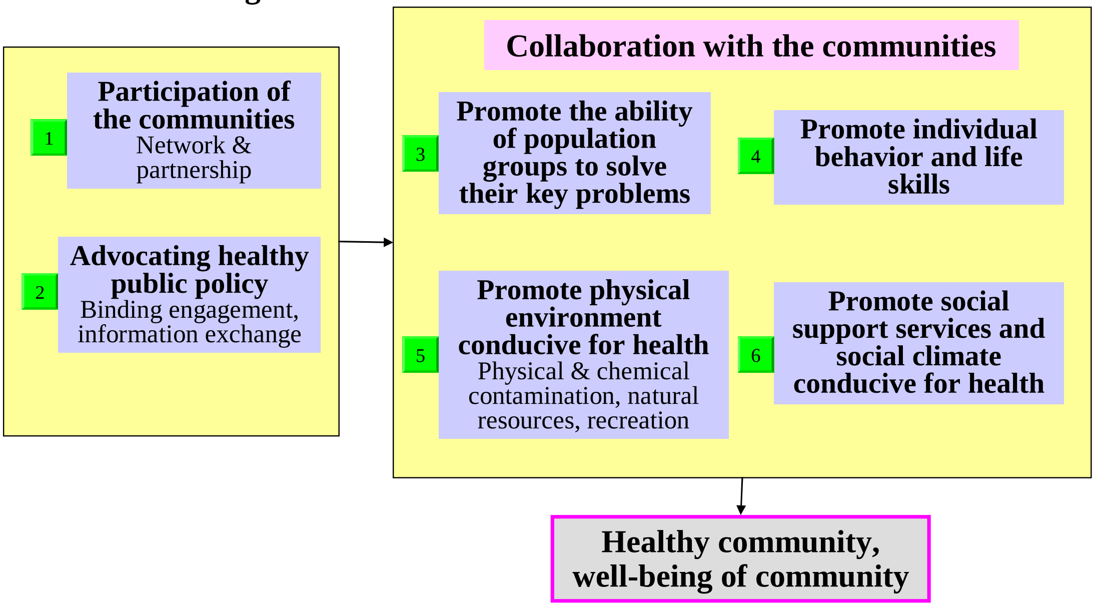

9.2 Community Empowerment

The healthcare team works with the communities to support the development of the communities' capacities to improve its own health and well-being.

(1) The organization promotes participation, mobilizes the network, and strengthens partnership with the communities.

(2) The organization takes part in advocating healthy public policy through binding engagement of key persons and information exchange.

(3) The organization, in collaboration with the communities, promotes the ability of population groups to find solutions for key problems identified by the communities.

(4) The organization, in collaboration with the communities, promotes individual health-related behavior and life skills (including knowledge, attitude, beliefs, values) that are significant for one's own health.

(5) The organization, in collaboration with the communities, promotes physical environment conducive for health in the community.

(6) The organization, in collaboration with the communities, promotes social support services and social climate conducive for health.

PART III PATIENT CARE PROCESSES

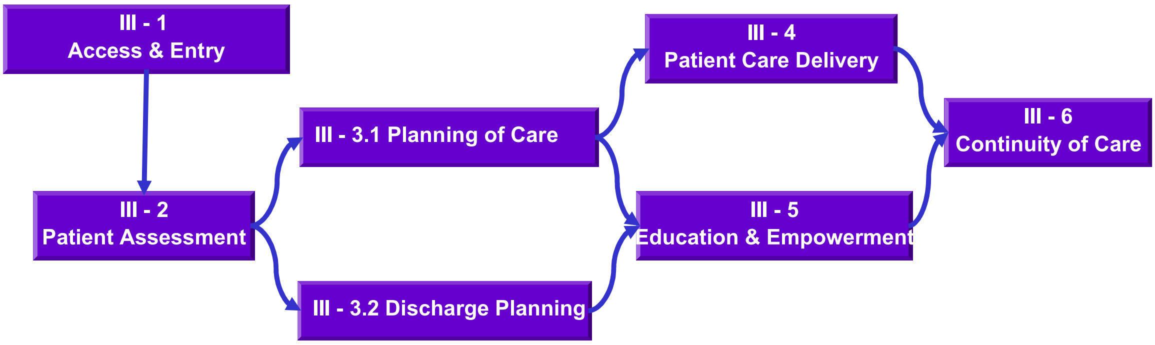

III – 1 Access and Entry

III –1. Access & Entry

The healthcare team ensures access to essential services with minimal barrier, ensures that an entry process is timely, meets patient problems / needs, is coordinated and supported by suitable and effective systems and environment.

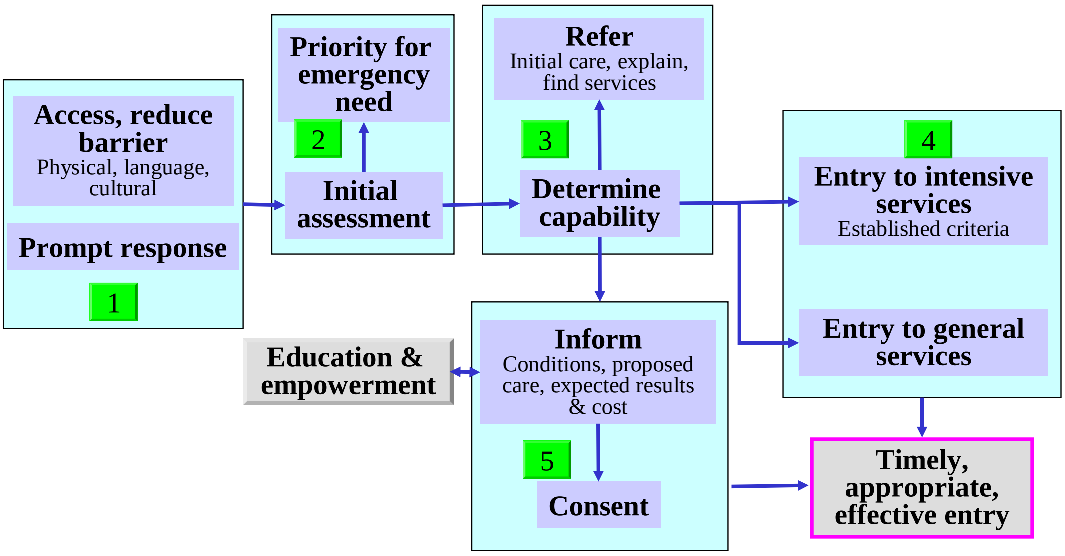

III – 1 Access and Entry

The healthcare team ensures access to essential services with minimal barrier, ensures that an entry process is timely, meets patient problems / needs, is coordinated and supported by suitable and effective systems and environment.

(1) Communities are able to access a range of services that are appropriate to their needs. The healthcare team seeks to reduce physical, language, cultural, and other barriers to access of services. The healthcare team responds promptly to those who ask for services. Waiting times for services are acceptable to the communities they serve.

(2) Patients with emergency or immediate needs are given priority for assessment and treatment.

(3) Capability to offer services or accept patient is determined, using the established criteria. If the healthcare team cannot provide services to the patient, it provides appropriate initial care, explains the reasons why, and helps patients to find other more appropriate services.

(4) Transfer to or entry to units providing intensive or specialized services is determined by established criteria.

(5) At admission, patients / families are given clear, understandable and appropriate information about their conditions, the proposed care, the expected results of that care, any expected cost. Informed consent is obtained before starting any service or intervention, ensuring that patients / families receive and understand necessary information, have time to consider before making decision, and proper records are made.

III - 2 Patient Assessment

III – 2. Patient Assessment

All patients have their healthcare needs and problems identified accurately in a comprehensive and appropriate manner.

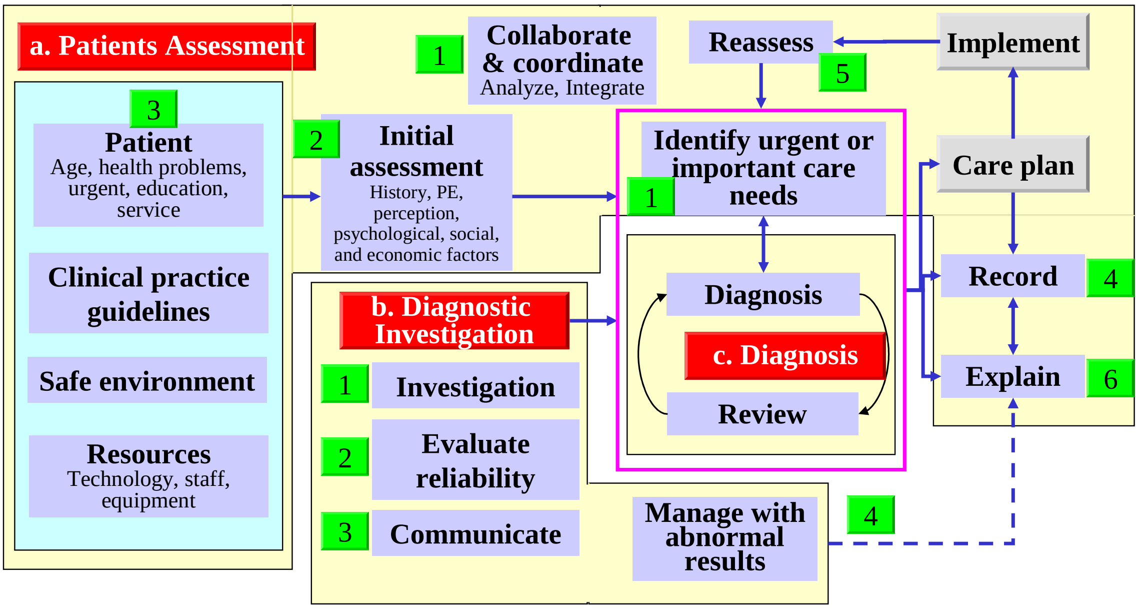

III - 2 Patient Assessment

All patients have their healthcare needs and problems identified accurately in a comprehensive and appropriate manner.

a. Patients Assessment

(1) A comprehensive patient assessment is coordinated to reduce unnecessary repetition. Professionals responsible for patient care collaborate to analyze and integrate patient assessments. The most urgent or important care needs are identified.

(2) Each patient's initial assessment includes: health history and physical examination, patient's perception of his / her needs, an evaluation of psychological, social, and economic factors.

(3) The assessment method is appropriate with each patient, under a safe environment and adequate resources. When available, clinical practice guidelines that are appropriate to the patients and resources are used to guide patient assessment.

(4) Patients are assessed within the time frame established by the organization. Assessment findings are documented in the patient's record and readily available to those responsible for the patient's care.

(5) All patients are reassessed at appropriate intervals to determine their response to treatment.

(6) The healthcare team shares the assessment results with patients and families in a clear and easy-to-understand way.

b. Diagnostic Investigation

(1) Essential diagnostic investigation is provided or referred to other facilities in a timely manner.

(2) The reliability of diagnostic investigation results is evaluated, comparing with patient's condition.

(3) The diagnostic investigation results are effectively communicated and documented; ensuring that physicians get results in a timely manner, results are easily retrieved, without loss, and with proper confidential precaution.

(4) There is an attempt to explain or further investigate if the investigation results are abnormal.

c. Diagnosis

(1) The patients receive correct diagnosis with enough documented evidence to support the diagnosis. The diagnosis is recorded in the determined period, and is updated when there is more information. The appropriateness of diagnosis and consistency of diagnosis by each profession in the healthcare team is reviewed regularly.

III-3 Planning

III – 3.1 Planning of Care

The healthcare team ensures a coordinated patient care plan with goals developed in response to health problems / needs of the patient.

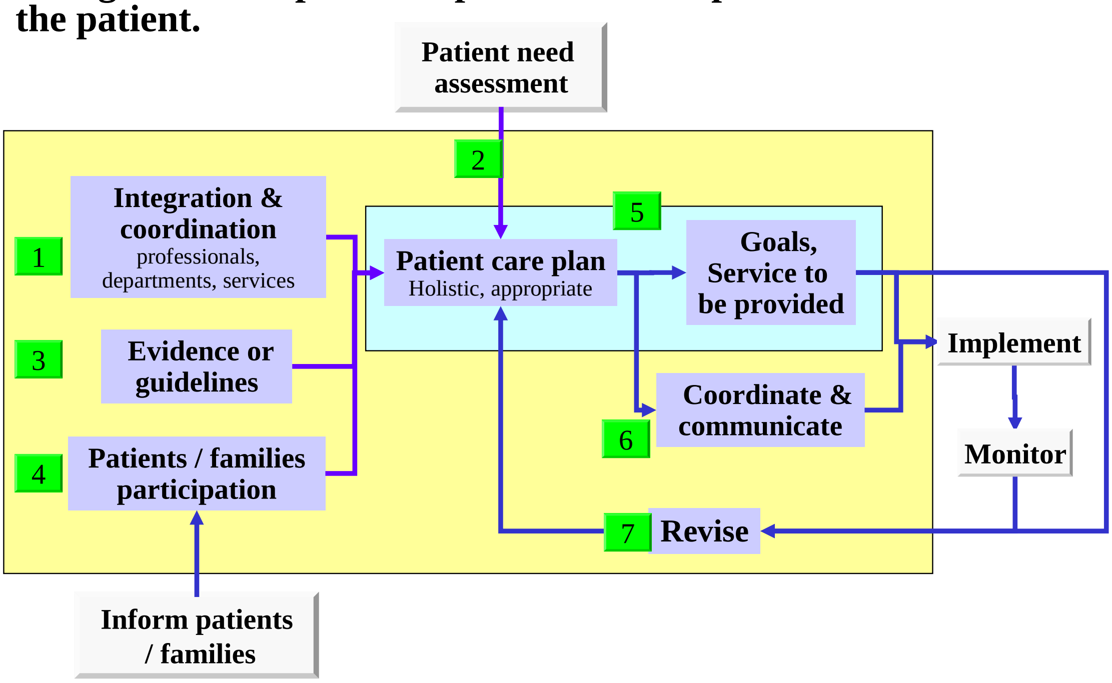

Part III   Patient Care Provessess

3.1 Planning of Care

The healthcare team ensures a coordinated patient care plan with goals developed in response to health problems / needs of the patient.

(1) Patient care planning is integrated and coordinated among professionals, departments, and services.

(2) The patient care plan is based on patient's needs identified in the assessment process in a holistic way.

(3) Appropriate evidence or guidelines are used to guide the patient care plan.

(4) Patients and families have opportunities to make informed decision on choice of treatment and participate in the planning of care.

(5) The patient care plan includes the goals to be achieved and service to be provided.

(6) The care plan is effectively coordinated and communicated to all team members and relevant services in a timely basis, and the team members understand the role of each other.

(7) The patient care plan is revised when indicated by a change in the patient's condition.

III – 3.2 Discharge Planning

Discharge planning process enables self care of patients and ensures that patient's ongoing needs after discharge are met.

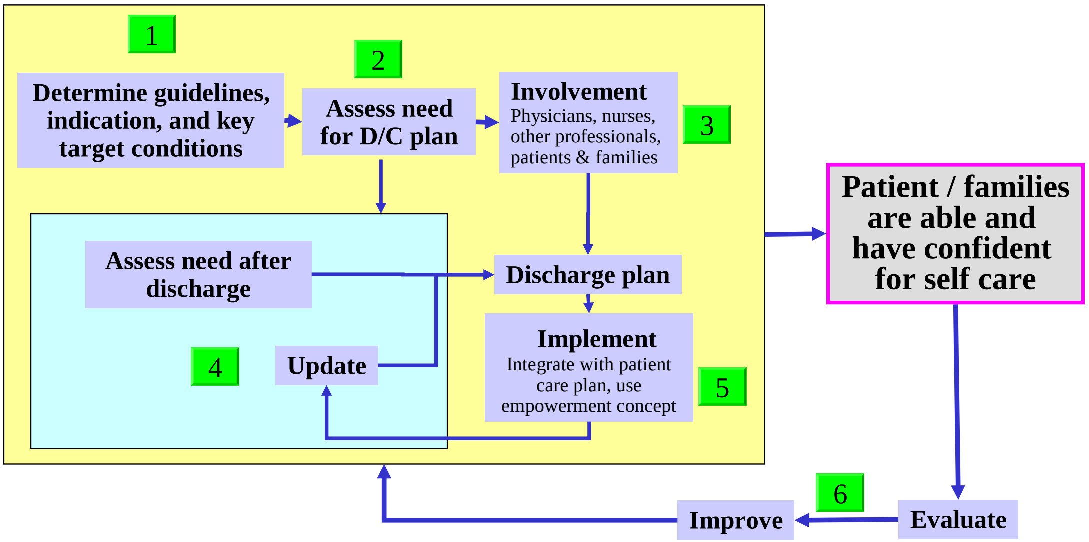

3.2 Discharge Planning

Discharge planning process enables self care of patients and ensures that patient's ongoing needs after discharge are met.

(1) Guidelines, indication, and key target conditions for discharge planning are determined.

(2) The need for discharge planning of an individual patient is determined early in the care process.

(3) Physicians, nurses, other relevant professionals, including patients and their families, are involved in discharge planning.

(4) Patient's needs after discharge are assessed and updated throughout the hospital stays.

(5) Discharge plan is implemented in an integrated manner with patient care plan, using empowerment concept to ensure that patients and families have capability and confidence in management of self-care.

(6) The discharge planning process is evaluated and improved using information from patients' follow up and feed back from other relevant health service providers.

III-4 Patient Care Delivery

III - 4.1 General Care Delivery

The healthcare team ensures that care is delivered in a timely, safe, and appropriate manner according to professional standards.

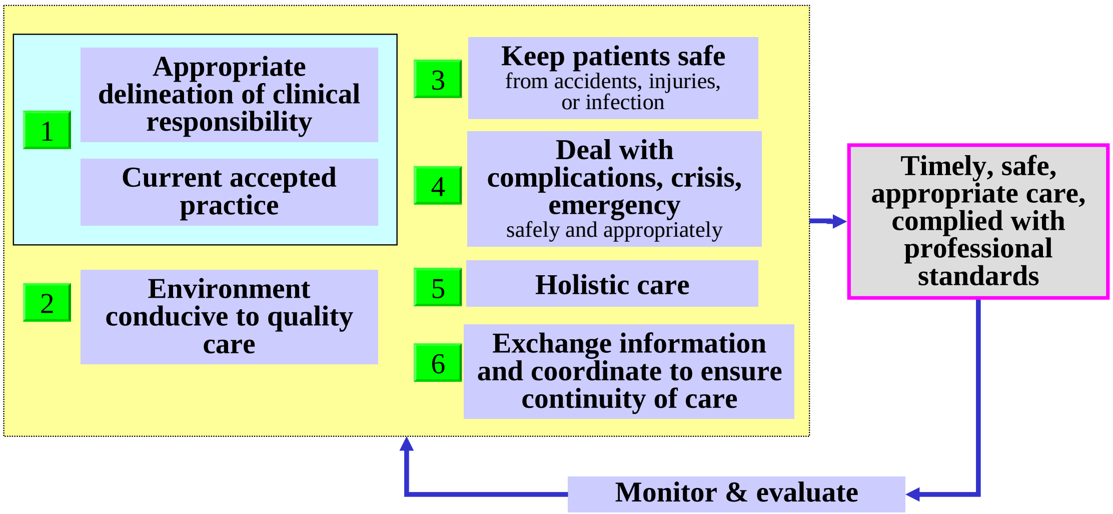

Part III Patient Care Provessess

4.1 General Care Delivery

The healthcare team ensures that care is delivered in a timely, safe, and appropriate manner according to professional standards.

(1) Care is delivered in a timely, safe, and appropriate manner with appropriate delineation of clinical responsibility. The care meets current accepted practice throughout the organization.

(2) Care environment is conducive to the provision of quality care, considering patient dignity and privacy, pleasant and clean surroundings, prevention of hazards / stress / noise / other disturbance.

(3) The care team takes all reasonable steps to keep patients safe from accidents, injuries, or infection.

(4) The care team has a process for safely and appropriately dealing with complications, a crisis, or an emergency.

(5) The care team response to the patient's need in a holistic way, especially emotional and psychosocial problems.

(6) The care team exchanges information and coordinates to ensure continuity of care.

III - 4.2 Care of High-Risk Patients and Provision of High-Risk Services

The healthcare team ensures that care of high-risk patients and high-risk services is delivered in a timely, safe, and appropriate manner according to professional standards.

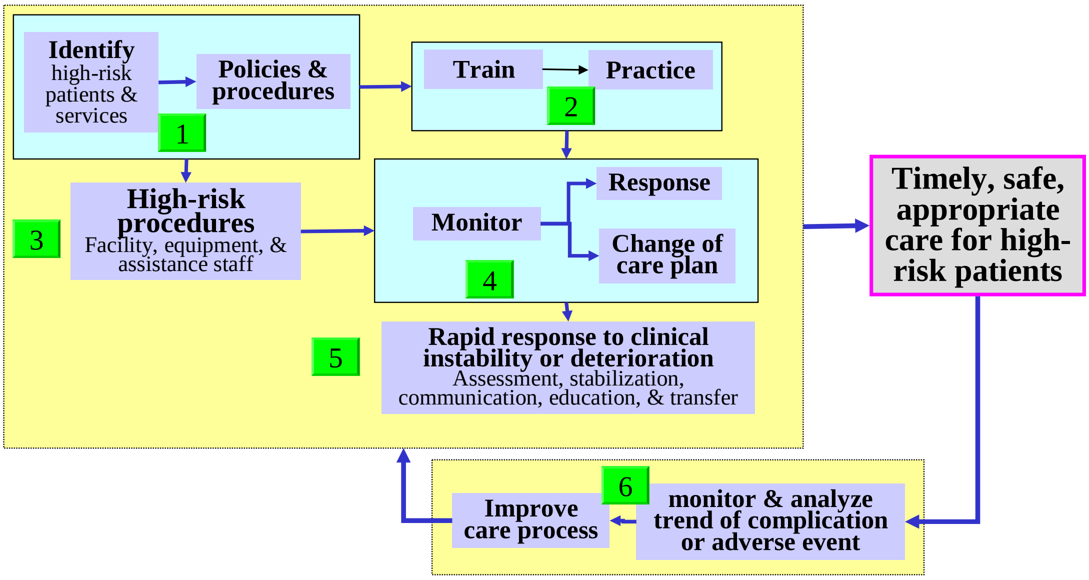

4.2 Care of High-Risk Patients and Provision of High-Risk Services

The healthcare team ensures that care of high-risk patients and high-risk services is delivered in a timely, safe, and appropriate manner according to professional standards.

(1) The healthcare team identifies the high-risk patients and high-risk services, and collaboratively develops applicable policies and procedures for caring of these high-risk conditions.

(2) Staffs have been trained and use the policies and procedures to guide care of these high risk conditions.

(3) The high-risk procedures must be performed in an appropriate facility, with available essential equipment and assistance staff.

(4) The high-risk patients or patients receiving high-risk services are monitored as appropriate to the patients' condition, with timely response or change of care plan.

(5) When there is a sign of clinical instability or deterioration, assistance by a more expert staff or health care team can be made in a timely manner for patient assessment, stabilization, communication, education, and transfer (if necessary).

(6) The care team monitors and analyzes trend of complication or adverse event in these patients to improve the patient care processes.

4.3 Specific Care

The healthcare team ensures that key specific services are delivered in a timely, safe, and appropriate manner according to professional standards.

a. Anesthesia Care

(1) A pre-anesthesia assessment is performed to identify any potential risk that may occur during anesthesia, of which information is used for planning of appropriate anesthesia and consultation with relevant specialist.

(2) Patients / families receive essential information about anesthesia, participate in decision making on choice of anesthesia, if possible. Pre-anesthesia preparation, both physical and mental, is performed.

(3) The anesthetic process is carried out smoothly and safely, based on the professional standards best fit for the hospital situation, by qualified staff.

(4) During anesthesia and post-anesthesia, each patient's physiological status is monitored and documented. It is well prepared to deal with emergency situation during anesthesia and recovery. A qualified individual discharges the patient from the recovery area using established criteria.

(5) Equipment, supplies, and medications recommended by anesthesia professional organization are used.

b. Surgical Care

(1) Each patient's surgical care is planned. The planning process considers all available assessment information. The surgical care plan is documented in the patient's record, including a preoperative diagnosis. The care team assesses patient's risks and coordinated with relevant professionals for a safe care.

(2) The rational, options of surgical procedures and blood use, risks, potential complications are discussed with the patient, family, or those who make decisions for the patient.

(3) The elective and emergency patients receive preoperative preparation, both physically and psychologically, for patient readiness, and reduction of surgical risks and infection. There is an appropriate measure to prevent wrong patient and wrong site surgery.

(4) Surgical care is undertaken under well-prepared, efficient, and safe condition.

(5) The surgery performed is written in the patient's record on a timely basis to support communication among the care team and support a continuum of post-surgical care.

(6) The monitoring and care after surgery is appropriate to the patient's condition and the procedure performed.

c. Food and Nutrition Therapy

(1) Patients receive appropriate food, with adequate nutritional value for their basic need, through a good food service.

(2) Patients at nutrition risk receive nutritional assessment and planning, and receive adequate nutritional therapy.

(3) Patients and families are educated on food, nutrition, and nutritional therapy to enable them for behavioral change, food preparation and consumption that are appropriate with their illnesses.

(4) Food preparation, storage, delivery, and handling of dishes / equipment / waste / residual food are safe and comply with acceptable practices to reduce the risk of contamination, spoilage, vectors, and spreading of diseases.

d. End-Of-Life Care

(1) Staffs are aware of patients' unique needs at the end of life.

(2) Patients / families are assessed / reassessed for symptoms, response to symptom management, psychosocial and spiritual needs.

(3) The care team ensures appropriate care of those dying by taking intervention to manage symptoms; taking intervention that address psychosocial, emotional, and spiritual needs; involving the patient and family in care decisions.

e. Pain Management

(1) Patients are assessed for pain. When pain is identified, a comprehensive pain assessment is performed to measure pain intensity and quality.

(2) The care team ensures appropriate pain management.

f. Rehabilitation Service

(1) A rehabilitation plan, based on assessment of the patient's physical, emotional, and social status, is developed to guide rehabilitation services to reach personal rehabilitation goals.

(2) Rehabilitation restores, improves, or maintains the patient's optimal level of functioning, self-care, self-responsibility, independence, and quality of life.

III-5 Information and Empowerment for Patient / Family

III - 5. Information and Empowerment for Patients / Families

The healthcare team provides patients and families with information on their health condition and planned activities to empower them, encourages them to carry out their responsibilities, and to facilitate integration of health promotion in all patient pathways.

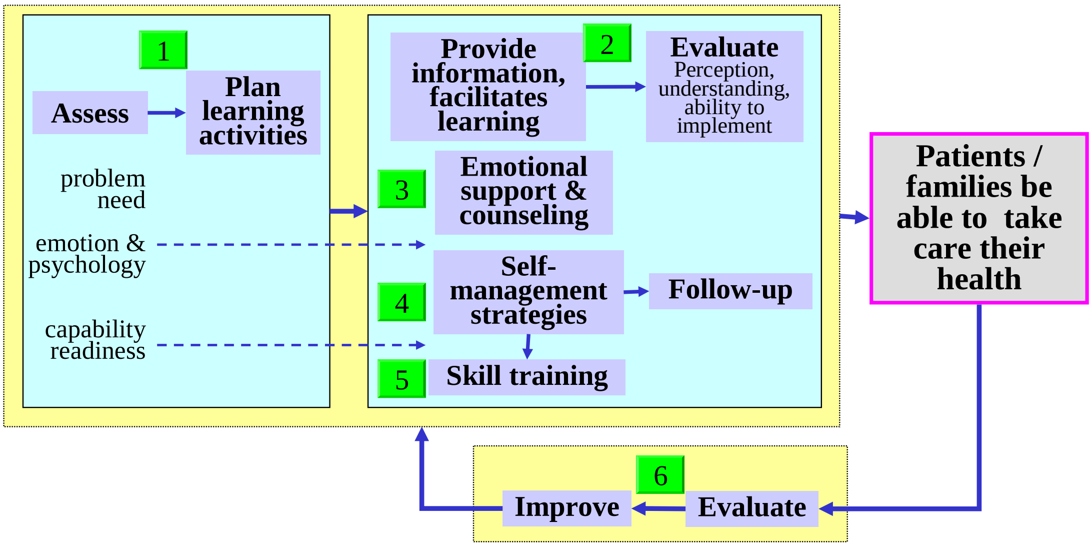

III-5 Information and Empowerment for Patient / Family

The healthcare team provides patients and families with information on their health condition and planned activities to empower them, encourages them to carry out their responsibilities, and to facilitate integration of health promotion in all patient pathways.

(1) The healthcare team assesses patients to plan and determine learning activities. The assessment includes patients' problems and needs, capability, emotional and psychological condition, readiness for learning and self-care.

(2) The healthcare team provides essential information and facilitates learning for self-care and good health behavior to patients and families. Such information and learning is appropriate for patient's problem, timely, clear and understandable. Perception, understanding, ability to implement are evaluated.

(3) The healthcare team provides appropriate emotional support and counseling to help patients and families.

(4) The healthcare team and patients / families, collaboratively determine appropriate self-management strategies, including continuously follow up on problems and difficulties in self-care

(5) The healthcare team provides essential skill training for patients / families, and ensures that patients / families are able to do by themselves.

(6) The healthcare team evaluates and improves the effectiveness of health education, learning and empowerment program.

III-6 Continuity of Care

III - 6 Continuity of Care

The healthcare team collaborates and co-ordinates for effective follow-up and continuity of care.

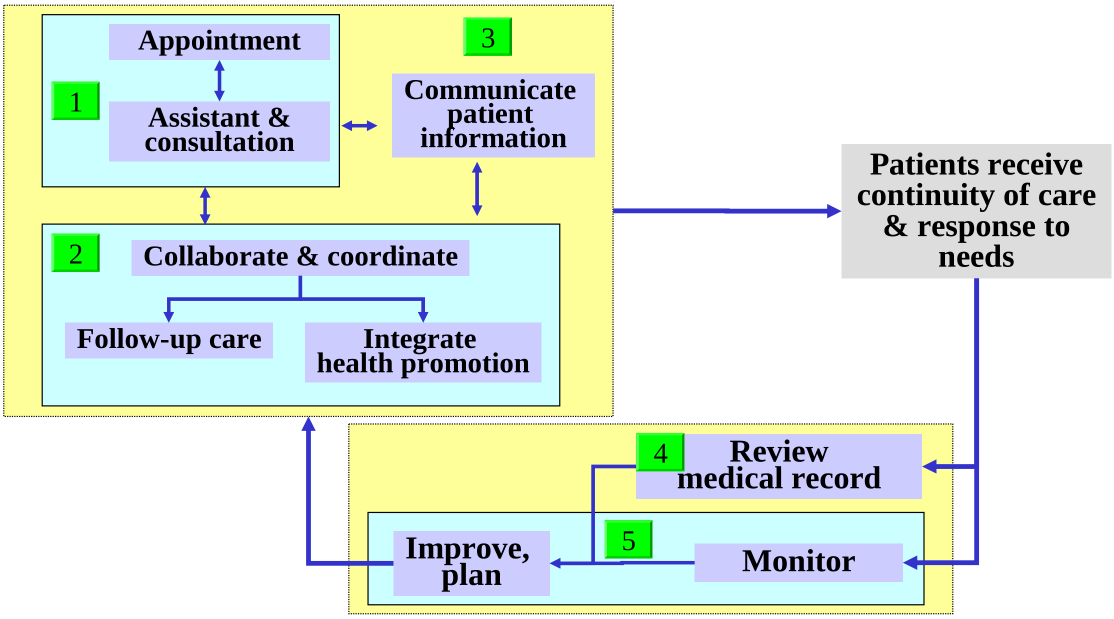

III-6 Continuity of Care

The healthcare team collaborates and co-ordinates for effective follow-up and continuity of care.

(1) When indicated, appointment is made for follow up care. Assistant and consultation for the discharged patients are arranged as appropriate.

(2) The organization collaborates and co-ordinates with other relevant health care providers, organizations, communities, and other sectors to allow continuity of follow-up care and integrate health promotion activities in patient pathways.

(3) Patient information is effectively communicated to all relevant health care providers in the continuum of care, both inside and outside the organization, considering confidentiality of patient information.

(4) Medical records are reviewed to ensure the documentation facilitate continuing care.

(5) Patient results are continuously monitored to ensure that patients' needs are met and the information is used for improvement and planning future services.

PART IV ORGANIZATION PERFORMANCE RESULTS

The organization demonstrates good performance and improvement in key area, i.e. health care results, patient and other customer-focused results, financial results, human resource results, process effectiveness results, leadership results, and health promotion results.

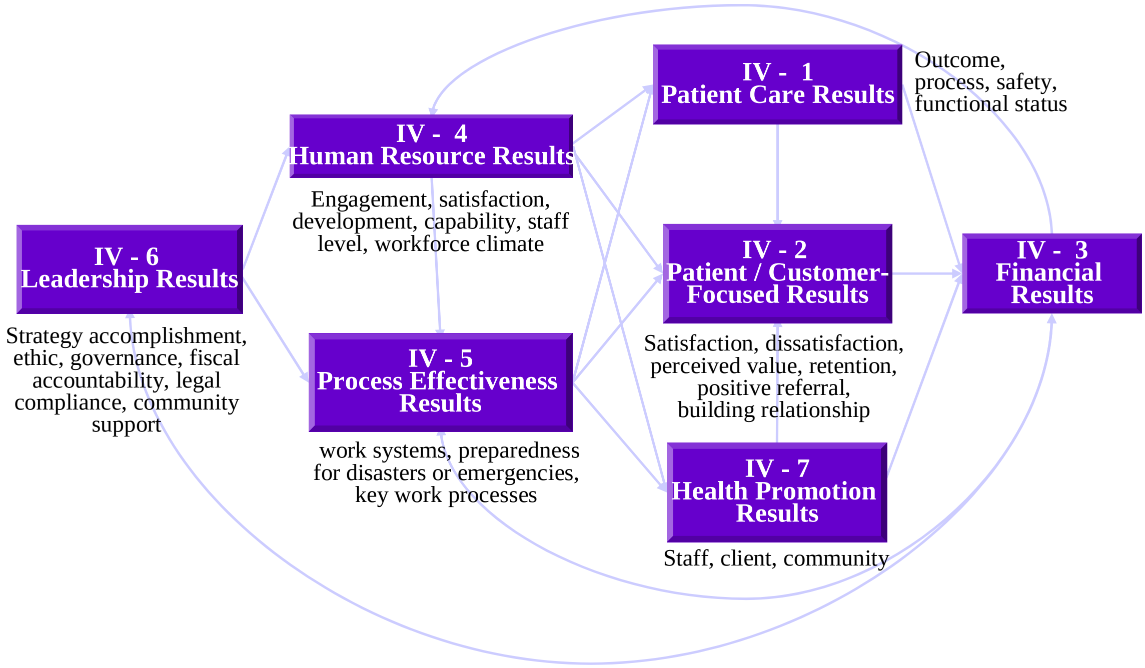

IV-1 Patient Care Results

The organization demonstrates the current levels and trends in key indicators of patient care outcome, patient care process, patient safety and patients' functional status.

IV-2 Patient and Other Customer-Focused Results

The organization demonstrates the current levels and trends in key indicators of patient and other customer satisfaction; dissatisfaction; perceived value; retention, positive referral, and building relationship with customers.

IV-3 Financial Results

The organization demonstrates the current levels and trends in key indicators of financial performance.

IV-4 Human Resource Results

(1) The organization demonstrates the current levels and trends in key indicators of staff engagement, staff satisfaction, and the development of staff and leaders.

(2) The organization demonstrates the current levels and trends in key indicators of staff capability, staffing level, retention and appropriate skill.

(3) The organization demonstrates the current levels and trends in key indicators of workforce climate, health, safety, security, services and benefits.

IV-5 Key System & Process Operational Results

(1) The organization demonstrates the current levels and trends in key indicators of the operational performance of the work systems, including preparedness for disasters or emergencies.

(2) The organization demonstrates the current levels and trends in key indicators of the operational performance key work processes.

IV-6 Leadership Results

(1) The organization demonstrates the current levels and trends in key indicators of accomplishment of organizational strategy and action plans.

(2) The organization demonstrates the results of ethical behavior and trust in the senior leadership and governance of the organization.

(3) The organization demonstrates the results of fiscal accountability, both internal and external.

(4) The organization demonstrates the current levels and trends in key indicators of organizational assessment, regulatory and legal compliance.

(5) The organization demonstrates the current levels and trends in key indicators of organizational citizenship in support of its communities, including contributions to the health of community.

IV-7 Health Promotion Results

(1) The organization demonstrates the current levels and trends in key indicators of health behavior and health outcome of staff.

(2) The organization demonstrates the current levels and trends in key indicators of health behavior and health outcome of certain key client group.

(3) The organization demonstrates the current levels and trends in key indicators of health behavior and health outcome of people in the communities.
